# Glutathione‐Responsive Acyl‐Modifications for Targeted RNA Decaging and Prolonged Protein Synthesis

**DOI:** 10.1002/anie.8835235

**Published:** 2026-07-17

**Authors:** Mary E. Flood, Sathishkumar Kurusamy, Mark Berney, C. Mark Smales, Elizabeth M. Topp, Joanna F. McGouran, Steven Ferguson

**Affiliations:** ^1^ RINN Pharma and Biopharma Centre School of Chemical and Bioprocess Engineering University College Dublin, Belfield Dublin Ireland; ^2^ RINN Pharma and Biopharma Centre National Institute for Bioprocess Research and Training, Mount Merrion Dublin Ireland; ^3^ Industrial Biotechnology Centre School of Natural Sciences University of Kent Canterbury UK; ^4^ School of Chemistry and Chemical Engineering Queen's University Belfast Belfast UK; ^5^ Department of Industrial and Physical Pharmacy Purdue University West Lafayette Indiana USA; ^6^ RINN Pharma and Biopharma Centre School of Chemistry and Trinity Biomedical Sciences Institute Trinity College Dublin The University of Dublin Dublin Ireland

**Keywords:** acyl‐modification, decaging, glutathione‐responsive, mRNA, RNA

## Abstract

A series of disulfide‐based, self‐immolative acyl conjugates have been designed and tested for reversibly protecting the ribose 2’‐hydroxy position of RNA. These modifications were found to be responsive to endogenous glutathione levels for efficient RNA recovery both in solution and cultured cells. Through this modification strategy, we achieve shielding of the biomolecule from both nuclease enzymes and translational machinery, allowing for a tunable release of RNA with improved cellular stability and minimal innate immune activation. The self‐immolative RNA deprotection includes a cyclization step which exhibits differing kinetic profiles based on ring size and electronic properties. Moreover, we demonstrate sustained target protein production for both green‐fluorescent protein and nanoluciferase in cell models. Both acylated mRNA models resulted in significant increases in protein expression compared to the unmodified mRNA control, with increases in protein expression of up to 600% and sustained higher protein expression of modified mRNA samples observed over 120 h in comparison to unmodified RNA. These exciting results highlight the versatility of our approach in the design of next‐generation RNA‐based prodrugs.

## Graphical abstract

The self‐immolation of disulfide‐based mRNA modifications in response to endogenous glutathione (GSH) promotes a gradual release of translatable mRNA within the cell, leading to improved nuclease resistance, tunable release properties, and a significant increase (up to 600%) of target protein production over time. This strategy offers an exciting proof of principle toward next‐generation, higher‐efficacy mRNA‐based prodrugs.

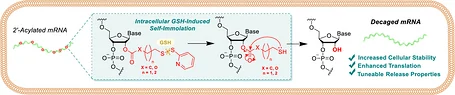

## 1 Introduction

The inherent diversity of RNA as a biotherapeutic molecule has been widely showcased across multiple applications including protein production, target protein binding, gene knockdown, and CRISPR‐based gene editing [1, 2]. Through modulation of the nucleobase, ribose, phosphate backbone, or chain end (3’ or 5’), further control of RNA activity can be achieved [3, 4, 5, 6, 7]. In recent years, post‐synthetic strategies have been employed to selectively modify the 2’‐hydroxy (2’‐OH) position of the RNA ribose ring [8, 9]. A route toward 2’‐OH protection has benefits in preventing intramolecular attack of this functional group on the neighboring phosphodiester, leading to reduced degradation of therapeutic RNA alongside the improvement of its pharmacokinetic properties [10, 11].

Several “on‐off” prodrug‐type strategies have been reported for protection of the 2’‐hydroxy unit of RNA [12]. Such approaches include a diverse library of acyl imidazole [8, 10, 11, 13, 14, 15], isatoic anhydride [16, 17, 18], acyl cyanide [19], activated NHS ester [20], sulfonyl [21], halogenated [9], and heterocyclic species [22]. Under aqueous conditions, these reagents react stochastically with the 2’‐OH position of RNA to produce acylated strands of the biomolecule (Acyl‐RNA) [12]. Advantages of post‐synthetic and post‐transcriptional RNA modifications include ease of preparation and incorporation, circumventing the need for non‐standard phosphoramidite coupling reactions during solid‐phase oligonucleotide synthesis, or enzymatic recognition of non‐natural nucleotide triphosphates during in vitro transcription [23]. Furthermore, RNA acylating reagents are highly tunable in terms of their solubility, steric bulk, aqueous half‐life and selectivity, and have the propensity to react at multiple sites along a single RNA strand with minimal structure or sequence specificity [12]. To date, Acyl‐RNA models have been shown to exhibit unique biological properties for effective structural mapping [24], stabilization [10], enrichment and separation [25], intracellular delivery and imaging [26, 27].

Equally, the reactivation of functional RNA has been widely documented through small‐molecule deprotection reactions utilizing DABCO [10], Tris [10], histamine [14], *N*‐methylimidazole [14], tetrazine [11, 28], and hydrogen peroxide [29, 30] to selectively reinstate the 2’‐OH unit in multiple RNA models. Research groups have also investigated phosphine‐based mechanisms to deprotect 2’‐azide conjugates via Staudinger reduction reactions [8, 11, 31, 32, 33, 34]. More recently, Pd‐catalyzed reductive amination and demethylation has been reported as a route toward simultaneous 2’‐OH deprotection and purification of RNA [35, 36]. Non‐chemical strategies have also been reported, utilizing UV‐light exposure or endogenous esterases for the respective photo‐reversible or enzymatic restoration of native RNA structure [37, 38, 39]. Despite these advancements, 2’‐OH deprotection remains challenging in live cells, as treatment with chemical agents or UV light can give rise to side reactions causing loss of RNA activity and cytotoxicity [11, 13, 14]. Hence, designing alternative 2’‐modification strategies that allow for efficient and mild deprotection in a cellular environment would lead to the release of native, intact RNA for wider therapeutic use.

Disulfide‐based prodrug chemistry has been highlighted as an attractive route toward the non‐toxic removal of acyl conjugates from RNA. Acyl‐imidazole reagents reported previously by the Zhu and Zhou research groups were observed to react at the 2’‐OH position in high yields, disrupting the correct folding, hybridization and catalytic activity of various RNA models [26, 40, 41]. Moreover, these disulfide‐based conjugates were reported to have high sensitivity to reducing agents such as dithiothreitol (DTT) and reduced glutathione (GSH) both in vitro and under cytosolic conditions. Removal of such groups within a reducing environment was effective in manipulating the activity of a short‐interfering RNA (siRNA), a short‐guide RNA (sgRNA) and a longer mRNA model.

**FIGURE 1:**
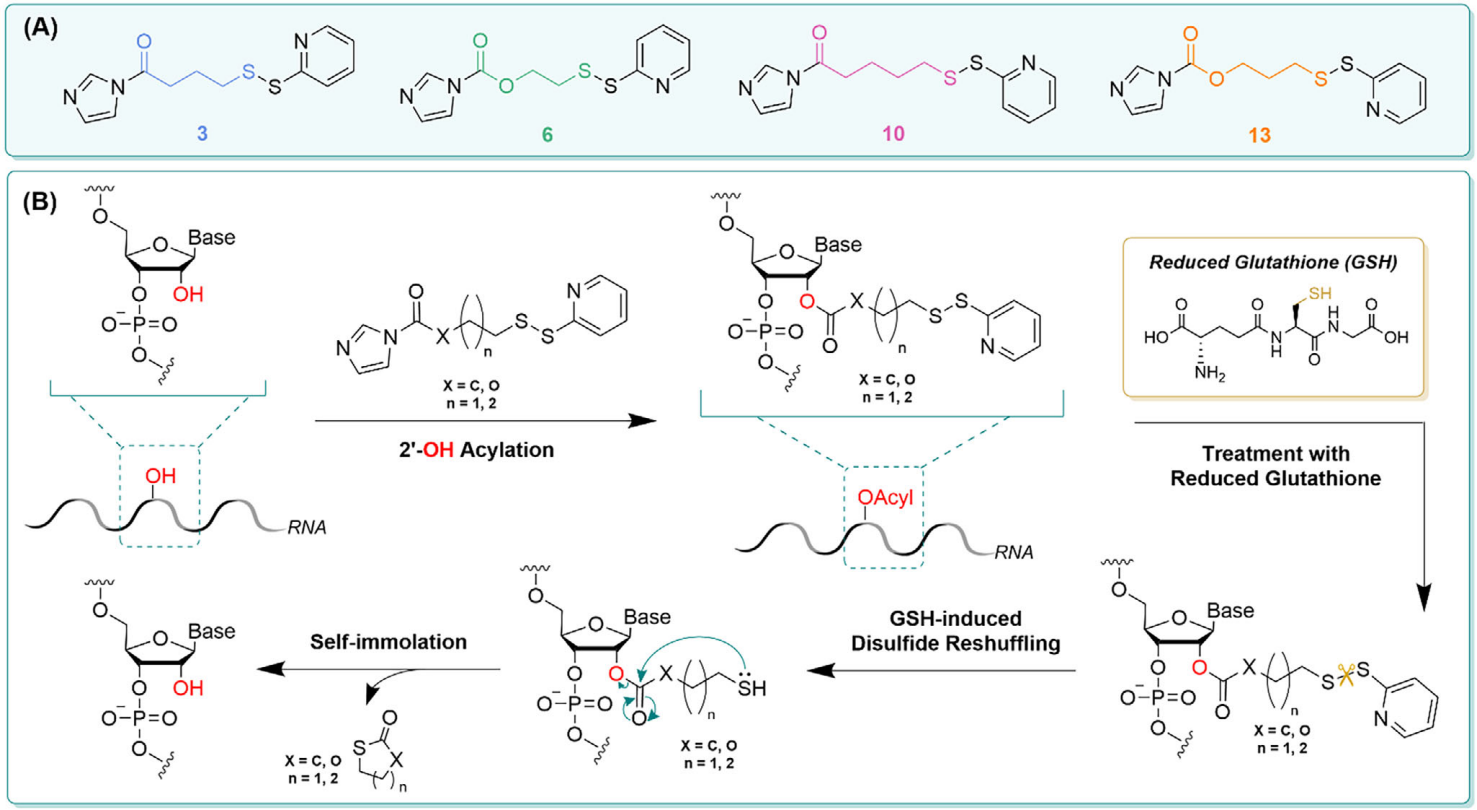
(A) Structural diversity of acylating reagents included in this study, with disulfide‐based self‐immolative linker moieties highlighted. (B) Post‐synthetic 2’‐OH acylation of RNA to produce Acyl‐RNA strands, and subsequent treatment with reduced glutathione to induce disulfide reshuffling, self‐immolation of 2’‐conjugates, and recovery of functional RNA.

In this study, we set out to expand the current library of disulfide‐based acyl‐imidazole reagents and examine the effects of structural modification on the activation of RNA. Two parameters within our investigation included extended alkyl chain length and the formation of ester or carbonate conjugates at the 2’‐position (Figure 1A). By altering the structure of these reagents, we report significant variation in acylation efficiency between Acyl‐RNA models. This strategy also serves as a basis for the development of a tunable release system for Acyl‐RNA conjugates containing a self‐immolative linker (Figure 1B). Based on the unique structural and electronic properties of the specified 2’‐modification, it is possible to control the self‐immolation and deprotective release of native RNA over time. From a therapeutic perspective, we further demonstrate the ability of these 2’‐modifications to hinder the enzymatic digestion and ribosomal processing of RNA, resulting in improved cellular stability and translation efficiency. This work describes a biocompatible approach for the temporal control of RNA structure and function, opening up a new field of targeted vaccine and prodrug technologies with extended therapeutic half‐lives.

## 2 Results and Discussions

The design of our acylating reagents included an electrophilic acyl‐imidazole site for reactivity with the 2’‐OH position, as well as a disulfide bond, separated by an alkyl spacer of two, three, or four carbon atoms. It was envisioned that alteration of the self‐immolative linker moiety would induce RNA release at varying rates when exposed to an overall reducing environment. This hypothesis stems from the observed speed of five‐membered ring formations over six‐membered rings. Six‐exo‐trig cyclizations have been shown to occur at slower rates than five‐exo‐trig cyclizations in accordance with Baldwin's rules [42, 43, 44]. Moreover, the insertion of an electronegative oxygen atom at the alpha position of the carbonyl would alter the stereoelectronic effects of ring‐closure further. With this variation, our series of disulfide‐based self‐immolative 2’‐modifications were anticipated to exhibit differing kinetic profiles during reductive RNA deprotection. In addition, 2’‐mercaptopyridine was chosen as the protecting group at the distal side of the self‐immolative linker, as it was predicted that its planar, aromatic structure would minimize steric hinderance for 2’‐conjugation at less accessible ribose sites along an RNA strand [41].

**SCHEME 1:**
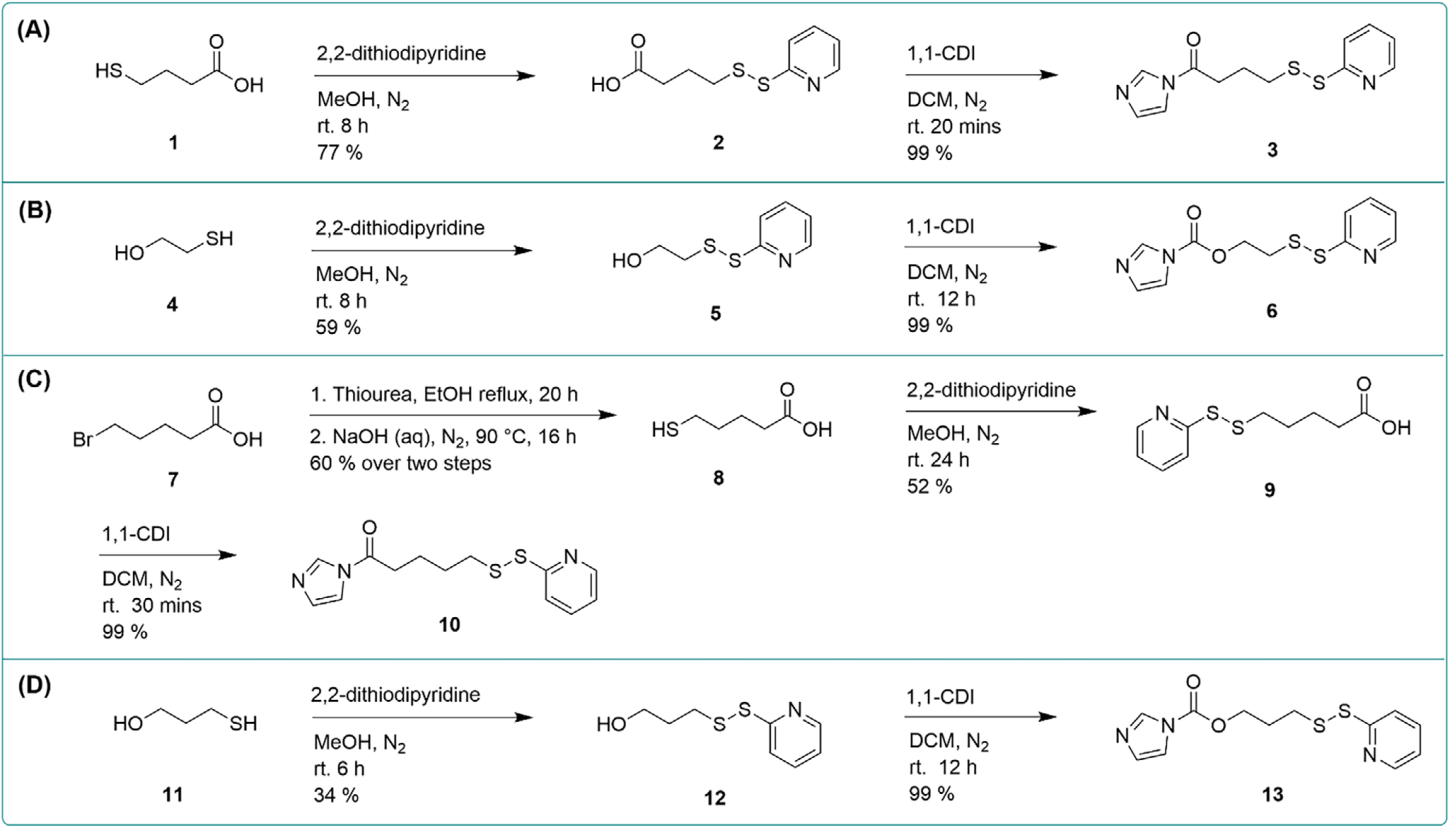
Synthesis of acyl‐imidazole and imidazole‐carbamate RNA 2’‐OH acylating reagents **3**, **6**, **10**, and **13**.

Synthesis of carboxylic acid **2** was achieved in 77% yield by reacting 4‐mercaptobuytric acid with 2,2‐dithiodipyridine under inert conditions (Scheme 1A). Disulfide **5** was obtained in 59% yield through the reaction of 2,2‐dithiodipyridine with primary thiol 2‐mercaptoethanol (Scheme 1B). 5‐Mercaptopentanoic acid **8** was synthesized in 60% yield through an initial reflux of 5‐bromopentanoic acid in the presence of thiourea, followed by an acidic workup. Subsequently, carboxylic acid **8** was reacted with 2,2‐dithiodipyridine under inert conditions to provide disulfide **9** in 52% yield (Scheme 1C). Finally, disulfide **12** was synthesized in 34% yield through the reaction of 2,2‐dithiodipyridine with corresponding primary thiol 3‐mercaptopropanol (Scheme 1D). Following formation of the disulfide moiety, a one‐step activation of **2**, **5**, **9**, and **12** with 1,1‐carbonyldiimidazole under inert conditions afforded acylating reagents **3**, **6**, **10**, and **13**, respectively, in quantitative yields.

**FIGURE 2:**
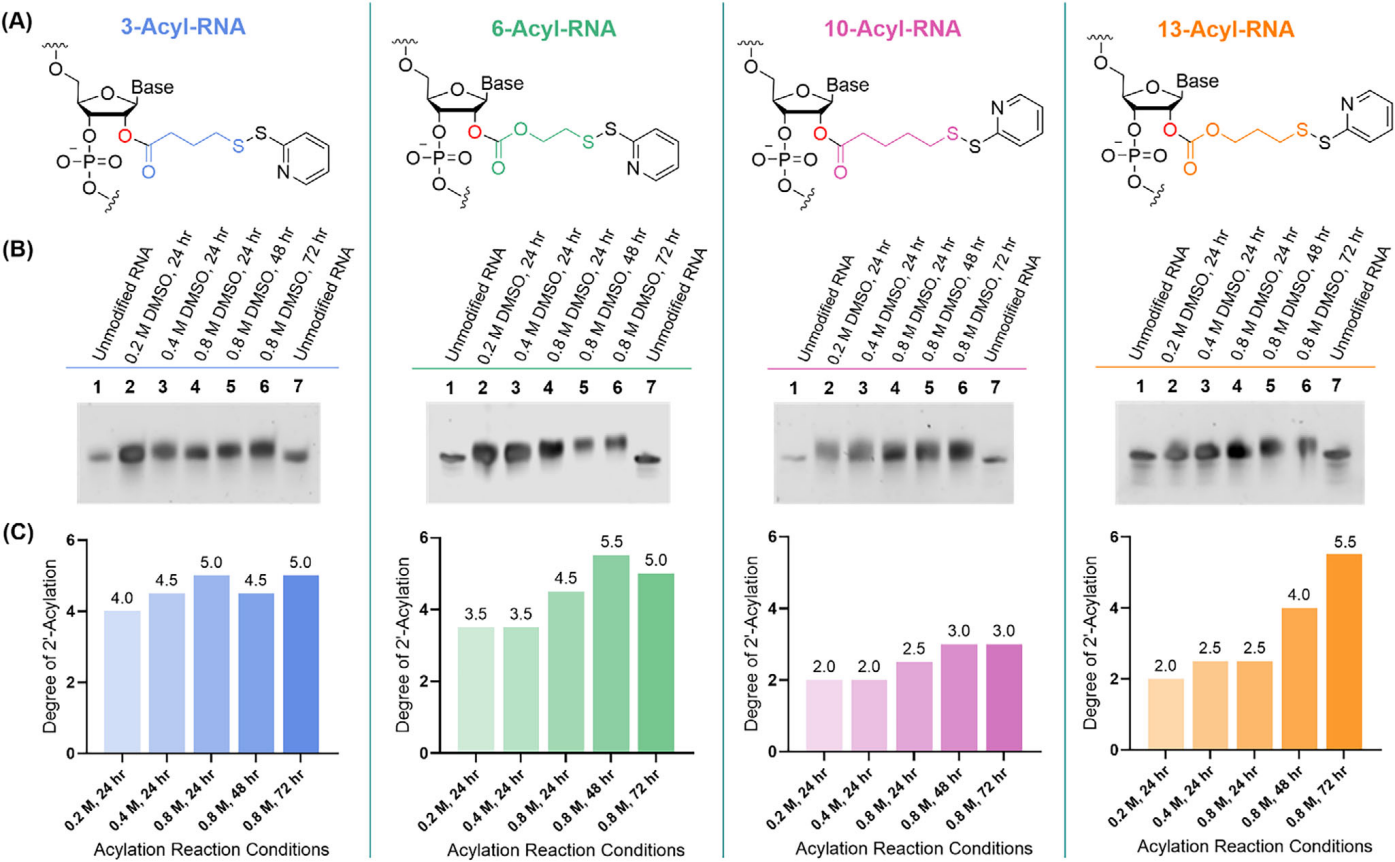
(A) Structural diversity of modified Acyl‐RNA strands included in this study, with disulfide‐based self‐immolative linker moieties highlighted. (B) Urea‐PAGE analysis of Acyl‐RNA strands under various reaction conditions for 2’‐acylation, including increasing acylating reagent DMSO stock concentration and reaction duration. (C) Degree of 2’‐acylation achieved for each reaction condition, quantified using MALDI‐ToF mass spectrometry as the median number of acyl‐conjugates per Acyl‐RNA strand.

To produce Acyl‐RNA strands using reagents **3**, **6**, **10**, or **13**, we first optimized the reaction conditions for 2’‐acylation utilizing a short, single stranded 18‐nucleotide RNA model (**RNA1**). Acylating reagents were added at final concentrations of 0.8, 0.4, and 0.2 M to a 50 µM solution of **RNA1** and incubated at room temperature for up to 72 h (Figure 2A). After this time, RNA was purified by ethanol precipitation. Urea‐PAGE analysis of the Acyl‐RNA products indicated that all reagents, most notably imidazole‐carbamates **6** and **13**, produced RNA strands with altered electrophoretic mobility (Figure 2B, Lanes 2–6). Moreover, the extent of acylation of available 2’‐OH positions appeared to increase with reaction duration as well as with increasing concentration of acylating reagent. The degree of acylation was further investigated using MALDI‐ToF mass spectrometry, showing a median number of up to six acyl groups per RNA strand (Figures 2C and S1A–E). Both intact disulfide and reduced thiol *m*/*z* values were present within the recorded MALDI‐ToF spectra for Acyl‐RNA samples, most likely arising from the high laser power used during analysis that can cleave disulfide bonds through spontaneous in‐source decay mechanisms [45]. We subsequently demonstrated the regioselectivity of acylating reagents **3**, **6**, **10**, and **13** for the 2’‐OH position of the RNA ribose unit, by reacting with an analogous 18‐nucleotide DNA sequence (**DNA1**) as well as a 2’‐OMethyl‐derived RNA (**RNA2**). From the resulting MALDI‐ToF spectra, +1 disulfide and reduced thiol conjugates were observed for **DNA1** samples incubated with **3**, **6**, and **10**, respectively, likely through acylation at exocyclic amines on nucleobases or the 3’‐ and 5’‐terminal hydroxy groups (Figure S2A) [8, 14, 38]. In contrast, only reagents **6** and **13** produced +1 conjugates when incubated with **RNA2**, which was further visualized via urea‐PAGE as a shift in the native electrophoretic band (Figure S2B). The increased selectivity of reagents **3** and **10** for the 2’‐OH position of RNA has added benefits in reducing off‐target reactivity when considering more biologically relevant models such as mRNA. It has been reported previously that acyl‐modifications at the exocyclic amines of cytidine residues are well‐tolerated intracellularly, and impact mRNA activity in a location‐dependent manner, showcasing an increase in biological half‐life and stability [46, 47, 48], reduced cytokine secretion during immune stimulation [49], and the facilitated localization of RNA‐binding proteins to stress granules [47]. When considering potential effects on translation efficiency, acyl‐nucleobase modifications along the 5’‐UTR region have been shown to competitively inhibit translation initiation, whereas inclusion along mRNA wobbles sites had a profound promotion of translation both in vitro and in vivo [48]. In addition, acyl‐modifications at the terminal hydroxy units of RNA have been shown to confer complete resistance of the oligonucleotide to enzymatic digestion by phosphodiesterases [50, 51]. To reduce the likelihood of this reactivity from occurring, biologically derived RNAs are often phosphorylated at both terminal positions, in contrast to synthetic RNA models. We anticipate therefore that the 2’‐OH selectivity of **3**, **6**, **10**, and **13** for RNA will likely depend on a number of factors such as the sequence, length, and codon composition of each target biomolecule [49].

**FIGURE 3:**
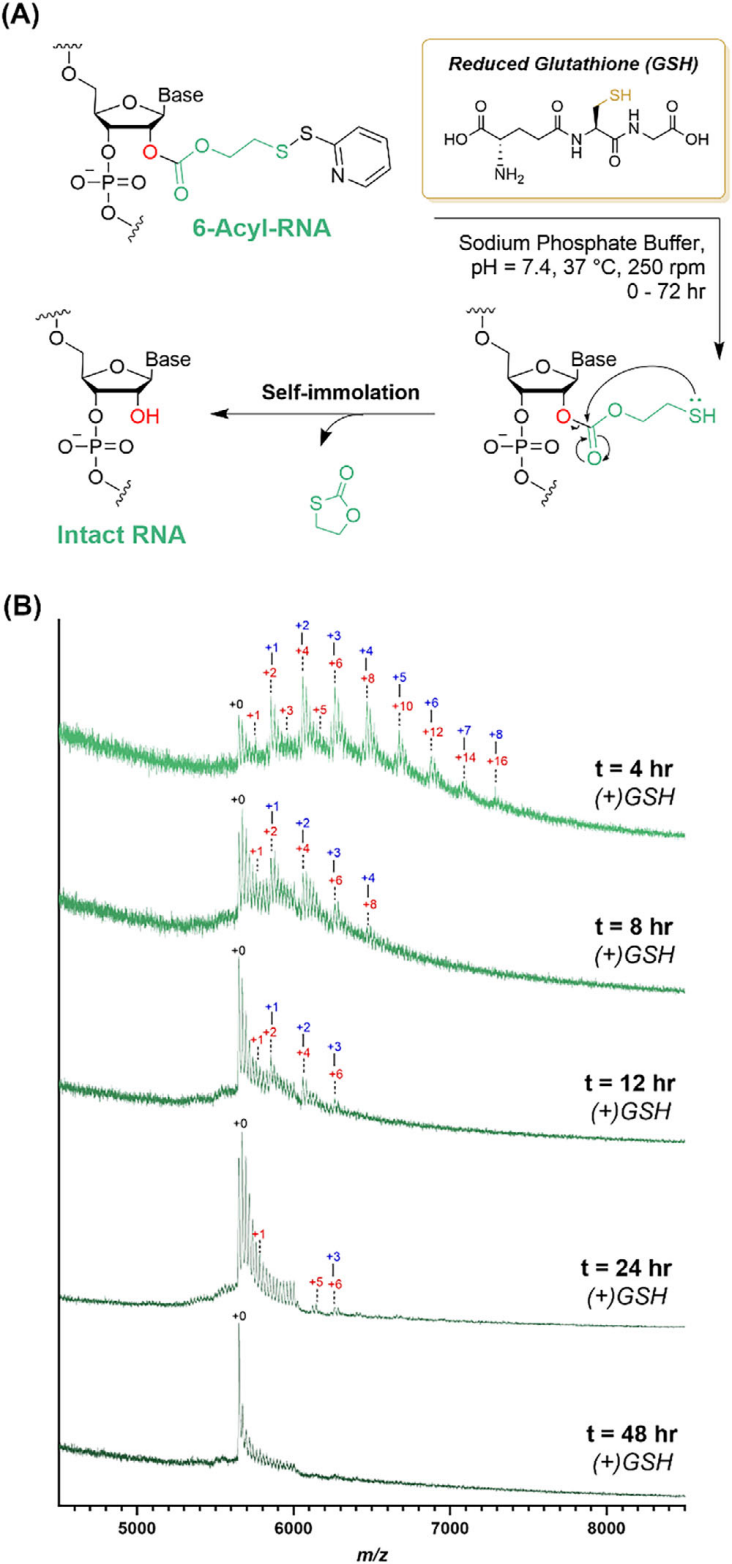
(A) Schematic representation of the self‐immolation and removal of 2’‐conjugates from **6**‐Acyl‐RNA via reaction with 10 mM reduced glutathione in sodium phosphate buffer. (B) MALDI‐ToF *m*/*z* spectra of **6**‐Acyl‐RNA samples incubated in the presence of 10 mM reduced glutathione at various time points. The number of intact disulfide conjugates (+X) are denoted in blue alongside the number of reduced thiol conjugates (+Y), denoted in red.

Having successfully demonstrated RNA reactivity to produce 2’‐acylated models, the disulfide cleavage and self‐immolation of **3**‐Acyl‐RNA, **6**‐Acyl‐RNA, **10**‐Acyl‐RNA and **13**‐Acyl‐RNA to reinstate the 2’‐alcohol was monitored in a cytosol‐mimetic environment of 10 mM reduced glutathione at 37°C. Analysis via urea‐PAGE showed that RNA strands protected with acylating reagents **3**, **10**, or **13** remained partially deprotected over a 72‐h period (Figure S3A–C). The incomplete removal of acyl‐conjugates was subsequently investigated using MALDI‐ToF mass spectrometry, with **3**‐Acyl‐RNA, **10**‐Acyl‐RNA, and **13**‐Acyl‐RNA still exhibiting median values of up to +6, +6, and +3 reduced thiol signals respectively after 72 h in the presence of GSH (Figure S4A–D). In contrast, **6**‐Acyl‐RNA underwent complete removal of all 2’‐acyl groups after approximately 48 h, leading to full recovery of native RNA. This result shows the promise of reagent **6** for the reversible modification of RNA in reducing cellular environments, but also highlights the importance of small changes in the structure of the acylating reagents (Figures 3A,B and S3D). The rapid elimination of **6‐**Acyl‐RNA in comparison to **3‐**Acyl‐RNA further supported previous reports outlining a faster rate of deprotection for carbonate‐based 2’‐conjugates when compared with 2’‐ester derivatives [41]. This comparison was further quantified based on the relative rates of **RNA1** recovery in the presence of 10 mM reduced glutathione at 37°C, with **6**‐Acyl‐RNA demonstrating the fastest recovery of native RNA and showing up to a 3.5× fold difference between linker architectures (Figure S4E). It was hypothesized that for **3**‐Acyl‐RNA, the absence of intact disulfide *m*/*z* values in the MALDI spectra was due to a rapid, GSH‐induced cleavage of the disulfide and loss of the 2‐mercaptopyridine moiety, followed by a rate‐limiting self‐immolation step. In addition, RNA conjugated by **10** and **13** underwent incomplete deprotection during this period, corresponding to the anticipated slower kinetics of 6‐membered ring closures in comparison to 5‐membered rings [42, 43, 44]. Interestingly, the formation of multiple, stable reduced glutathione 2’‐conjugates were evident within the MALDI‐ToF spectra of **13**‐Acyl‐RNA, likely forming as a result of rapid reduction of the disulfide **13**, as well as a spontaneous oxidation of GSH to form a labile glutathione disulfide (GSSG) (Figure S4D) [52]. Urea‐PAGE analysis further supported the presence of multiple RNA‐GSH species as multiple bands at increasing molecular size (Figure S3C).

The aqueous stability of Acyl‐RNA strands was also evaluated in phosphate buffer in the absence of GSH over a 72‐h period. In this case, no hydrolysis of the 2’‐esters was observed (Figure S5). This confirmed that a shift in *m*/*z* values and the emergence of the native, deprotected RNA peak was due to the presence of GSH in solution.

**FIGURE 4:**
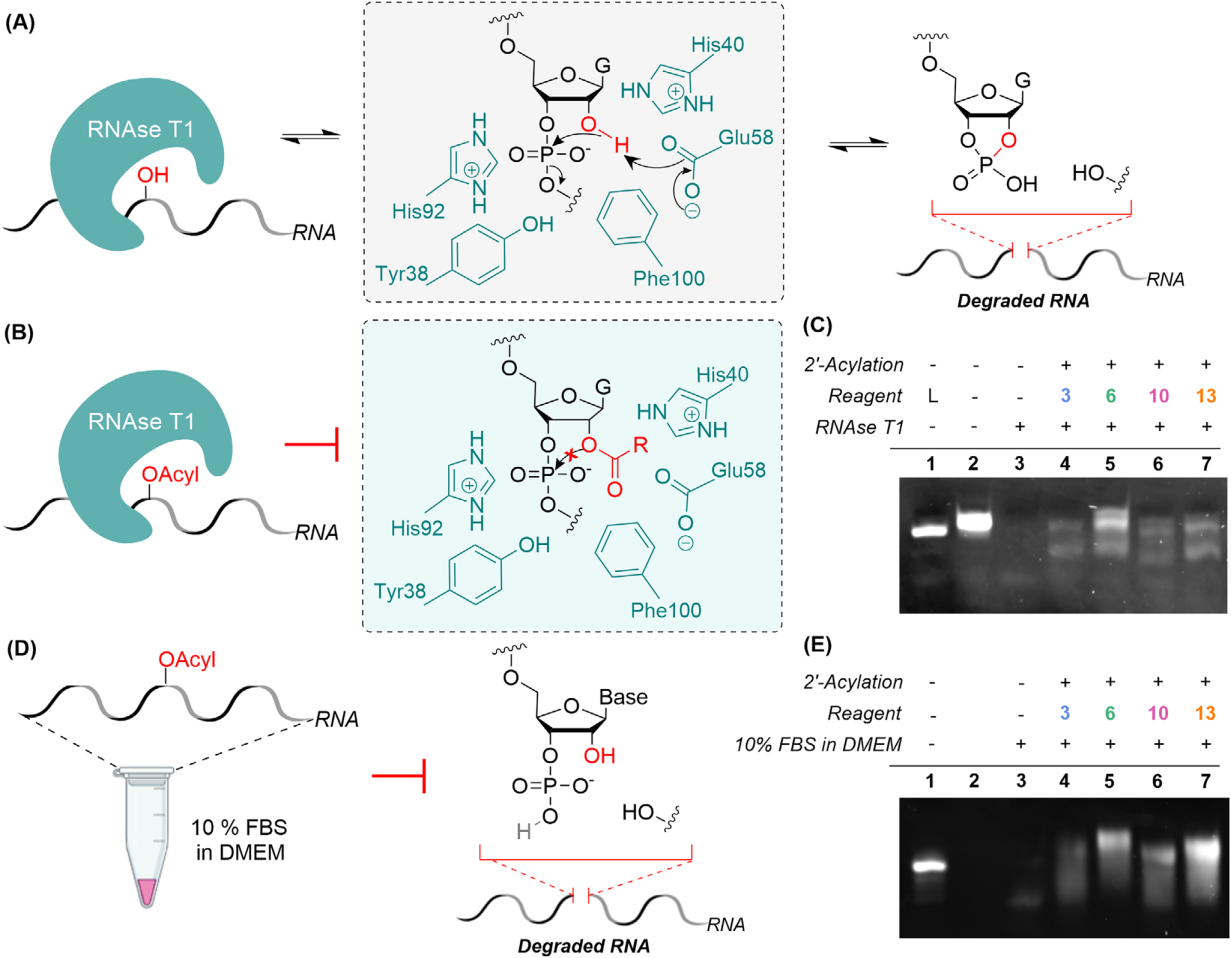
(A) RNase T1‐mediated digestion of an unmodified, single‐stranded RNA at the 3’‐end of guanosine residues. Amino acid side chains within the active site facilitate deprotonation of the ribose 2’‐OH unit resulting in intramolecular nucleophilic attack on the adjacent phosphodiester and degradation of the RNA strand [53]. (B) Reduced RNase T1 recognition of a 2’‐acylated single‐stranded RNA at the 3’‐end of guanosine residues. Acylation of the 2’‐OH unit inhibits RNA–enzyme interactions within the active site, preserving the biomolecule from degradation [10]. (C) Evaluation of RNase T1‐mediated digestion of unmodified RNA and **3**‐, **6**‐, **10**‐, and **13**‐Acyl‐RNA samples via urea‐PAGE (L = RNA ladder consisting of 15, 9, and 6 nt strands). (D) Reduced nuclease digestion of **3**‐, **6**‐, **10**‐, and **13**‐Acyl‐RNA samples in the presence of Dulbecco's modified Eagle medium (DMEM) supplemented with 10% fetal bovine serum (FBS). (E) Evaluation of serum‐mediated digestion of unmodified RNA and **3**‐, **6**‐, **10**‐, and **13**‐Acyl‐RNA samples via urea‐PAGE.

Motivated by these results, we investigated the effects of 2’‐OH conjugation on enzymatic degradation, as this would further indicate the stability of Acyl‐RNA models within a cellular environment. We first utilized RNase T1, an endoribonuclease that catalyzes the degradation of single‐stranded RNA at guanosine residues as our model enzyme, as it has been postulated that the Glu58 residue within its active site facilitates the base‐catalyzed deprotonation of the 2’‐OH unit and subsequent intramolecular attack on the neighboring phosphodiester (Figure 4A) [53, 54]. By treating an unmodified RNA control with the enzyme, we observed significant degradation by urea‐PAGE analysis (Figure 4C, Lane 3). RNA samples that were acylated by **3**, **6**, **10**, or **13** showed a lower extent of enzymatic digestion relative to the unmodified RNA control, indicating that 2’‐acylation was efficient in preventing enzymatic cleavage of the RNA strand (Figure 4B,C, Lanes 3–7) [10]. To further investigate this increased stability under biologically relevant contexts, we assessed the nuclease resistance of unmodified RNA and RNA samples acylated by **3**, **6**, **10**, or **13** in the presence of Dulbecco's modified Eagle medium (DMEM) supplemented with 10% fetal bovine serum to replicate a eukaryotic cell culture environment (Figure 4D). To our excitement, we consequently observed a lower extent of RNA digestion by urea‐PAGE analysis when **3**‐Acyl‐RNA, **6**‐Acyl‐RNA, **10**‐Acyl‐RNA, and **13**‐Acyl‐RNA samples were treated with serum and compared with an unmodified RNA control (Figure 4E, Lanes 3–7). To further reinforce our hypothesis that reagents **3**, **6**, **10**, or **13** were effective in enhancing the nuclease resistance of single‐stranded RNA, we utilized RNase A as an additional model enzyme, as its related mechanism of RNA digestion relies on the reactivity of the His12 and His119 active site residues to catalyze deprotonation of the 2’‐OH unit of RNA at the 3’‐end of pyrimidine nucleotides (Figure S6A) [55]. Through urea‐PAGE analysis of RNase A‐treated samples, we were successful in further demonstrating the increased resistance of Acyl‐RNA models to endoribonuclease‐mediated digestion when compared to an unmodified RNA control (Figure S6B,C, Lanes 3–7). Interestingly, **6**‐Acyl‐RNA showed the greatest increase in nuclease resistance under all simulated conditions, further reinforcing the potential applicability of this particular modification in stabilizing RNA‐based formulations for long‐term storage, sustained intracellular release and prolonged therapeutic activity.

To further examine the potential use of **3**, **6**, **10** and **13** in the stabilization of RNA therapeutics and vaccines, we shifted our focus toward longer, intrinsically more unstable biological models such as mRNA. In vitro transcription was used to produce mRNA molecules encoding for green‐fluorescent protein (GFP, 730 nt), as well as nanoluciferase (NanoLuc, 519 nt). We carried out 2’‐acylation experiments under similar conditions as previously, reacting solutions of **3**, **6**, **10**, or **13** with mRNA to produce Acyl‐GFP‐mRNA and Acyl‐NanoLuc‐mRNA models with high electrophoretic integrity and minimal observed degradation (Figure S7). Initially, unmodified GFP mRNA and Acyl‐GFP‐mRNA samples were transfected into human embryonic kidney 293 (HEK293) cells using Lipofectamine 2000 to investigate potential cellular toxicity arising from 2’‐conjugates or by‐products during self‐immolation. An MTT assay was performed at various timepoints post‐transfection (24, 72, 144 h) to quantify changes in percentage cell viability. When normalized to untreated control cells, there were negligible differences between non‐acylated GFP mRNA and Acyl‐GFP‐mRNA models at each transfection timepoint (Figure S8).

**FIGURE 5:**
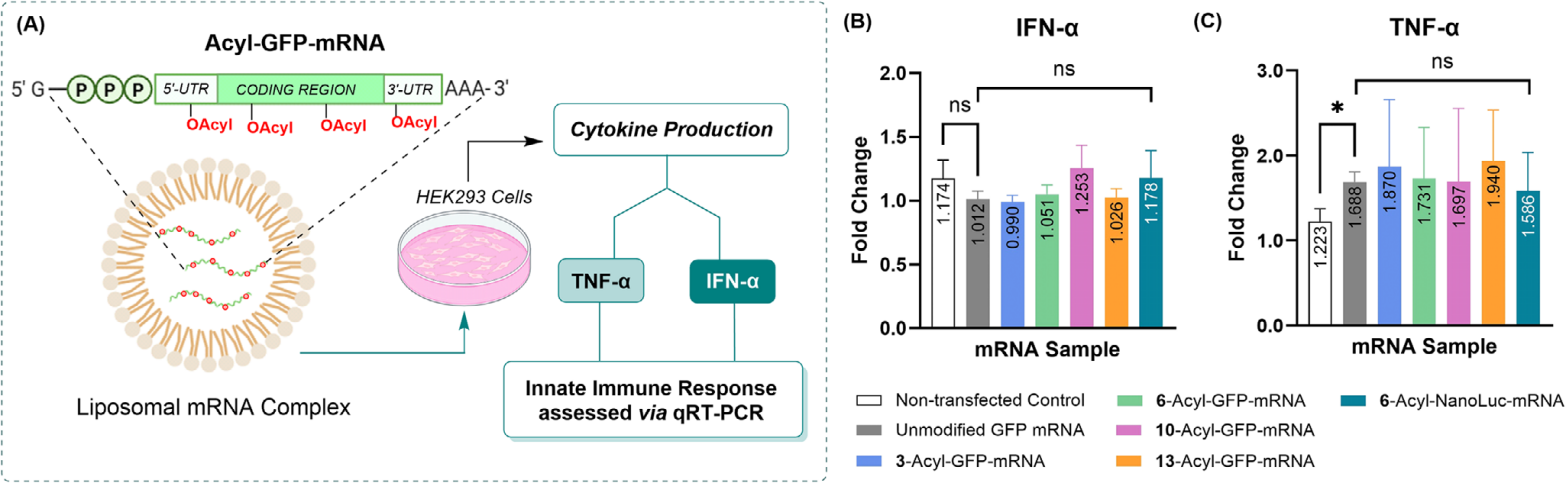
(A) Schematic representation of the liposomal entrapment and transfection of Acyl‐GFP‐mRNA models conjugated to **3**, **6**, **10**, and **13**, respectively, into HEK293 cells, producing cytokine responses for interferon alpha (IFN‐α) and tumor necrosis factor alpha (TNF‐α) measured via qRT‐PCR. Graphic created in BioRender (https://www.biorender.com). (B) Fold change values obtained from qRT‐PCR analysis for IFN‐α expression levels in non‐transfected cells and cells treated with unmodified GFP mRNA and Acyl‐GFP‐mRNA constructs (mean ± SD; *n* = 3 independent experiments). (C) Fold change values obtained from qRT‐PCR analysis for TNF‐α expression levels in non‐transfected cells and cells treated with unmodified GFP mRNA and Acyl‐GFP‐mRNA constructs (mean ± SD; *n* = 3 independent experiments). Statistical significance was calculated using unpaired two‐tailed Student's *t*‐test and one‐way ANOVA followed by Tukey's post‐hoc test (ns = non‐significant, *p ≤ 0.05).

To better understand the promising biological compatibility of reagents **3**, **6**, **10**, and **13** as 2’‐conjugates of mRNA, we subsequently looked toward assessing innate immune activation upon transfection of unmodified and modified constructs. Cells were lysed 24 h post transfection, and total cellular RNA was isolated for quantitative reverse transcription polymerase chain reaction (qRT‐PCR) analysis (Figure 5A). We first quantified levels of interferon alpha (INF‐α), an important Type‐1 interferon produced by cells following the innate immune response to cellular stressors such as viral nucleic acids or cancer [56]. After 24 h, IFN‐α gene expression remained unchanged across all treatment groups, indicating minimal induction of innate immune activation by Acyl‐GFP‐mRNA models (Figure 5B). As an additional cytokine model, we examined the effects of mRNA 2’‐acylation on tumor necrosis factor alpha (TNF‐α) production as an indicator of cellular inflammation and apoptosis [57]. When normalized to untreated controls, cells transfected with unmodified GFP mRNA induced a significant increase in TNF‐α expression, whereas no significant changes were observed for Acyl‐GFP‐mRNA constructs compared with the unmodified GFP mRNA control (Figure 5C). Together, these results highlight the potential application of reagents **3**, **6**, **10**, and **13** in the development of Acyl‐mRNA vaccine candidates for the treatment of cancer and infectious diseases.

**FIGURE 6:**
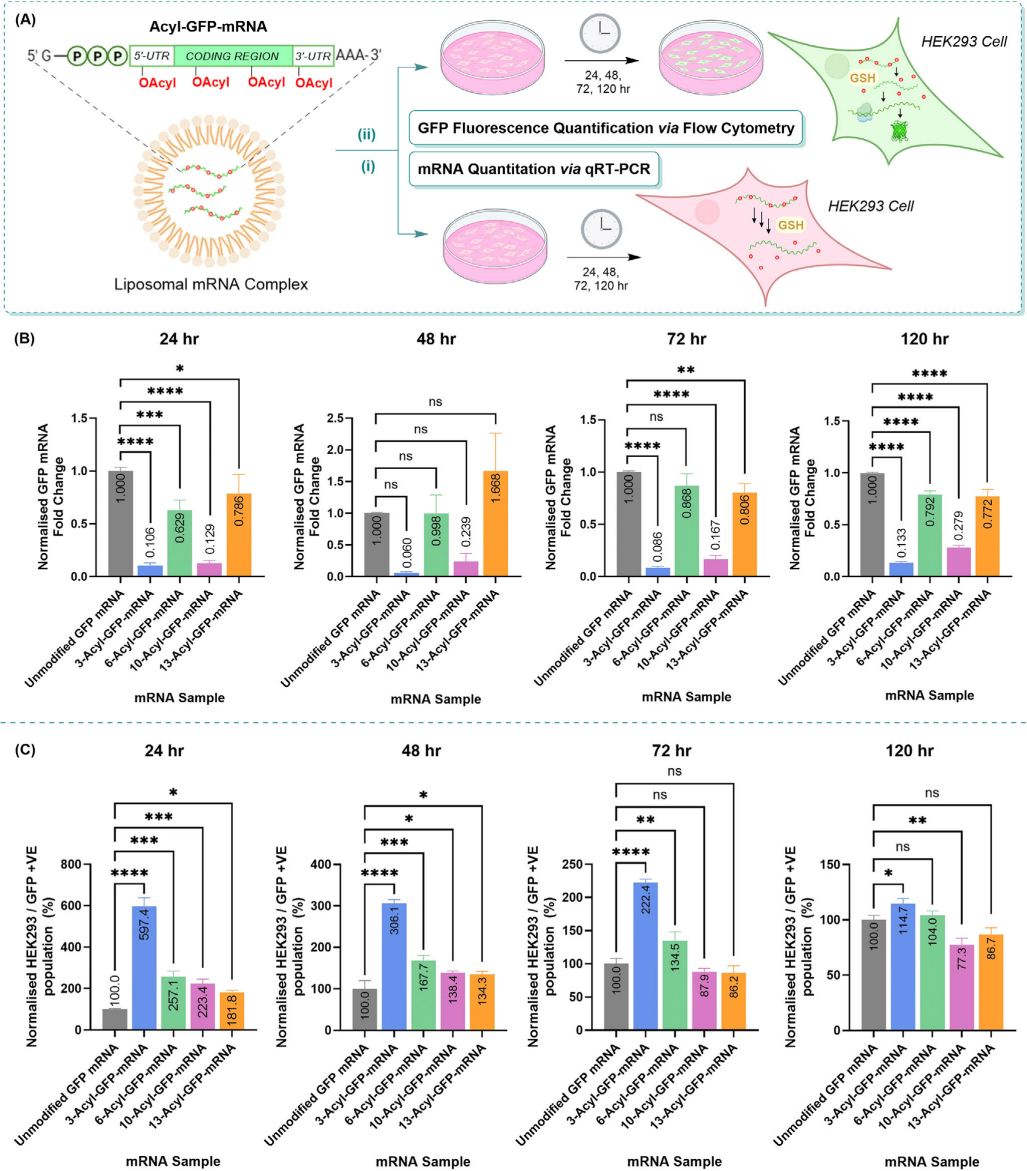
(A) Schematic representation of the liposomal entrapment and transfection of Acyl‐GFP‐mRNA models conjugated to **3**, **6**, **10**, and **13**, respectively, into HEK293 cells. (i) qRT‐PCR analysis of Acyl‐GFP‐mRNA samples 24, 48, 72, and 120 h post‐transfection. (ii) Flow cytometry analysis of GFP fluorescent signal 24, 48, 72, and 120 h post‐transfection. Graphic created in BioRender (https://www.biorender.com). (B) Fold change values obtained from qRT‐PCR analysis for Acyl‐GFP‐mRNA samples conjugated to **3**, **6**, **10**, and **13**, respectively, normalized to an unmodified GFP mRNA control at each transfection timepoint (mean ± SD; *n* = 3 independent experiments). (C) Population percentages of HEK293 cells expressing GFP signal obtained from flow cytometry analysis for Acyl‐GFP‐mRNA samples conjugated to **3**, **6**, **10**, and **13**, respectively, normalized to an unmodified GFP mRNA control at each transfection timepoint (mean ± SD; *n* = 3 independent experiments). Statistical significance was calculated using one‐way ANOVA followed by Tukey's post‐hoc test (ns = non‐significant, *p ≤ 0.05, **p ≤ 0.01, ***p ≤ 0.001, and ****p ≤ 0.0001).

Having shown positive tolerability responses toward our acylated‐mRNA models, we subsequently investigated the effects of 2’‐acylation on target GFP mRNA levels via qRT‐PCR. A time‐course experiment was carried out to isolate total cellular RNA from cells transfected with unmodified GFP mRNA and GFP mRNA 2’‐conjugated by **3**, **6**, **10**, and **13** at 24, 48, 72, and 120 h post‐transfection (Figure 6Ai). Cells transfected with **3**‐Acyl‐GFP‐mRNA and **10**‐Acyl‐GFP‐mRNA showed reduced mRNA amounts when compared to a control consisting of cells transfected with non‐acylated GFP mRNA. This is likely due to inefficient removal of these RNA modifications by GSH, as observed in our earlier in vitro experiments. In contrast, carbonate‐based derivatives **6**‐Acyl‐GFP‐mRNA and **13**‐Acyl‐GFP‐mRNA exhibited markedly higher mRNA levels relative to the control 48 h post‐transfection, which steadily decreased after 72 h. This dynamic variation in the observed mRNA levels presents a sustained, non‐toxic release of stable, native GFP mRNA within the cytosol, highlighting the strong therapeutic applicability of these modifications in the activation of RNA‐based prodrugs (Figure 6B).

To examine this exciting result further, we monitored the extent of GFP protein production in HEK293 cells transfected with Acyl‐GFP‐mRNA samples modified with **3**, **6**, **10**, or **13**, respectively, across various timepoints post‐transfection (Figure 6Aii). It has been previously reported that 2’‐acylation along the mRNA coding region can significantly attenuate or prevent the translation of mRNA both in vitro and in cultured cells [10, 11, 41]. However, we hypothesized that the self‐immolation of our disulfide‐based modifications would gradually release translatable mRNA, potentially leading to an overall increase in total protein production relative to an unmodified mRNA control. To carry out this time‐course experiment, GFP fluorescence was quantified in HEK293 cells for all 2’‐acylated mRNA samples and subsequently normalized to a non‐acylated mRNA control at each time point (Figure 6C). Based on previous qRT‐PCR results for cells treated with ester‐based **3**‐Acyl‐GFP‐mRNA and **10**‐Acyl‐GFP‐mRNA samples, a stark reduction in GFP fluorescence signal was expected due to the inefficient removal of acyl modifications. Surprisingly, however, a significant increase in normalized GFP fluorescent signal was observed across both carbonate‐ and ester‐based GFP mRNA samples, particularly at 24 and 48 h post‐transfection. Most notably, cells treated **3**‐Acyl‐GFP‐mRNA exhibited a ∼600% increase in GFP fluorescent signal after 24 h, indicating a rapid and continuous release of mRNA within this initial period. Significantly, cells treated with **3**‐Acyl‐GFP‐mRNA and **6**‐Acyl‐GFP‐mRNA samples achieved a markedly higher level of protein expression compared to the unmodified control for all recorded time points. This sustained increase was not observed in cells transfected with **10**‐Acyl‐GFP‐mRNA and **13**‐Acyl‐GFP‐mRNA. The expected faster kinetics of five‐membered ring formations over six‐membered ring formations during self‐immolation of the acyl modifications may help to rationalize this increase. In addition, it was found that the decrease in normalized GFP signal over time was proportional to the self‐immolation rates of **3**‐Acyl‐GFP‐mRNA, **6**‐Acyl‐GFP‐mRNA, **10**‐Acyl‐GFP‐mRNA, and 1**3**‐Acyl‐GFP‐mRNA in forming five‐ and six‐membered rings (Figure S10). This result further reinstated that a tuning of mRNA deprotection kinetics leads to a clear difference in prolonging its biological effect. After 120 h, a notable recovery of protein expression was evident in almost all 2’‐conjugated samples compared to the native control, supporting previous work surrounding the effects of 2’‐OH acylation in proactively downregulating and activating mRNA translation [10, 11, 41].

Following the qRT‐PCR and flow cytometry results, which provide insight into the release kinetics of carbonates **6** and **13**, we decided to confirm the broad applicability of our mRNA prodrug approach by producing an alternative target protein, Nano‐Luc. Utilizing Acyl‐NanoLuc‐mRNA models modified by **6** or **13**, we transfected HEK293 cells via a Lipofectamine 2000 delivery system. A Nano‐Glo Luciferase Assay System was performed to measure the luminescence of samples at various timepoints post‐transfection (24, 48, 72, 144, 192 h) (Figure S11). This assay confirmed a negligible change in overall protein expression for **13**‐Acyl‐NanoLuc‐mRNA samples compared to the unmodified mRNA control. However, 2’‐conjugated **6**‐Acyl‐NanoLuc‐mRNA samples demonstrated a statistically significant increase in protein expression after 72 h compared to the non‐acylated control, emphasizing the generality of this modification in successfully prolonging and increasing the production of target protein.

From a mechanistic perspective, our overall findings suggest that 2’‐modification of mRNA with **3**, **6**, **10**, or **13** interferes with the interaction of translational machinery with the RNA, including initiation and elongation factors, tRNAs or the assembly or translocation of the 80S ribosome. Thus, by compromising elongation speeds and therefore protein synthesis rates, there is added potential to prolong the therapeutic half‐life of the modified mRNA via the action of endogenous glutathione in facilitating gradual disulfide cleavage and self‐immolation [10, 11]. When considering the degree of modification within a given sequence, we argue a partial degree of 2’‐acylation has benefits in providing increased translational control over a fully‐protected mRNA sequence, as endogenous intracellular levels of GSH are required to fully deprotect each mRNA strand within a relevant therapeutic window. Furthermore, we believe it is beneficial to attain a lower degree of modification to prevent degradation of the mRNA under 2’‐acylation reaction conditions, and to facilitate the efficient removal of 2’‐conjugates. Through this work, incomplete modification along a coding sequence or binding sequence has been shown to prolong target protein synthesis as well as reduce recognition by ribonucleases. Overall, this prodrug strategy serves as a basis toward the endogenous activation of stable, long‐acting and higher‐efficacy mRNA, opening up a new field of targeted cancer and infectious disease therapies.

## 3 Conclusion

In summary, by modifying the self‐immolative linker structure of acylating agents **3**, **6**, **10**, and **13**, a high degree of 2’‐acylation of RNA can be achieved under aqueous conditions. Exposure to physiological concentrations of glutathione resulted in either complete or partial recovery of intact, native RNA through removal of 2’‐acyl modifications in oligonucleotide model systems. Furthermore, our work has shown that modification at this position can considerably reduce RNA recognition and degradation via RNase T1, RNase A, and serum‐mediated digestion. This degree of nuclease resistance has additional benefits in improving the stability and facilitating the long‐term storage and handling of RNA [58]. The therapeutic applicability of these 2’‐modifications in mRNA was assessed using qRT‐PCR methods, highlighting the cellular stability and biocompatibility of acylated mRNA samples. Additional time‐course experiments revealed that within the reducing environment of the cell cytosol, changes in the release properties of 2’‐conjugates from mRNA led to significantly higher levels (up to ∼600%) of target protein production when compared to non‐acylated controls. This significant and sustained increase in protein production provides evidence of the temporal control of mRNA translation within the cell, and although we have not directly shown this is due to the modulated interaction of translational machinery with the RNA, we predict that a decoupling of deprotection kinetics could be elucidated in future work by monitoring cellular mRNA turnover at controlled in vitro GSH concentrations. Overall, this work exemplifies the idea of using endogenous stimuli to recover intact functional mRNA over a prolonged period, offering a gateway toward the development of targeted mRNA therapeutics for indications beyond current vaccine applications.

## Author Contributions

**Mary E. Flood**: conceptualization, writing – original draft, methodology, validation, visualization, data curation, investigation. **Sathishkumar Kurusamy**: investigation, writing – review and editing. **Mark Berney**: writing – review and editing, conceptualization. **C. Mark Smales**: methodology, writing – review and editing, resources, writing – original draft, funding acquisition. **Elizabeth M. Topp**: conceptualization, writing – review and editing, supervision, resources, funding acquisition, methodology. **Joanna F. Mcgouran**: conceptualization, methodology, writing – original draft, writing – review and editing, supervision. **Steven Ferguson**: conceptualization, funding acquisition, writing – original draft, writing – review and editing, supervision, resources, project administration.

## Conflicts of Interest

The authors declare no conflicts of interest.

## Supporting information



The authors have cited additional references within the Supporting Information [59, 60, 61, 62, 63].**Supporting File**: anie73630‐sup‐0001‐SuppMat.pdf.

## Data Availability

The data that supports the findings of this study are available in the Supporting Information of this article.
