# Supplementary material for: Glutathione‐Responsive Acyl‐Modifications for Targeted RNA Decaging and Prolonged Protein Synthesis
Source: Angew Chem Int Ed Engl. 2026 Jul 17;65(39):e8835235. doi: 10.1002/anie.8835235 (PMC13592295; doi:10.1002/anie.8835235)
Supplement: Supplementary file 1 [file ANIE-65-e8835235-s001.pdf]

Supporting Information

Glutathione-responsive Acyl-modifications for Targeted RNA Decaging and Prolonged Protein Synthesis

Mary E. Flood,[a,b] Sathishkumar Kurusamy,[c] Mark Berney,[d,b] C Mark Smales,[c,b] Elizabeth M. Topp,[e,b] Joanna F. McGouran*,[f] Steven Ferguson*[a,b]

[a] M. E. Flood, Prof. S. Ferguson*
RINN Pharma and Biopharma Centre, School of Chemical and Bioprocess Engineering, University College Dublin, Belfield, Dublin, Ireland
E-mail: steven.ferguson@ucd.ie
ORCID: https://orcid.org/0000-0002-8166-1956

[b] M. E. Flood, Dr. M. Berney, Prof. C. M. Smales, Prof. E. M. Topp, Prof. S. Ferguson
RINN Pharma and Biopharma Centre, National Institute for Bioprocess Research and Training, Foster Avenue, Mount Merrion, Dublin, Ireland

[c] Dr. S. Kurusamy, Prof. C. M. Smales
Industrial Biotechnology Centre, School of Natural Sciences, University of Kent, Canterbury, United Kingdom

[d] Dr. M. Berney
School of Chemistry and Chemical Engineering, Queen's University Belfast, Stranmillis Road, Belfast, United Kingdom

[e] Prof. E. M. Topp
Department of Industrial and Physical Pharmacy, Purdue University, West Lafayette, Indiana, United States

[f] Prof. J. F. McGouran*
RINN Pharma and Biopharma Centre, School of Chemistry and Trinity Biomedical Sciences Institute, Trinity College Dublin, The University of Dublin, Dublin, Ireland
ORCID: https://orcid.org/0000-0002-9349-2141

Table of Contents

General Experimental 3

Oligonucleotides sequences 3

GFP mRNA sequence 3

Nanoluciferase mRNA sequence 4

General RNA/DNA 2'-OH Acylation 4

General RNA 2'-Deprotection 4

General mRNA 2'-OH Acylation 5

MALDI-ToF Mass Spectrometry Analysis 5

Urea-PAGE 5

RNase T1 Gel Electrophoresis Assay 6

RNase A Gel Electrophoresis Assay 6

Fetal Bovine Serum Electrophoresis Assay 6

In Vitro Transcription 7

Automated Tapestation Workflow for RNA Integrity Analysis 7

Cell Culture & mRNA Transfection 7

MTT Assay 8

Luciferase Activity Assay 8

Flow Cytometry 8

Quantitative Reverse Transcription Polymerase Chain Reaction 8

Statistical Analysis 9

Table S1: qRT-PCR Primer Sequences 10

Synthesis of Acylating Reagents 11

Figure S1A: 17

Figure S1B: 18

Figure S1C: 20

Figure S1D: 23

Figure S1E: 25

Figure S2A: 27

Figure S2B: 29

Figure S3A: 31

Figure S3B: 31

Figure S3C: 32

Figure S3D: 32

Figure S4A: 33

Figure S4B: 36

Figure S4C: 38
Figure S4D: 41
Figure S4E: 44
Figure S5: 45
Figure S6: 46
Figure S7: 47
Figure S8: 48
Figure S9: 49
Figure S10: 50
Figure S11: 51
^{1}H & ^{13}C NMR Spectra and LC/MS (ESI+) Mass Spectra 52
References: 65

General Experimental

^{1}H and ^{13}C NMR spectra were recorded on a Varian VnmrS 400 MHz spectrometer, ^{1}H (400.13 MHz) and ^{13}C (100.6 MHz). Resonances δ, are in ppm units and referenced relative to residual $CHCl_3$ (δH = 7.26 ppm, δC = 77.0 ppm) or DMSO (δH= 2.50 ppm, δC= 39.52 ppm) as appropriate. Coupling constants are reported in Hertz (Hz). NMR spectra were assigned using HSQC, HMBC, and COSY experiments. Infrared (IR) spectra were measured using a Bruker ALPHA FT-IR spectrophotometer. High Resolution Mass Spectrometry (HRMS) was carried out using an Agilent 6546 Quadrupole Time-of-Flight (QToF) High Resolution Mass Spectrometer and associated Infinity Prime II 800 bar HPLC system with variable wavelength detector (VWD). Silica gel (particle size 60-200 µm, 60 Å, Aldrich) was used for flash column chromatography. Analytical thin-layer chromatography was performed on precoated Kieselgel 60F254 aluminium plates and visualized by UV irradiation (254 nm),or stained with potassium permanganate (3 g $KMnO_4$, 20 g K_2CO_3, 300 mL H_2O), vanillin (15 g vanillin, 250 mL EtOH, 2.5 mL conc. H_2SO_4), or p-anisaldehyde (15 ml AcOH, 3.5 mL of p-anisaldehyde, 350 mL EtOH, 50 mL conc. H_2SO_4). All solvents were obtained from commercial sources and used as received without further purification. Anhydrous solvents were used dry from SureSeal bottles or dried over 3 Å molecular sieves (Aldrich). Oxygen-free nitrogen was obtained from BOC gases and used without further drying. RNA and DNA oligonucleotides were obtained commercially from Eurogentec and used as received without further purification. RNA concentrations (ng/uL) were measured using a NanoDrop 2000 UV-Vis spectrophotometer (Thermo Fisher Scientific).

Oligonucleotides sequences

RNA1/2 (18 nt): 5’-AUC CUG CCG ACU ACG CCA-3’

DNA1 (18 nt): 5’-ATC CTG CCG ACT ACG CCA-3’

GFP mRNA sequence

AUGAGUAAAGGAGAAGAACUUUUCACUGGAGUUGUCCCAAUUCUUGUUGAAUUAGAUGGUGA
UGUUAAUGGGCACAAAUUUUCUGUCAGUGGAGAGGGUGAAGGUGAUGCAACAUACGGAAAAC
UUACCCUUAAAUUUAUUUGCACUACUGGAAAACUACCUGUUCCAUGGCCAACACUUGUCACUA
CUUUCUCUUAUGGUGUUCAAUGCUUUUCAAGAUACCCAGAUCAUAUGAAACAGCAUGACUUUU
UCAAGAGUGCCAUGCCCGAAGGUUAUGUACAGGAAAGAACUAUAUUUUUCAAAGAUGACGGG
AACUACAAGACACGUGCUGAAGUCAAGUUUGAAGGUGAUACCCUUGUUAAUAGAAUCGAGUUA
AAAGGUAUUGAUUUUAAAGAAGAUGGAAACAUUCUUGGACACAAAUUGGAAUACAACUAUAAC
UCACACAAUGUAUACAUCAUGGCAGACAAACAAAAGAAUGGAAUCAAAGUUAACUUCAAAAUUA
GACACAACAUUGAAGAUGGAAGCGUUCAACUAGCAGACCAUUAUCAACAAAAUACUCCAAUUG
GCGAUGGCCCUGUCCUUUUACCAGACAACCAUUACCUGUCCACACAAUCUGCCCUUUCGAAA
GAUCCCAACGAAAAGAGAGACCACAUGGUCCUUCUUGAGUUUGUAACAGCUGCUGGGAUUAC
ACAUGGCAUGGAUGAACUAUACAAAUAA

Nanoluciferase mRNA sequence

AUGGUCUUCACACUCGAAGAUUUCGUUGGGGACUGGCGACAGACAGCCGGCUACAACCUGGA
CCAAGUCCUUGAACAGGGAGGUGUGUCCAGUUUGUUUCAGAAUCUCGGGGUGUCCGUAACUC
CGAUCCAAAGGAUUGUCCUGAGCGGUGAAAAUGGGCUGAAGAUCGACAUCCAUGUCAUCAUC
CCGUAUGAAGGUCUGAGCGGCGACCAAAUGGGCCAGAUCGAAAAAAUUUUUAAGGUGGUGUA
CCCUGUGGAUGAUCAUCACUUUAAGGUGAUCCUGCACUAUGGCACACUGGUAAUCGACGGGG
UUACGCCGAACAUGAUCGACUAUUUCGGACGGCCGUAUGAAGGCAUCGCCGUGUUCGACGGC
AAAAAGAUCACUGUAACAGGGACCCUGUGGAACGGCAACAAAAUUAUCGACGAGCGCCUGAUC
AACCCCGACGGCUCCCUGCUGUUCCGAGUAACCAUCAACGGAGUGACCGGCUGGCGGCUGU
GCGAACGCAUUCUGGCGUGAUAA

General RNA/DNA 2'-OH Acylation

RNA1 oligonucleotides (50 μM, 282.5 ng/μL) were dissolved in DEPC-treated H_2O (32 μL) and added to solutions of **3**, **6**, **10**, and **13** in dry DMSO (1M, 2M, or 4M), to a total volume of 40 μL. **DNA1** oligonucleotides (50 μM , 270.2 ng/μL) were dissolved in DEPC-treated H_2O (32 μL) and added to 4 M dry DMSO solutions of **3**, **6**, **10**, and **13** respectively, to a total volume of 40 μL. **RNA2** oligonucleotides (50 μM, 295.2 ng/μL) were dissolved in DEPC-treated H_2O (32 μL) and added to solutions of **3**, **6**, **10**, and **13** in dry DMSO (1M, 2M, or 4M), to a total volume of 40 μL. For unmodified RNA and DNA control samples, an equivalent volume of dry DMSO was added in the absence of the acylating reagent. The mixtures were incubated for 24 hr, 48 hr or 72 hr at room temperature. After this time the acylation reactions were quenched with the addition of 3 M NaOAc (pH = 5.2, 0.1 equiv), RNA-grade glycogen (20 mg/mL, 1.5 μL) and ice-cold 100 % EtOH (110 μL). RNA and DNA was precipitated at -80 °C overnight. RNA and DNA pellets were obtained by centrifuging (14,000 rpm) for 30 minutes at 4 °C and removing the supernatant. The pellets were washed three times with ice-cold 70 % EtOH (500 μL) and centrifuged (12,500 rpm) for 15 minutes at 4 °C. The supernatant was removed, and samples were air-dried for 30 minutes. RNA and DNA pellets were stored at -40 °C and -20 °C respectively and later re-suspended in DEPC-treated H_2O for further analysis.

General RNA 2'-Deprotection

RNA samples in DEPC-treated H_2O were treated in a 1:1 ratio by volume with 20 mM L-glutathione in 20 mM sodium phosphate buffer (pH = 7.4). The samples were incubated at 37 °C (250 rpm) over a 72-hour period. Aliquots were obtained from the reaction mixture at 1, 2, 4, 8, 12, 24, 48 & 72 hours, and 2'-deprotection was quenched with the addition of 3 M NaOAc (pH = 5.2, 0.1 equiv), RNA-grade glycogen (20 mg/mL, 0.5 μL) and ice-cold 100 % EtOH (110 μL). Reaction aliquots were precipitated at -80 °C overnight. RNA pellets were obtained by centrifuging (14,000 rpm) for 30 minutes at 4 °C and removing the supernatant. The pellets were washed three times with ice-cold 70 % EtOH (500 μL) and centrifuged (12,500 rpm) for 15 minutes at 4 °C. The supernatant was removed, and samples were air-dried for 30 minutes. RNA pellets were stored at -40 °C and later re-suspended in DEPC-treated H_2O for further analysis.

General mRNA 2'-OH Acylation

In vitro transcribed mRNA samples encoding for green-fluorescent protein (60 µL,150 ng/µL) were resuspended in DEPC-treated H_2O and added to 2M solutions of **3**, **6**, **10**, and **13** in dry DMSO, to a total volume of 80 µL. The mixtures were incubated for 2 hours at room temperature. After this time the acylation reactions were quenched and mRNA was purified using the RNA Clean & Concentrator™-25 Kit (Zymo Research, Cat. #R1018) according to the manufacturer's recommendations.

In vitro transcribed mRNA samples encoding for nanoluciferase protein (60 µL, 200 ng/µL) were resuspended in DEPC-treated H_2O and added to 2M solutions of **3**, **6**, **10**, and **13** in dry DMSO, to a total volume of 80 µL. The mixtures were incubated for 2 hours at room temperature. After this time the acylation reactions were quenched and mRNA was purified using the RNA Clean & Concentrator™-25 Kit (Zymo Research, Cat. #R1018) according to the manufacturer's recommendations.

MALDI-ToF Mass Spectrometry Analysis

Matrix Assisted Laser Desorption Ionisation Time-of-Flight (MALDI-ToF) spectra were acquired using a Bruker autoFlex maX MALDI-ToF/ToF instrument using a polished steel MTP384 target plate. The instrument was operated in linear negative mode for oligonucleotide analysis. The UV nitrogen laser operated at 337 nm. 0.3 M trihydroxyacetophenone in EtOH (matrix) and 0.1 M aqueous ammonium citrate (co-matrix) were mixed in a 2:1 ratio by volume to be used as a matrix mix and were always freshly prepared before analysis. Oligonucleotide samples dissolved in DEPC-treated H_2O were mixed in a 1:1 ratio by volume with the matrix mix. 1 µL of this solution was then transferred to the target plate and dried uniformly by evaporation at room temperature. The instrument was calibrated using a mixture of DNA oligonucleotides across a *m/z* range of 3700–8600 Da. The spectral data was recorded using FlexControl software (Bruker), and analysed using FlexAnalysis (Bruker) and mMass software.

Urea-PAGE

Oligonucleotide samples were diluted in a 1:1 ratio by volume with Novex TBE 2X Sample buffer (Thermo Fisher Scientific), heated to 95 ˚C for 3 minutes and separated on 20 % acrylamide 9.6 M urea gels (5.8 g urea, 5.4 mL 40% acrylamide-bisacrylamide 29:1, 100 µL ammonium persulfate, 10 µL tetramethylethylenediamiene, 1.4 mL 10X TBE (21.6 g Tris base, 11.0 g boric acid, 8 mL 0.5 M EDTA pH 8.0 ± 0.1), 2.6 mL deionised water) in 1X TBE. The gels were pre-run at 150 V for 30 minutes and run at 150 V for between 75 and 90 minutes alongside bromophenol blue and xylene cyanol as markers for 8 and 28 nucleotides, respectively. The gels were stained with 1X SYBR™ Gold Nucleic Acid Gel Stain (Thermo Fisher Scientific) and imaged using a Geneflash Syngene Bio Imaging Gel Doc System & UV / White Transilluminator equipped with ImageJ software.

RNase T1 Gel Electrophoresis Assay

Unmodified and Acyl-RNA samples (200 ng) were dissolved in DEPC-treated H_2O and mixed in a 1:1 ratio by volume with RNAse T1 enzyme (7500 U, Thermo Fisher Scientific). The reactions were incubated at room temperature for 20 minutes before immediately being placed on ice. The reaction mixture was further diluted in a 1:1 ratio by volume with Novex TBE 2X Sample buffer (Thermo Fisher Scientific) and separated on 30 % acrylamide 9.6 M urea gels (5.8 g urea, 8 mL 40% acrylamide-bisacrylamide 29:1, 100 µL ammonium persulfate, 10 µL tetramethylethylenediamiene, 1.4 mL 10X TBE (21.6 g Tris base, 11.0 g boric acid, 8mL 0.5 M EDTA pH 8.0 ± 0.1)) in 1X TBE. The gels were pre-run at 150 V for 30 minutes and run at 150 V for between 45 and 60 minutes alongside bromophenol blue and xylene cyanol as markers for 8 nt and 28 nt, respectively. A ladder consisting of a mixture of RNA oligonucleotides (20 uM) of lengths 15 nt, 9 nt and 6 nt was loaded in Lane 1. The gels were stained with 1X SYBR™ Gold Nucleic Acid Gel Stain (Thermo Fisher Scientific) and imaged using a Geneflash Syngene Bio Imaging Gel Doc System & UV / White Transilluminator equipped with ImageJ software.

RNase A Gel Electrophoresis Assay

Unmodified and Acyl-RNA samples (200 ng) were dissolved in DEPC-treated H2O, diluted in a 1:1 ratio by volume with Novex TBE 2X Sample buffer (Thermo Fisher Scientific) and heated to 95 °C for 3 minutes. The samples were immediately placed on ice for 5 minutes and subsequently mixed with RNase A enzyme (1 µL, 1 µg/mL) before immediately being separated on 20 % acrylamide 9.6 M urea gels (5.8 g urea, 5.4 mL 40% acrylamide-bisacrylamide 29:1, 100 µL ammonium persulfate, 10 µL tetramethylethylenediamiene, 1.4 mL 10X TBE (21.6 g Tris base, 11.0 g boric acid, 8 mL 0.5 M EDTA pH 8.0 ± 0.1), 2.6 mL deionised water) in 1X TBE. The gels were pre-run at 150 V for 30 minutes and run at 150 V for between 45 and 60 minutes alongside bromophenol blue and xylene cyanol as markers for 8 nt and 28 nt, respectively. The gels were stained with 1X SYBRTM Gold Nucleic Acid Gel Stain (Thermo Fisher Scientific) and imaged using a Geneflash Syngene Bio Imaging Gel Doc System & UV / White Transilluminator equipped with ImageJ software.

Fetal Bovine Serum Electrophoresis Assay

Unmodified and Acyl-RNA samples (200 ng) were dissolved in DEPC-treated H2O, diluted in a 1:1 ratio by volume with Novex TBE 2X Sample buffer (Thermo Fisher Scientific) and heated to 95 °C for 3 minutes. The samples were immediately placed on ice for 5 minutes and subsequently mixed with 2 uL of Dulbecco's Modified Eagle Medium (DMEM; Gibco) supplemented with 2 mM L glutamine, 10% fetal bovine serum (FBS), 100 U/mL penicillin, and 0.1 mg/mL streptomycin, before immediately being separated on 20 % acrylamide 9.6 M urea gels (5.8 g urea, 5.4 mL 40% acrylamide-bisacrylamide 29:1, 100 µL ammonium persulfate, 10 µL tetramethylethylenediamiene, 1.4 mL 10X TBE (21.6 g Tris base, 11.0 g boric acid, 8 mL 0.5 M EDTA pH 8.0 ± 0.1), 2.6 mL deionised water) in 1X TBE. The gels were pre-run at 150 V for 30 minutes and run at 150 V for between 45 and 60 minutes alongside bromophenol blue and xylene cyanol as markers for 8 nt and 28 nt, respectively. The gels were stained with 1X

SYBRTM Gold Nucleic Acid Gel Stain (Thermo Fisher Scientific) and imaged using a Geneflash Syngene Bio Imaging Gel Doc System & UV / White Transilluminator equipped with ImageJ software.

In Vitro Transcription

Coding sequences for GFP and NanoLuc luciferase were cloned into the Takara IVT template vector (Takara, Cat. #6146). Plasmid DNA was linearized with HindIII and purified prior to transcription. IVT reactions (20 µL) were assembled using the IVTpro™ mRNA Synthesis Kit (Takara, Cat. #6141) according to the manufacturer's recommendations. Each reaction contained up to 1 µg linearized DNA template, 100 mM of each NTP, Clean Cap AG (TriLink Biotechnologies, N7113), 2 µL enzyme mix, and 2 µL transcription buffer. Reactions were incubated at 37 °C for 2 h. Residual DNA was removed by treatment with Turbo DNase (1 µL, 2 U; Thermo Fisher Scientific) for 15 minutes at 37 °C. For synthesis of modified mRNAs, N1-methylpseudouridine triphosphate (TriLink Biotechnologies, N1019) was substituted for UTP at an equimolar concentration. Following DNase digestion, transcripts were purified using the RNeasy Plus Kit (Qiagen, Cat. #74134) according to the manufacturer's instructions. Purified mRNAs were quantified, assessed for integrity, aliquoted, and stored at −80 °C until use.

Automated Tapestation Workflow for RNA Integrity Analysis

mRNA samples were mixed with Agilent RNA Sample Buffer at the recommended ratio. The Agilent RNA Ladder was prepared using the same buffer. All tubes were incubated at 72 °C for 3 min to denature RNA structures and immediately cooled to room temperature. Samples and ladder were loaded into Agilent RNA ScreenTape consumables and processed on the Agilent Tapestation automated electrophoresis platform. The instrument generated electropherograms and virtual gel images, which were analysed using Tapestation Analysis Software to evaluate RNA integrity, peak sharpness, and the presence or absence of low-molecular-weight degradation fragments.

Cell Culture & mRNA Transfection

HEK293 cells were maintained in Dulbecco's Modified Eagle Medium (DMEM; Gibco) supplemented with 2 mM L-Glutamine, 10% fetal bovine serum (FBS), 100 U/mL penicillin, and 0.1 mg/mL streptomycin. Cells were cultured at 37 °C in a humidified incubator with 5% CO_2.

For mRNA transfection, cells were seeded in 24 well plates and allowed to adhere for 24 hours. 250 ng IVT mRNA was transfected per well using Lipofectamine 2000 (Thermo Fisher Scientific) following the manufacturer's protocol. Lipid–RNA complexes were prepared in Opti-MEM™ Reduced Serum Medium (Gibco, Cat. #31985062) in a 0.5 µL:250 ng lipid:RNA ratio and added directly to cells maintained in complete DMEM (10% FBS). Transfection conditions and incubation times were optimized as required and are specified in the figure legends.

MTT Assay

HEK293 cells were transfected with GFP mRNA and NanoLuc mRNA constructs modified with reagents **3**, **6**, **10**, and **13**. After 24 h, the cells were detached by trypsinisation, seeded into 96-well tissue culture plates at a density of 2×10^4 cells per well, and incubated overnight at 37 °C. Cell viability was assessed on day 1, day 3, and day 6 using the MTT assay. At each time point, 50 µl per well of a 5 mg/ml MTT solution (3-(4,5-dimethylthiazol-2-yl)-2,5-diphenyl tetrazolium bromide) was added directly to the culture medium. Plates were wrapped in aluminium foil to protect the reaction from light and incubated for 4 h at 37 °C. Following incubation, the medium containing residual MTT was removed, and 175 µl per well of DMSO followed by 25 µl per well of Sorensen's glycine buffer (0.1 M glycine, 0.1 M NaCl, pH 10.5) were added to solubilise the formazan crystals. Absorbance was measured at 540 nm using a Multiskan Ascent plate reader (Thermo Labsystems, UK).

Luciferase Activity Assay

Secreted NanoLuc activity was measured at the indicated time points post-transfection using the Nano-Glo® Luciferase Assay System (Promega, Cat. #N1110) according to the manufacturer's instructions. For each sample, Nano-Glo® reagent was mixed 1:1 with culture medium collected from transfected cells. Luminescence was recorded on a microplate luminometer using standard settings. All measurements were performed in technical duplicate or triplicate and background luminescence from untransfected controls was subtracted.

Flow Cytometry

GFP expression was quantified at 24, 48, 72, and 120 hours post-transfection. At each time point, cells were detached with trypsin, collected by centrifugation (1200 rpm, 5 minutes), washed once with phosphate buffered saline (PBS), and resuspended in PBS for analysis. Flow cytometry was performed on a BD Accuri™ C6 cytometer equipped with a 488 nm laser; GFP fluorescence was detected in the FL1 channel (533/30 nm). A minimum of 10,000 events were recorded per sample. Data were analysed using BD Accuri C6 software; gating excluded debris and doublets and viable, single cells were used to quantify the percentage of GFP positive cells. Representative gating strategies are provided in the Figure S9.

Quantitative Reverse Transcription Polymerase Chain Reaction

Total RNA was isolated at 24, 48, 72, and 120 hours post-transfection using the RNeasy mRNA Isolation Kit (Qiagen) following the manufacturer's protocol. qRT-PCR was performed using the Luna® Universal One Step RT qPCR Kit (New England Biolabs) with 50 ng total RNA per reaction. Each 20 µL reaction contained Luna Universal One Step Reaction Mix (2×), Luna WarmStart® RT Enzyme Mix (20×), and gene specific primers. Thermocycling was carried out on a QuantStudio 3 Real Time PCR System with the following program: reverse transcription 55 °C for 10 minutes, initial denaturation 95 °C for 1 minute, then 40 cycles of 95 °C for 10 seconds and 60 °C for 30 seconds. Fluorescence was recorded for each

cycle and quantification was performed using the instrument software. Relative expression was calculated using the ΔΔCt method with an appropriate housekeeping gene; primer sequences and housekeeping controls are listed in Supplementary Table S1.

Statistical Analysis

Data are presented as mean ± standard deviation (SD) from at least three independent experiments (*n*) unless otherwise stated. Comparisons between two groups were performed using an unpaired two-tailed Student's t-test. For comparisons among three or more groups, one-way ANOVA followed by Tukey's post-hoc test was used. Statistical analyses were performed using GraphPad Prism (Version 10.6.1). A $p \leq 0.05$ was considered statistically significant. Exact *n* values and details of statistical tests are reported in the figure legends.

Table S1: qRT-PCR Primer Sequences

GFP_F- **5′→3′-**ATGAGTAAAGGAGAAGAACT

GFP_R- **5′→3′**-TAGTGCAAATAAATTTAAGG

Beta-actin_F- **5′→3′**-AGAGCTACGAGCTGCCTGAC

Beta-actin_R- **5′→3′**-AGCACTGTGTTGGCGTACAG

TNF-alpha_F- **5′→3′**-ATGATCCGGGACGTGGAGCT

TNF-alpha_R- **5′→3′**-TCTGGGGGCCGATCACTCCAAAG

IFN-alpha_F- **5′→3′**-CTCCCTGAGACCCACAGCCT

IFN-alpha_R- **5′→3′**-GTTGCCATCAAACTCCTCCTGG

Synthesis of Acylating Reagents

4-(Pyridin-2-yldisulfaneyl)butanoic acid (2)

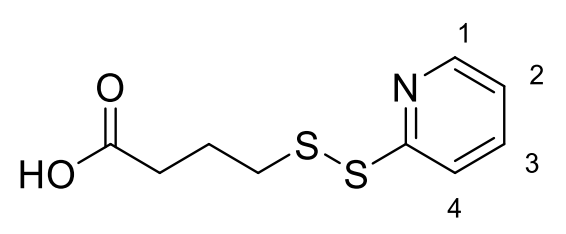

To a stirred solution of 2,2-dipyridyl disulfide (330.0 mg, 1.50 mmol) in MeOH (4 mL) was added 4-mercaptobutyric acid (**1**) (103.5 μL, 998.62 μmol). The reaction mixture was stirred for 8 h at rt under N_2, with an observed colour change from a colourless to bright yellow solution. After this time TLC analysis (cyclohexane:EtOAc, 3:1) showed consumption of starting material (R_f = 0.2) and formation of product (R_f = 0.4). The solvent was removed under reduced pressure and the residue was purified by flash chromatography (cyclohexane:EtOAc, 3:1) to provide product **2** (176.3 mg, 77%) as a white powder. A side product, 2-mercaptopyridine was obtained in a trace amount as a yellow powder.

ν_{max}/cm^{-1} (neat) 3138, 3109, 2962, 2916, 2849, 2511, 1710, 1578.

^{1}H NMR (400 MHz, DMSO-d_6) δ 1.81 (app p, *J* = 7.3 Hz, 2H, βCH_2) 2.31 (t, *J* = 7.3 Hz, 2H, αCH_2) , 2.82 (m, *J* = 7.2 Hz, 2H, γCH_2), 7.21 (dd, *J* = 7.2 Hz, 5.3 Hz, 1H, pyr-H2), 7.73 (app d, *J* = 7.95 Hz, 1H, pyr-H4), 7.80 (m, 1H, pyr-H3), 8.42 (dd, *J* = 5.3 Hz, 2.0 Hz, 1H, pyr-H1), 12.08 (bs, 1H, OH) ppm.

^{13}C NMR (101 MHz, DMSO-d_6) δ 24.4 (βCH_2), 32.6 (αCH_2), 37.8 (γCH_2), 119.8 (pyr-H4), 121.7 (pyr-H2), 138.3 (pyr-H3), 150.1 (pyr-H1), 159.7 (qC-pyr), 174.3 (COOH) ppm.

LC/MS (ESI$^+$) *m/z* calc. for $C_9H_{11}NO_2S_2$ = 230.0304, $(M+H)^+$, found = 230.0307.

1-(1H-Imidazol-1-yl)-4-(pyridin-2-yldisulfaneyl)butan-1-one (3)

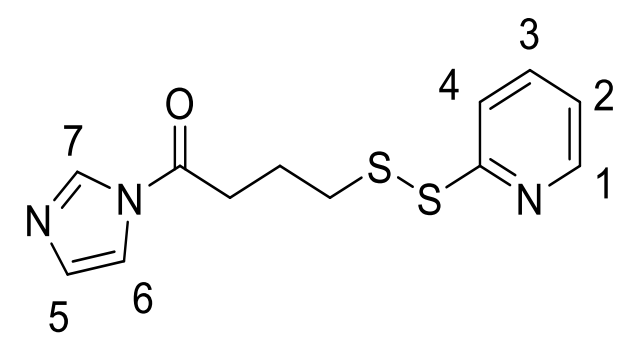

To a stirred solution of 4-(pyridin-2-yldisulfaneyl)butanoic acid (**2**) (100.1 mg, 436.1 μmol) in dry CH_2Cl_2 (4 mL) was added 1,1-carbonyl diimidazole (106.1 mg, 654.1 μmol). The reaction mixture was stirred for 2 h at rt under N_2. After this time TLC analysis (cyclohexane:EtOAc, 1:1) showed complete consumption of starting material (R_f = 0.2) and formation of product (R_f = 0.25). The solvent was removed under reduced pressure to afford **3** in quantitative yield as a pale-yellow solid. The crude product was used in subsequent RNA reactions without further purification.

^{1}H NMR (400 MHz, $CDCl_3$) δ 2.22 (p, *J* = 6.9 Hz, 2H, βCH_2), 2.93 (t, *J* = 6.9 Hz, 2H, αCH_2), 3.06 (t, *J* = 7.1 Hz, 2H, γCH_2), 7.06 – 7.10 (m, 2H, im-H5, pyr-H2), 7.46 (app t, *J* = 1.6 Hz, 1H, im-H6), 7.58 – 7.66 (m, 2H, pyr-H3,H4), 8.16 (s, 1H, im-H7), 8.42 (d, *J* = 4.8 Hz, 1H, pyr-H1) ppm.

^{13}C NMR (101 MHz, $CDCl_3$) δ 22.6 (βCH_2), 33.2 (γCH_2), 37.4 (αCH_2), 116.0 (im-H6), 120.2 (pyr-H4), 121.0 (pyr-H2), 131.1 (im-H5), 135.2 (im-H7), 137.1 (pyr-H3), 149.7 (pyr-H1), 159.4 (qC-pyr), 168.7 (CONCH_2) ppm.

LC/MS (ESI$^+$) *m/z* calc. for $C_{12}H_{13}N_3OS_2$ = 280.0573, $(M+H)^+$, found = 280.0573.

2-(Pyridin-2-yldisulfaneyl)ethan-1-ol (5)

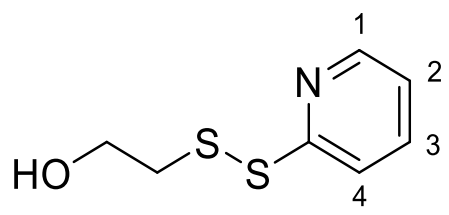

Compound **5** was synthesised according to modified literature procedures.[59-61] To a stirred solution of 2,2-dipyridyl disulfide (422.9 mg, 1.92 mmol) in MeOH (5 mL) was added 2-mercaptoethanol (**4**) (90.1 μL, 1.28 mmol) dropwise over 5 minutes at 0 °C. The reaction mixture was then allowed to warm to room temperature and stirred for 8 hr at rt under N_2, with an observed colour change from a colourless to bright yellow solution. After this time TLC analysis (cyclohexane:EtOAc, 1:1) showed consumption of starting material (R_f = 0.15) and formation of product (R_f = 0.4). The solvent was removed under reduced pressure and the crude product was purified by flash chromatography (cyclohexane:EtOAc, 3:2) to afford **5** (142.3 mg, 59 %) as a colourless oil. A side product, 2-mercaptopyridine was obtained in a trace amount as a yellow powder.

v_{max}/cm^{-1} (neat) 3288, 3046, 2922, 2864, 1575.

^{1}H NMR (400 MHz, $CDCl_3$) δ 2.91-2.95 (m, 2H, SCH_2), 3.76-3.81 (m, 2H, OCH_2), 7.13 (ddd, *J* = 7.4 Hz, 5.0 Hz, 0.9 Hz, 1H, pyr-H2), 7.38 (d, *J* = 8.1 Hz, 1H, pyr-H4), 7.52 – 7.62 (m, 1H, pyr-H3), 8.48 (d, *J* = 4.4 Hz, 1H, pyr-H1) ppm.

^{13}C NMR (101 MHz, $CDCl_3$) δ 42.7 (SCH_2), 58.2 (OCH_2), 121.5 (pyr-H2), 121.9 (pyr-H4), 136.8 (pyr-H3), 149.8 (pyr-H1), 159.1 (qC-pyr) ppm.

LC/MS (ESI$^+$) *m/z* calc. for $C_7H_9NO_2$ = 188.0198, $(M+H)^+$, found = 188.0199.

Spectral data is consistent with literature reports.[61]

2-(Pyridin-2-yldisulfaneyl)ethyl 1H-imidazole-1-carboxylate (6)

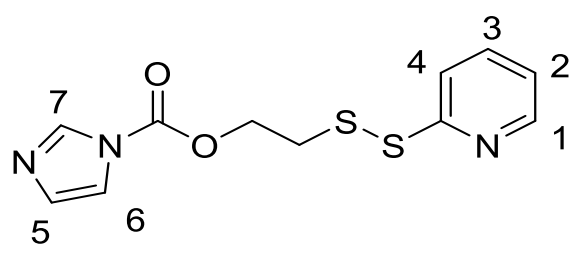

Compound **6** was synthesised according to a modified literature procedure.[62] To a stirred solution of 2-(pyridin-2-yldisulfaneyl)ethan-1-ol (**5**) (130.0 mg, 694.17 µmol) in dry CH_2Cl_2 (5 mL) was added 1,1-carbonyl diimidazole (168.8 mg, 1.04 mmol). The reaction mixture was then stirred for 12 h at rt under N_2. After this time TLC analysis (cyclohexane:EtOAc, 1:1) showed complete consumption of starting material (R_f = 0.45) and formation of product (R_f = 0.2). The solvent was removed under reduced pressure to afford **6** in quantitative yield as a pale-yellow solid. The crude product was used in subsequent RNA reactions without further purification.

v_{max}/cm^{-1} (neat) 3123, 3013, 2912, 2821, 2683, 2608, 1821, 1668.

^{1}H NMR (400 MHz, DMSO-d$_6$) δ 3.25 (t, *J* = 6.0 Hz, 2H, SCH_2), 4.57 (t, *J* = 6.0 Hz, 2H, OCH_2), 7.04 (m, 1H, im-H5), 7.19 (q, *J* = 4.4 Hz, 1H, pyr-H2), 7.52 (app t, *J* = 1.3 Hz, 1H, im-H6), 7.70 -7.75 (m, 2H, pyr-H3,H4), 8.18 (s, 1H, im-H7), 8.39 (m, 1H, pyr-H1) ppm.

^{13}C NMR (101 MHz, DMSO-d$_6$) δ 36.9 (SCH_2), 66.1 (OCH_2), 117.9 (im-H6), 120.0 (pyr-H4), 121.8 (pyr-H2), 130.8 (im-H5), 137.6 (im-H7), 138.2 (pyr-H3), 148.5 (COON), 150.1 (pyr-H1), 159.0 (qC-pyr) ppm.

LC/MS (ESI$^+$) *m/z* calc. for $C_{11}H_{11}N_3O_2S_2$ = 282.0365, $(M+H)^+$, found = 282.0364.

5-Mercaptopentanoic acid (8)

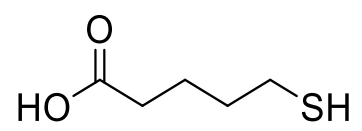

Compound **8** was synthesised according to a modified literature procedure.[26] To a stirred solution of 5-bromopentanoic acid (**7**) (1.81 g, 10 mmol) in EtOH (8 mL) was added thiourea (1.14 g, 15 mmol). The reaction mixture was refluxed for 20 h. After this time the solvent was removed under reduced pressure and 7.5 M sodium hydroxide solution (20 mL) was added. The solution was transferred to an oil bath and stirred at 90 °C for 16 h under N_2. Following this time, the reaction mixture was cooled with an ice bath and 6 M hydrochloric acid solution (50 mL) was added dropwise until an acidic pH was achieved. The mixture was transferred to a separating funnel and extracted with DCM (3 x 60 mL), and the organic layers were combined and dried over sodium sulphate. The solvent was removed under reduced pressure to afford **8** (804.2 mg, 60 %) as a pale yellow oil.

^{1}H NMR (400 MHz, $CDCl_3$) δ 1.35 (t, *J* = 7.8 Hz, 1H, SH), 1.66-1.81 (m, 4H, βCH_2 + γCH_2), 2.38 (t, *J* = 7.2 Hz, 2H, αCH_2), 2.54 (app q, *J* = 7.2 Hz, 2H, CH_2SH) ppm.

^{13}C NMR (101 MHz, $CDCl_3$) δ 23.3 (βCH_2), 24.2 (CH_2SH), 33.2 (γCH_2), 33.3 (αCH_2), 187.7 (COOH) ppm.

LC/MS (ESI$^-$) *m/z* calc. for $C_5H_{10}O_2S$ = 133.0329, $(M-H)^-$, found = 133.0327.

Spectral data is consistent with literature reports.[63]

5-(Pyridin-2-yldisulfaneyl)pentanoic acid (9)

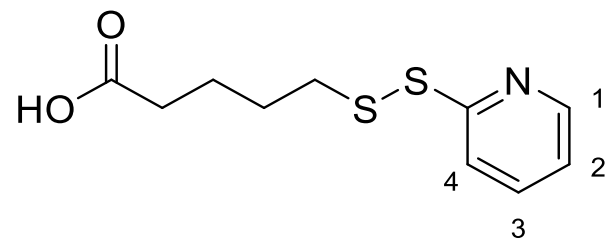

To a stirred solution of 2,2-dipyridyl disulfide (492.5 mg, 2.24 mmol) in MeOH (10 mL) was added Compound **8** (200.0 mg, 1.49 mmol). The reaction mixture was stirred for 24 h at rt under N_2, with an observed colour change from a colourless to bright yellow solution. After this time TLC analysis (cyclohexane:EtOAc, 1:1) showed consumption of starting material (R_f = 0.2) and formation of product (R_f = 0.3). The solvent was removed under reduced pressure, and the residue was purified by flash chromatography (cyclohexane:EtOAc, 3:1 → 3:2 → 1:1) to provide product **9** (188.7 mg, 52 %) as a pale-yellow oil. A side product, 2-mercaptopyridine was obtained in a trace amount as a yellow solid.

ν_{max}/cm^{-1} (neat) 3054, 2914, 2502, 1916, 1711, 1579.

^{1}H NMR (400 MHz, $CDCl_3$) δ 1.68-1.84 (m, 4H, βCH_2 + γCH_2), 2.36 (t, *J* = 6.9 Hz, 2H, αCH_2), 2.84 (t, *J* = 6.8 Hz, 2H, CH_2SS), 7.12-7.22 (m, 1H, pyr-H2), 7.61-7.83 (m, 2H, pyr-H3 + pyr-H4), 8.53 (d, *J* = 3.9 Hz, 1H, pyr-H1) ppm.

^{13}C NMR (101 MHz, $CDCl_3$) δ 23.4 (βCH_2), 28.2 (γCH_2), 33.2 (αCH_2), 38.4 (CH_2SS), 120.6 (pyr-H3), 121.0 (pyr-H2), 138.3 (pyr-H4), 148.4 (pyr-H1), 160.0 (qC-pyr), 177.4 (COOH) ppm.

LC/MS (ESI$^+$) *m/z* calc. for $C_{10}H_{13}NO_2S_2$ = 244.0460 $(M+H)^+$, found = 244.0461.

1-(1H-Imidazol-1-yl)-5-(pyridin-2-yldisulfaneyl)pentan-1-one (10)

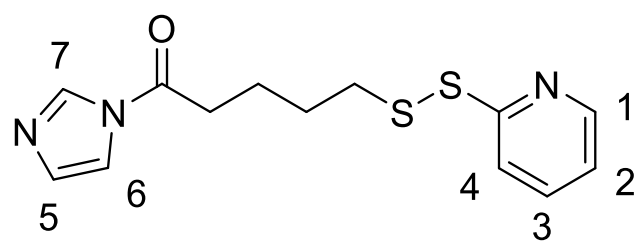

To a stirred solution of 5-(pyridin-2-yldisulfaneyl)pentanoic acid (**9**) (160.1 mg, 657.5 μmol) in dry CH_2Cl_2 (4 mL) was added 1,1-carbonyl diimidazole (160.0 mg, 986.3 μmol). The reaction mixture was stirred for 2 h at rt under N_2. After this time TLC analysis (cyclohexane:EtOAc, 1:1) showed complete consumption of starting material (R_f = 0.3) and formation of product (R_f = 0.2). The solvent was removed under reduced pressure to afford **10** in quantitative yield as a pale-yellow powder. The crude product was used in subsequent RNA reactions without further purification.

^{1}H NMR (400 MHz, $CDCl_3$) δ 1.79-1.98 (m, 4H, βCH_2 + γCH_2), 2.76-2.96 (m, 4H, αCH_2 + CH_2SS), 7.04 – 7.12 (m, 2H, im-H5, pyr-H2), 7.46 (d, *J* = 1.6 Hz, 1H, im-H6), 7.56 – 7.76 (m, 2H, pyr-H3 + pyr-H4), 8.15 (s, 1H, im-H7), 8.42-8.50 (m, 1H, pyr-H1) ppm.

^{13}C NMR (101 MHz, $CDCl_3$) δ 22.7 (βCH_2), 28.0 (γCH_2), 34.7 (αCH_2), 116.0 (im-H6), 119.9 (pyr-H4), 120.7 (pyr-H2), 131.1 (im-H5), 134.6 (im-H7), 137.0 (pyr-H3), 149.7 (pyr-H1), 159.1 (qC-pyr), 168.4 (<u>C</u>ONCH_2) ppm.

LC/MS (ESI$^+$) *m/z* calc. for $C_{13}H_{15}N_3OS_2$ = 294.0729, (M+H)$^+$, found = 294.0729.

3-(Pyridin-2-yldisulfaneyl)propan-1-ol (12)

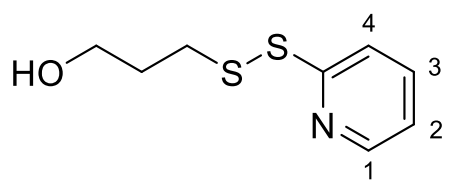

Compound **12** was synthesised according to a modified procedure.[60] To a stirred solution of 2,2-dipyridyl disulfide (896.5 mg, 4.07 mmol) in MeOH (5 mL) was added 3-mercaptopropanol (**11**) (235.9 µL, 2.71 mmol) dropwise over 5 minutes at 0 °C. The reaction mixture was then allowed to warm to room temperature and stirred for 6 h at rt under N_2. After this time TLC analysis (cyclohexane:EtOAc, 1:1) showed consumption of starting material (R_f = 0.2) and formation of product (R_f = 0.3). The solvent was removed under reduced pressure and the crude product was purified by flash chromatography (cyclohexane:EtOAc, 3:2) to afford **12** (189.7 mg, 34%) as a colourless oil. A side product, 2-mercaptopyridine was obtained in a trace amount as a yellow powder.

^{1}H NMR (400 MHz, DMSO-d_6) δ 1.68 – 1.80 (m, 2H, OCH_2<u>CH_2</u>), 2.85 (t, *J* = 7.0 Hz, 2H, SCH_2), 3.43 (q, *J* = 5.9 Hz, 2H, OCH_2), 7.20 (ddd, *J* = 7.4 Hz, 4.9 Hz, 1.3 Hz, 1H, pyr-H2), 7.70 – 7.77 (m, 1H, pyr-H4), 7.77 – 7.85 (m, 1H, pyr-H3), 8.39 – 8.45 (m, 1H, pyr-H1) ppm.

^{13}C NMR (101 MHz, DMSO-d_6) δ 32.2 (OCH_2<u>CH_2</u>), 35.4 (SCH_2), 59.4 (OCH_2), 119.6 (pyr-H4), 121.5 (pyr-H2), 138.2 (pyr-H3), 149.9 (pyr-H1), 159.8 (qC-pyr) ppm.

LC/MS (ESI$^+$) *m/z* calc. for $C_8H_{11}NOS_2$ = 202.0355, (M+H)$^+$, found = 202.0354.

Spectral data is consistent with literature reports.[64]

3-(Pyridin-2-yldisulfaneyl)propyl 1H-imidazole-1-carboxylate (13)

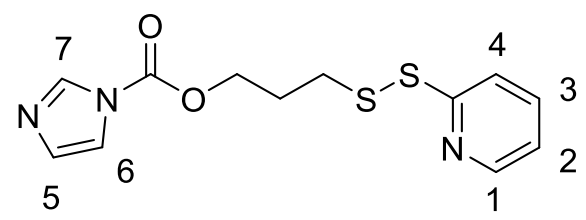

To a stirred solution of 3-(pyridin-2-yldisulfaneyl)propan-1-ol **(12)** (140.0 mg, 695.47 µmol) in dry CH_2Cl_2 (5 mL) was added 1,1-carbonyl diimidazole (191.7 mg, 1.18 mmol). The reaction mixture was then stirred for 12 h at rt under N_2. After this time TLC analysis (clyclohexane:EtOAc, 1:1) showed complete consumption of starting material (R_f = 0.3) and formation of product (R_f = 0.2). The solvent was removed under reduced pressure to afford **13** in quantitative yield as a white powder. The crude product was used in subsequent RNA reactions without further purification.

v_{max}/cm^{-1} (neat) 3125, 3111, 3012, 2916, 2790, 2695, 2610, 1821, 1668.

^{1}H NMR (400 MHz, DMSO-d$_6$) δ 2.02 – 2.13 (m, 2H, OCH$_2$$\underline{CH_2}$), 2.99 (t, *J* = 7.1 Hz, 2H SCH$_2$), 4.43 (t, *J* = 6.1 Hz, 2H, OCH$_2$), 7.03 (d, *J* = 1.7 Hz, 1H, im-H5), 7.17 – 7.24 (m, 1H, pyr-H2), 7.55 (t, *J* = 1.5 Hz, 1H, pyr-H4), 7.73 (app d, *J* = 7.9 Hz, 1H, im-H6), 7.76 – 7.85 (m, 1H, pyr-H3), 8.22 (s, 1H, im-H7), 8.42 (dd, *J* = 4.9 Hz, 1.8 Hz, 1H, pyr-H1) ppm.

^{13}C NMR (101 MHz, DMSO-d$_6$) δ 27.8 (OCH$_2$$\underline{CH_2}$), 34.7 (SCH$_2$), 66.8 (OCH$_2$), 118.0 (pyr-H4), 119.9 (im-H6), 121.7 (pyr-H2), 130.7 (im-H5), 137.7 (im-H7), 138.2 (pyr-H3), 148.7 (COON), 150.1 (pyr-H1), 159.4 (qC-pyr) ppm.

LC/MS (ESI$^+$) *m/z* calc. for $C_{12}H_{13}N_3O_2S_2$ = 296.0522, $(M+H)^+$, found = 296.0522.

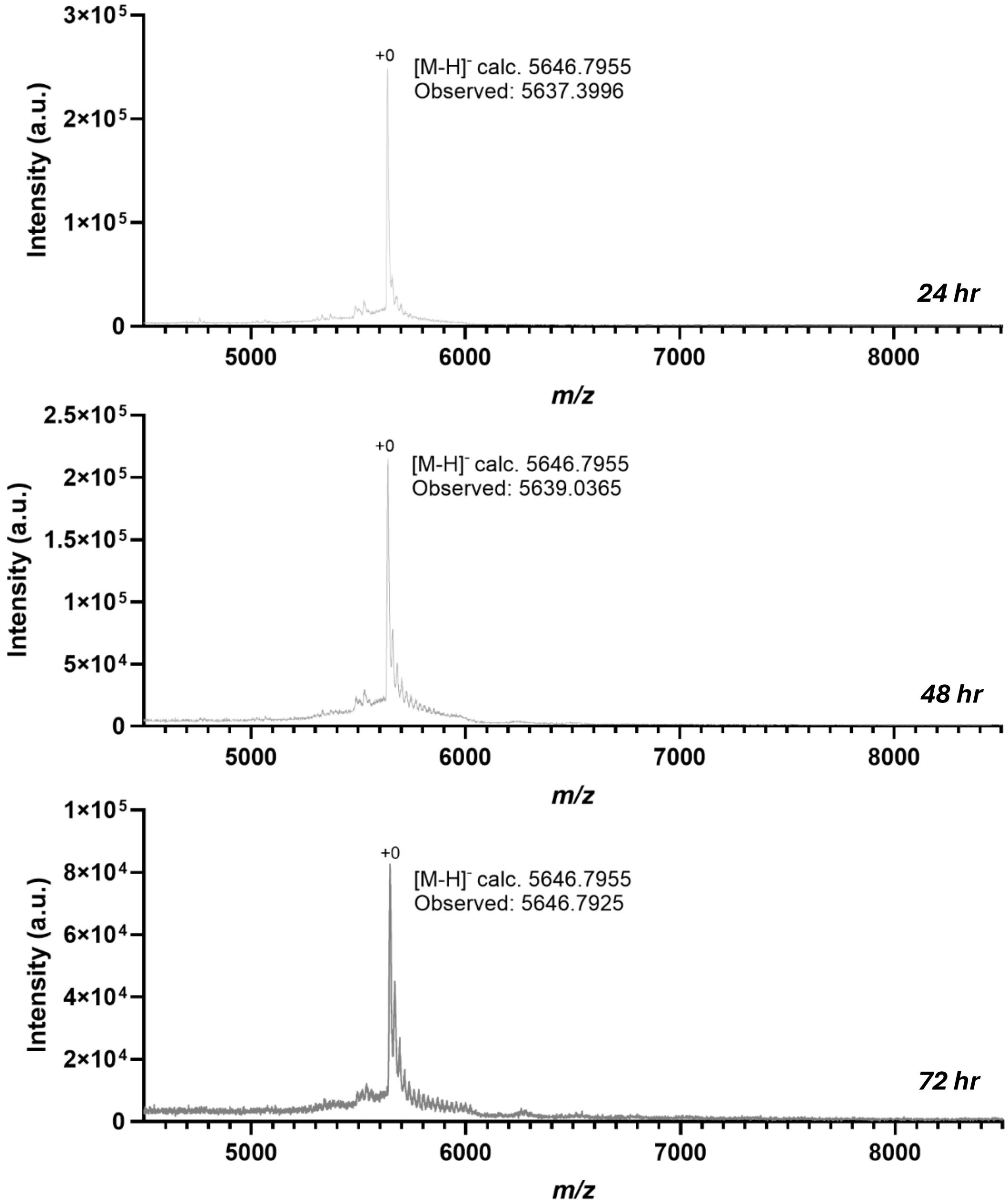

Figure S1A: MALDI-ToF mass spectra of unmodified, DMSO mock-treated **RNA1** incubated at room temperature for 24, 48 and 72 hours respectively.

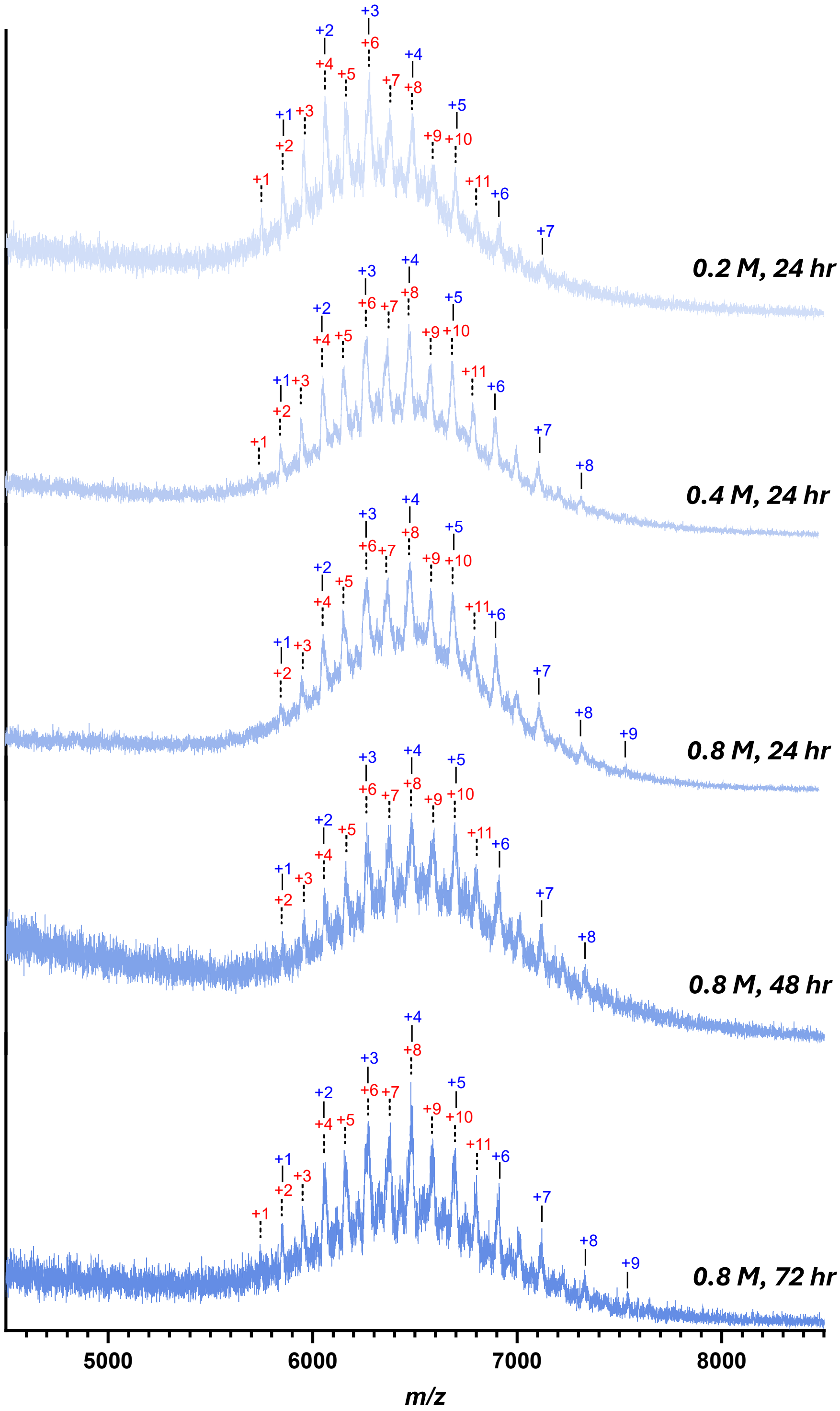

Figure S1B: MALDI-ToF mass spectra of RNA 2'-acylation reaction conditions to produce **3**-Acyl-RNA, with varying acylating reagent stock concentration in dry DMSO and duration of room temperature incubation. The number of intact disulfide conjugates (+X) are denoted in blue alongside the number of reduced thiol conjugates (+Y), denoted in red.

Table S2: Calculated and observed *m/z* values based on the above recorded MALDI-ToF spectra for **3**-Acyl-RNA models (Figure S1B). The number of intact disulfide conjugates (+X) are denoted in blue alongside the number of reduced thiol conjugates (+Y), denoted in red.

**3-Acyl-RNA:** 0.2 M DMSO, 24 hr					
+X Disulfide 2'-Conjugates	***Calculated [M-H]-***	**Observed [M-H]-**	***+Y Reduced Thiol 2'-Conjugates***	***Calculated [M-H]-***	**Observed [M-H]-**
+1	*5857.8081*	5857.8773	*+1*	*5748.8095*	5748.8094
+2	*6068.8207*	6067.4712	*+2*	*5850.8234*	5849.5825
+3	*6279.8332*	6279.3807	*+3*	*5952.8374*	5951.4906
+4	*6490.8458*	6489.9643	*+4*	*6054.8513*	6053.2362
+5	*6701.8583*	6699.0180	*+5*	*6156.8652*	6156.9872
+6	*6912.8709*	6912.8913	*+6*	*6258.8792*	6259.5258
+7	*7123.8834*	7121.5159	*+7*	*6360.8931*	6358.9833
			+8	*6462.9070*	6462.4617
			+9	*6564.9210*	6566.3155
			+10	*6666.9349*	6665.4209
			+11	*6768.9488*	6766.9761
**3-Acyl-RNA:** 0.4 M DMSO, 24 hr					
+X Disulfide 2'-Conjugates	***Calculated [M-H]-***	**Observed [M-H]-**	***+Y Reduced Thiol 2'-Conjugates***	***Calculated [M-H]-***	**Observed [M-H]-**
+1	*5857.8081*	5854.8457	*+1*	*5748.8095*	5751.3656
+2	*6068.8207*	6063.1436	*+2*	*5850.8234*	5852.6330
+3	*6279.8332*	6279.6434	*+3*	*5952.8374*	5952.7600
+4	*6490.8458*	6492.3350	*+4*	*6054.8513*	6056.2285
+5	*6701.8583*	6700.1326	*+5*	*6156.8652*	6157.4665
+6	*6912.8709*	6912.3779	*+6*	*6258.8792*	6258.9836
+7	*7123.8834*	7125.0639	*+7*	*6360.8931*	6359.9838
+8	*7334.8960*	7334.4528	*+8*	*6462.9070*	6461.4525
			+9	*6564.9210*	6567.6096
			+10	*6666.9349*	6665.8095
			+11	*6768.9488*	6764.8085
**3-Acyl-RNA:** 0.8 M DMSO, 24 hr					
+X Disulfide 2'-Conjugates	***Calculated [M-H]-***	**Observed [M-H]-**	***+Y Reduced Thiol 2'-Conjugates***	***Calculated [M-H]-***	**Observed [M-H]-**
+1	*5857.8081*	5858.7998	*+2*	*5850.8234*	5850.9589
+2	*6068.8207*	6067.2791	*+3*	*5952.8374*	5953.8890
+3	*6279.8332*	6278.3056	*+4*	*6054.8513*	6054.2637
+4	*6490.8458*	6490.6405	*+5*	*6156.8652*	6158.9249
+5	*6701.8583*	6699.8299	*+6*	*6258.8792*	6258.7200
+6	*6912.8709*	6913.7920	*+7*	*6360.8931*	6362.0938
+7	*7123.8834*	7124.5742	*+8*	*6462.9070*	6464.4490
+8	*7334.8960*	7334.3108	*+9*	*6564.9210*	6564.5610
+9	*7545.9085*	7547.8047	*+10*	*6666.9349*	6671.1612
			+11	*6768.9488*	6764.2013
**3-Acyl-RNA:** 0.8 M DMSO, 48 hr					
+X Disulfide 2'-Conjugates	***Calculated [M-H]-***	**Observed [M-H]-**	***+Y Reduced Thiol 2'-Conjugates***	***Calculated [M-H]-***	**Observed [M-H]-**
+1	*5857.8081*	5858.5717	*+2*	*5850.8234*	5852.7650
+2	*6068.8207*	6068.8663	*+3*	*5952.8374*	5951.3916
+3	*6279.8332*	6280.2185	*+4*	*6054.8513*	6054.3313
+4	*6490.8458*	6490.3877	*+5*	*6156.8652*	6158.0938
+5	*6701.8583*	6703.1179	*+6*	*6258.8792*	6258.2255
+6	*6912.8709*	6912.6191	*+7*	*6360.8931*	6358.8837
+7	*7123.8834*	7123.9357	*+8*	*6462.9070*	6463.4834
+8	*7334.8960*	7335.0720	*+9*	*6564.9210*	6563.8731
			+10	*6666.9349*	6669.8193
			+11	*6768.9488*	6767.4616
**3-Acyl-RNA:** 0.8 M DMSO, 72 hr					
+X Disulfide 2'-Conjugates	***Calculated [M-H]-***	**Observed [M-H]-**	***+Y Reduced Thiol 2'-Conjugates***	***Calculated [M-H]-***	**Observed [M-H]-**
+1	*5857.8081*	5855.0803	*+1*	*5748.8095*	5747.3629
+2	*6068.8207*	6067.5685	*+2*	*5850.8234*	5848.0334
+3	*6279.8332*	6275.9989	*+3*	*5952.8374*	5952.6122
+4	*6490.8458*	6488.7188	*+4*	*6054.8513*	6053.5930
+5	*6701.8583*	6698.0759	*+5*	*6156.8652*	6160.7496
+6	*6912.8709*	6911.4989	*+6*	*6258.8792*	6259.6297
+7	*7123.8834*	7120.5399	*+7*	*6360.8931*	6361.8974
+8	*7334.8960*	7334.1411	*+8*	*6462.9070*	6460.8097
+9	*7545.9085*	7543.6980	*+9*	*6564.9210*	6567.0828
			+10	*6666.9349*	6665.8164
			+11	*6768.9488*	6770.3653

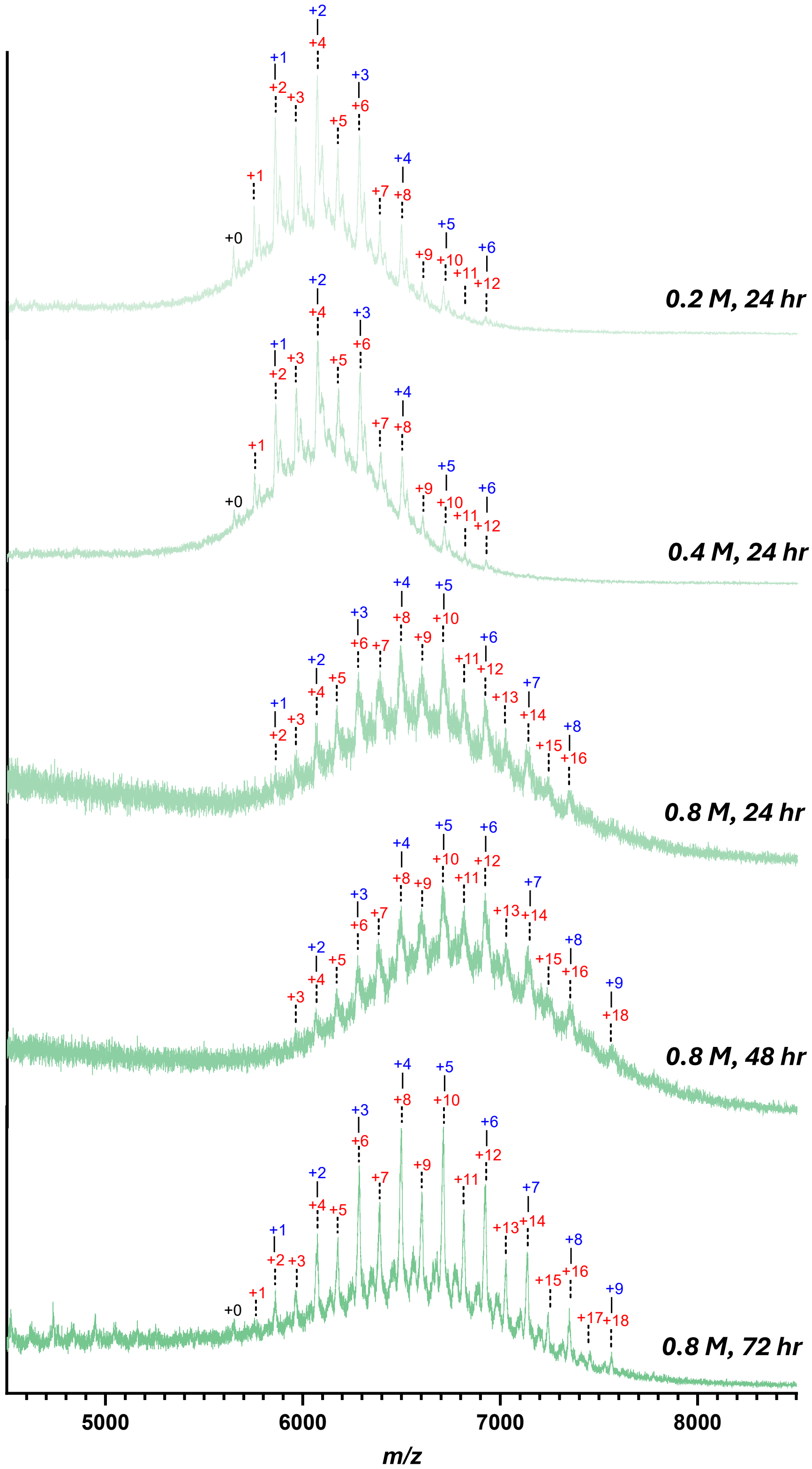

Figure S1C: MALDI-ToF mass spectra of RNA 2'-acylation reaction conditions to produce **6**-Acyl-RNA, with varying acylating reagent stock concentration in dry DMSO and duration of room temperature incubation. The number of intact disulfide conjugates (+X) are denoted in blue alongside the number of reduced thiol conjugates (+Y), denoted in red.

Table S3: Calculated and observed *m/z* values based on the above recorded MALDI-ToF spectra for **6**-Acyl-RNA models (Figure S1C). The number of intact disulfide conjugates (+X) are denoted in blue alongside the number of reduced thiol conjugates (+Y), denoted in red.

**6-Acyl-RNA:** 0.2 M DMSO, 24 hr					
+X Disulfide 2'-Conjugates	***Calculated*** $[M\text{-}H]^-$	**Observed** $[M\text{-}H]^-$	***+Y Reduced Thiol 2'-Conjugates***	***Calculated*** $[M\text{-}H]^-$	**Observed** $[M\text{-}H]^-$
+0	*5646.7955*	5646.9085	*+1*	*5750.7887*	5750.8420
+1	*5859.7874*	5859.8549	*+2*	*5854.7819*	5857.3035
+2	*6072.7792*	6070.5345	*+3*	*5958.7751*	5958.8545
+3	*6285.7710*	6284.0748	*+4*	*6062.7683*	6062.9924
+4	*6498.7628*	6497.5668	*+5*	*6166.7615*	6168.0580
+5	*6711.7546*	6709.5577	*+6*	*6270.7547*	6273.4240
+6	*6924.7465*	6922.3762	*+7*	*6374.7479*	6379.5686
			+8	*6478.7411*	6482.4932
			+9	*6582.7343*	6587.0609
			+10	*6686.7275*	6686.7562
			+11	*6790.7207*	6795.7793
			+12	*6894.7139*	6889.7663
**6-Acyl-RNA:** 0.4 M DMSO, 24 hr					
+X Disulfide 2'-Conjugates	***Calculated*** $[M\text{-}H]^-$	**Observed** $[M\text{-}H]^-$	***+Y Reduced Thiol 2'-Conjugates***	***Calculated*** $[M\text{-}H]^-$	**Observed** $[M\text{-}H]^-$
+0	*5646.7955*	5647.7112	*+1*	*5750.7887*	5752.1440
+1	*5859.7874*	5859.5653	*+2*	*5854.7819*	5854.1002
+2	*6072.7792*	6071.3899	*+3*	*5958.7751*	5959.9302
+3	*6285.7710*	6286.2596	*+4*	*6062.7683*	6065.5172
+4	*6498.7628*	6498.8646	*+5*	*6166.7615*	6165.9577
+5	*6711.7546*	6711.8371	*+6*	*6270.7547*	6272.3130
+6	*6924.7465*	6925.3410	*+7*	*6374.7479*	6378.6306
			+8	*6478.7411*	6483.6096
			+9	*6582.7343*	6582.3496
			+10	*6686.7275*	6685.4356
			+11	*6790.7207*	6789.5444
			+12	*6894.7139*	6898.8616
**6-Acyl-RNA:** 0.8 M DMSO, 24 hr					
+X Disulfide 2'-Conjugates	***Calculated*** $[M\text{-}H]^-$	**Observed** $[M\text{-}H]^-$	***+Y Reduced Thiol 2'-Conjugates***	***Calculated*** $[M\text{-}H]^-$	**Observed** $[M\text{-}H]^-$
+1	*5859.7874*	5858.1988	*+2*	*5854.7819*	5856.8940
+2	*6072.7792*	6070.7228	*+3*	*5958.7751*	5957.4128
+3	*6285.7710*	6286.2520	*+4*	*6062.7683*	6061.9800
+4	*6498.7628*	6498.3405	*+5*	*6166.7615*	6166.7941
+5	*6711.7546*	6709.1099	*+6*	*6270.7547*	6272.7227
+6	*6924.7465*	6921.5796	*+7*	*6374.7479*	6377.2093
+7	*7137.7383*	7135.1335	*+8*	*6478.7411*	6479.4136
+8	*7350.7301*	7350.6223	*+9*	*6582.7343*	6580.7721
			+10	*6686.7275*	6685.0345
			+11	*6790.7207*	6793.2037
			+12	*6894.7139*	6897.3673
			+13	*6998.7072*	6997.3081
			+14	*7102.7004*	7103.0719
			+15	*7206.6936*	7203.4375
			+16	*7310.6868*	7306.4289
**6-Acyl-RNA:** 0.8 M DMSO, 48 hr					
+X Disulfide 2'-Conjugates	***Calculated*** $[M\text{-}H]^-$	**Observed** $[M\text{-}H]^-$	***+Y Reduced Thiol 2'-Conjugates***	***Calculated*** $[M\text{-}H]^-$	**Observed** $[M\text{-}H]^-$
+2	*6072.7792*	6071.5541	*+3*	*5958.7751*	5960.4191
+3	*6285.7710*	6283.0840	*+4*	*6062.7683*	6062.1402
+4	*6498.7628*	6497.2432	*+5*	*6166.7615*	6168.1615
+5	*6711.7546*	6709.6129	*+6*	*6270.7547*	6273.3045
+6	*6924.7465*	6921.4750	*+7*	*6374.7479*	6373.7317
+7	*7137.7383*	7139.4034	*+8*	*6478.7411*	6478.9518
+8	*7350.7301*	7353.4671	*+9*	*6582.7343*	6581.8551
+9	*7563.7219*	7563.5533	*+10*	*6686.7275*	6687.6211
			+11	*6790.7207*	6789.1776
			+12	*6894.7139*	6897.5798
			+13	*6998.7072*	6994.8005
			+14	*7102.7004*	7106.4112
			+15	*7206.6936*	7204.9836
			+16	*7310.6868*	7312.2583
			+18	*7518.6732*	7521.6619
**6-Acyl-RNA:** 0.8 M DMSO, 72 hr					
+X Disulfide 2'-Conjugates	***Calculated*** $[M\text{-}H]^-$	**Observed** $[M\text{-}H]^-$	***+Y Reduced Thiol 2'-Conjugates***	***Calculated*** $[M\text{-}H]^-$	**Observed** $[M\text{-}H]^-$

+0	*5646.7955*	5647.8931	*+1*	*5750.7887*	5754.8057
+1	*5859.7874*	5860.6011	*+2*	*5854.7819*	5853.4992
+2	*6072.7792*	6074.0792	*+3*	*5958.7751*	5962.2212
+3	*6285.7710*	6285.7661	*+4*	*6062.7683*	6060.8989
+4	*6498.7628*	6498.1227	*+5*	*6166.7615*	6168.5240
+5	*6711.7546*	6711.3796	*+6*	*6270.7547*	6270.3334
+6	*6924.7465*	6924.1239	*+7*	*6374.7479*	6379.8322
+7	*7137.7383*	7136.8896	*+8*	*6478.7411*	6482.5628
+8	*7350.7301*	7349.9834	*+9*	*6582.7343*	6586.1019
+9	*7563.7219*	7564.5071	*+10*	*6686.7275*	6682.2455
			+11	*6790.7207*	6788.0694
			+12	*6894.7139*	6892.1616
			+13	*6998.7072*	7001.2261
			+14	*7102.7004*	7101.7667
			+15	*7206.6936*	7204.7959
			+16	*7310.6868*	7311.8833
			+17	*7414.6800*	7411.8944
			+18	*7518.6732*	7515.0964

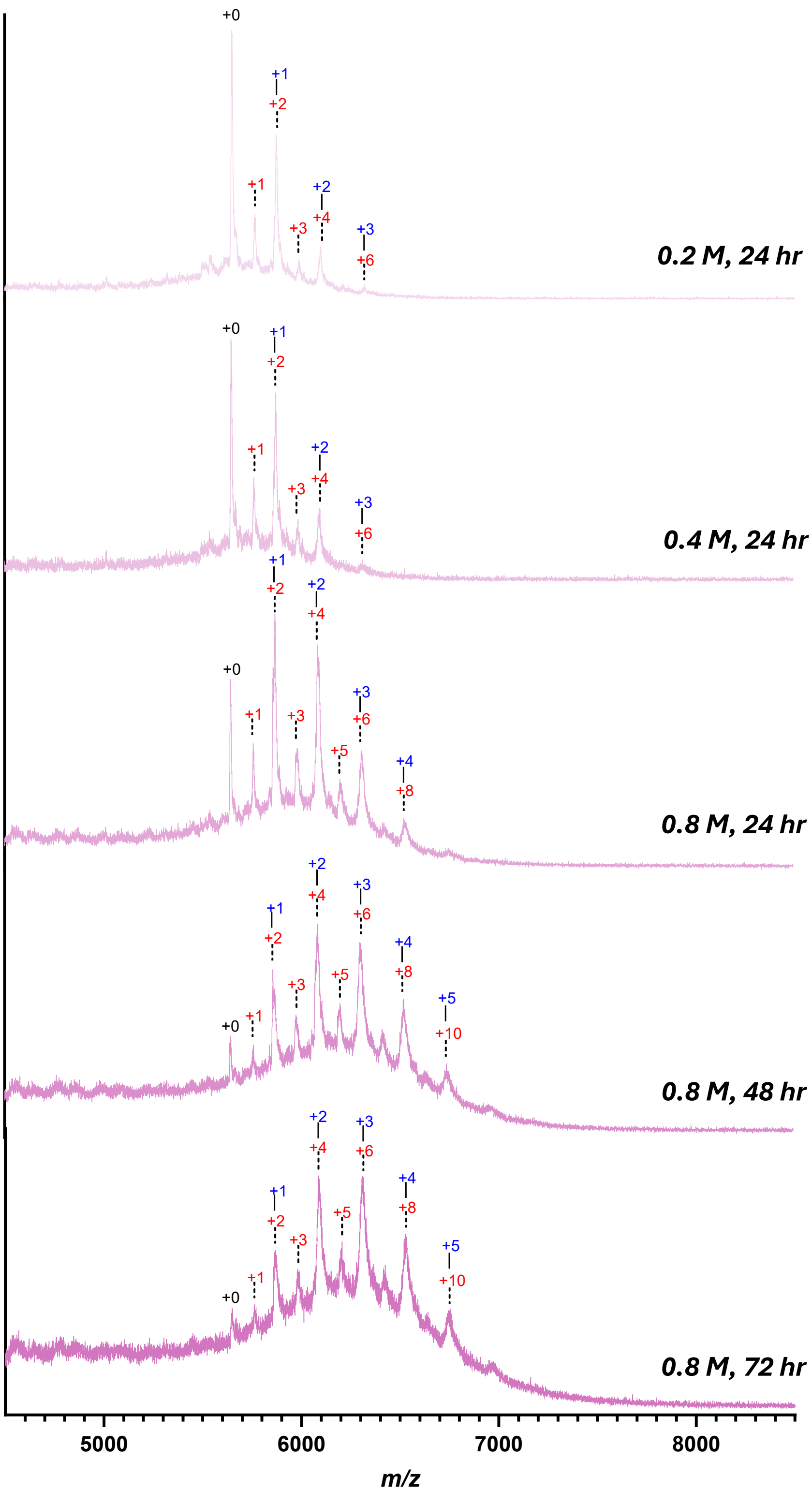

Figure S1D: MALDI-ToF mass spectra of RNA 2'-acylation reaction conditions to produce **10**-Acyl-RNA, with varying acylating reagent stock concentration in dry DMSO and duration of room temperature incubation. The number of intact disulfide conjugates (+X) are denoted in blue alongside the number of reduced thiol conjugates (+Y), denoted in red.

Table S4: Calculated and observed *m/z* values based on the above recorded MALDI-ToF spectra for **10**-Acyl-RNA models (Figure S1D). The number of intact disulfide conjugates (+X) are denoted in blue alongside the number of reduced thiol conjugates (+Y), denoted in red.

**10-Acyl-RNA:** 0.2 M DMSO, 24 hr					
+X Disulfide 2'-Conjugates	***Calculated [M-H]-***	**Observed [M-H]-**	***+Y Reduced Thiol 2'-Conjugates***	***Calculated [M-H]-***	**Observed [M-H]-**
+0	*5646.7955*	5651.3649	*+1*	*5762.8251*	5765.8115
+1	*5871.8237*	5876.4551	*+2*	*5882.8132*	5880.6209
+2	*6096.8520*	6093.2408	*+3*	*6000.8221*	5996.8334
+3	*6321.8802*	6322.3567	*+4*	*6118.8309*	6118.7102
			+6	*6354.8486*	6355.2327
**10-Acyl-RNA:** 0.4 M DMSO, 24 hr					
+X Disulfide 2'-Conjugates	***Calculated [M-H]-***	**Observed [M-H]-**	***+Y Reduced Thiol 2'-Conjugates***	***Calculated [M-H]-***	**Observed [M-H]-**
+0	*5646.7955*	5649.3138	*+1*	*5762.8251*	5765.6980
+1	*5871.8237*	5874.2843	*+2*	*5882.8132*	5883.1806
+2	*6096.8520*	6096.7414	*+3*	*6000.8221*	5997.6445
+3	*6321.8802*	6317.3180	*+4*	*6118.8309*	6112.5266
			+6	*6354.8486*	6356.4692
**10-Acyl-RNA:** 0.8 M DMSO, 24 hr					
+X Disulfide 2'-Conjugates	***Calculated [M-H]-***	**Observed [M-H]-**	***+Y Reduced Thiol 2'-Conjugates***	***Calculated [M-H]-***	**Observed [M-H]-**
+0	*5646.7955*	5646.8906	*+1*	*5762.8251*	5761.2131
+1	*5871.8237*	5871.3730	*+2*	*5882.8132*	5879.4856
+2	*6096.8520*	6094.3517	*+3*	*6000.8221*	5996.1775
+3	*6321.8802*	6322.0847	*+4*	*6118.8309*	6114.0536
+4	*6546.9084*	6548.1245	*+5*	*6226.9435*	6224.2902
			+6	*6354.8486*	6350.9533
			+8	*6590.8663*	6585.5155
**10-Acyl-RNA:** 0.8 M DMSO, 48 hr					
+X Disulfide 2'-Conjugates	***Calculated [M-H]-***	**Observed [M-H]-**	***+Y Reduced Thiol 2'-Conjugates***	***Calculated [M-H]-***	**Observed [M-H]-**
+0	*5646.7955*	5647.0055	*+1*	*5762.8251*	5762.8759
+1	*5871.8237*	5870.0757	*+2*	*5882.8132*	5883.0115
+2	*6096.8520*	6098.1165	*+3*	*6000.8221*	5992.5680
+3	*6321.8802*	6323.4820	*+4*	*6118.8309*	6112.9230
+4	*6546.9084*	6546.1894	*+5*	*6226.9435*	6227.9746
+5	*6771.9366*	6768.4893	*+6*	*6354.8486*	6357.4056
			+8	*6590.8663*	6591.0388
			+10	*6826.8840*	6830.0968
**10-Acyl-RNA:** 0.8 M DMSO, 72 hr					
+X Disulfide 2'-Conjugates	***Calculated [M-H]-***	**Observed [M-H]-**	***+Y Reduced Thiol 2'-Conjugates***	***Calculated [M-H]-***	**Observed [M-H]-**
+0	*5646.7955*	5646.3089	*+1*	*5762.8251*	5762.8573
+1	*5871.8237*	5875.5419	*+2*	*5882.8132*	5881.6320
+2	*6096.8520*	6094.1718	*+3*	*6000.8221*	6001.9013
+3	*6321.8802*	6320.5216	*+4*	*6118.8309*	6117.2198
+4	*6546.9084*	6547.8599	*+5*	*6226.9435*	6224.1981
+5	*6771.9366*	6770.9155	*+6*	*6354.8486*	6354.8982
			+8	*6590.8663*	6589.2820
			+10	*6826.8840*	6825.0904

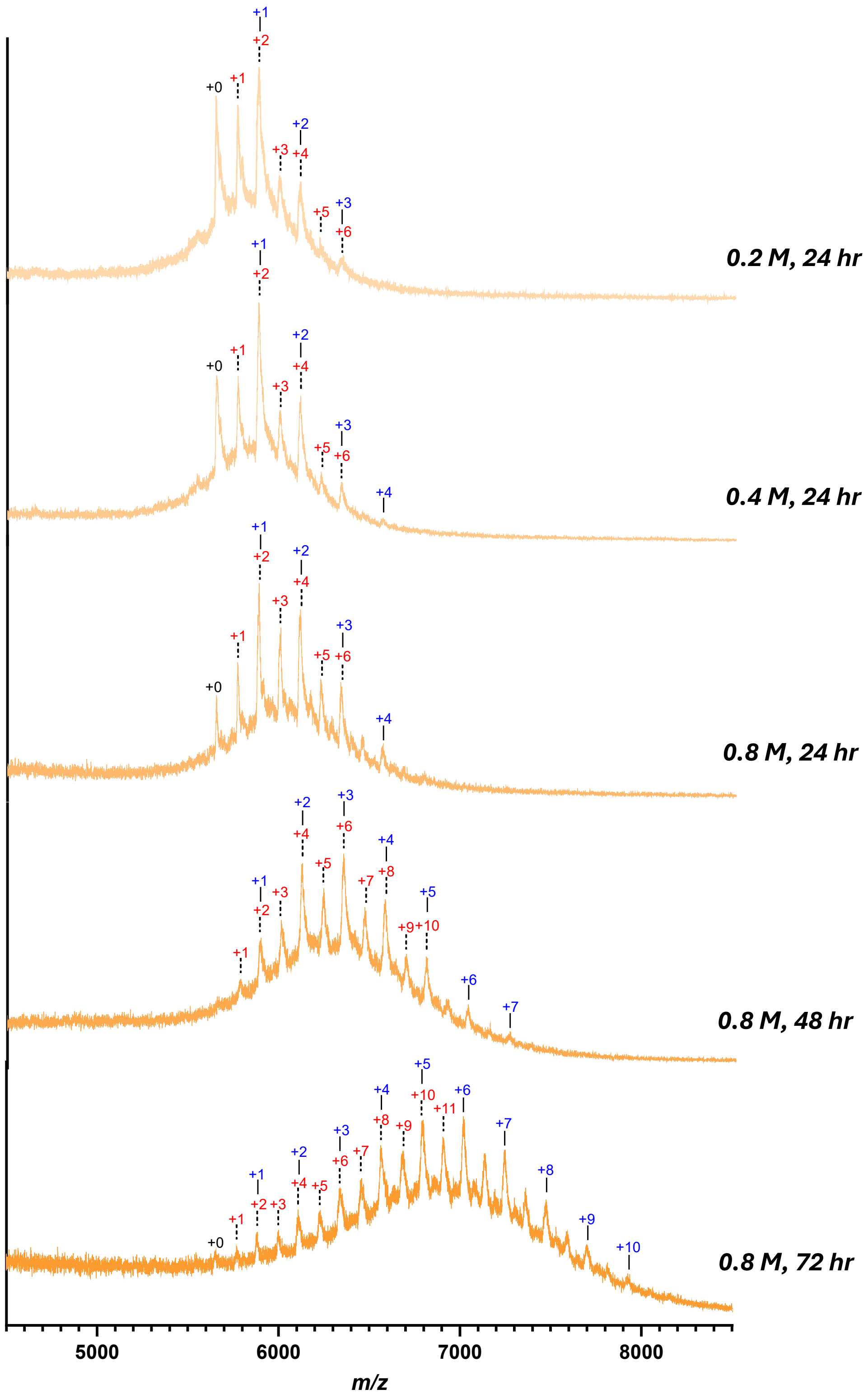

Figure S1E: MALDI-ToF mass spectra of RNA 2'-acylation reaction conditions to produce **13**-Acyl-RNA, with varying acylating reagent stock concentration in dry DMSO and duration of room temperature incubation. The number of intact disulfide conjugates (+X) are denoted in blue alongside the number of reduced thiol conjugates (+Y), denoted in red.

Table S5: Calculated and observed *m/z* values based on the above recorded MALDI-ToF spectra for **13**-Acyl-RNA models (Figure S1E). The number of intact disulfide conjugates (+X) are denoted in blue alongside the number of reduced thiol conjugates (+Y), denoted in red.

13-Acyl-RNA: 0.2 M DMSO, 24 hr					
+X Disulfide 2'-Conjugates	***Calculated [M-H]⁻***	**Observed [M-H]⁻**	***+Y Reduced Thiol 2'-Conjugates***	***Calculated [M-H]⁻***	**Observed [M-H]⁻**
+0	*5646.7955*	5646.4006	*+1*	*5764.8044*	5765.5585
+1	*5873.8030*	5874.0230	*+2*	*5882.8132*	5882.7484
+2	*6100.8105*	6101.1683	*+3*	*6000.8221*	5999.5281
+3	*6327.8180*	6328.2130	*+4*	*6118.8309*	6117.1910
			+5	*6236.8398*	6234.2316
			+6	*6354.8486*	6355.4178
13-Acyl-RNA: 0.4 M DMSO, 24 hr					
+X Disulfide 2'-Conjugates	***Calculated [M-H]⁻***	**Observed [M-H]⁻**	***+Y Reduced Thiol 2'-Conjugates***	***Calculated [M-H]⁻***	**Observed [M-H]⁻**
+0	*5646.7955*	5648.4447	*+1*	*5764.8044*	5764.3281
+1	*5873.8030*	5873.8052	*+2*	*5882.8132*	5881.7616
+2	*6100.8105*	6100.6955	*+3*	*6000.8221*	6001.2351
+3	*6327.8180*	6329.5493	*+4*	*6118.8309*	6119.1649
+4	*6554.8254*	6556.5237	*+5*	*6236.8398*	6235.9258
			+6	*6354.8486*	6355.1848
13-Acyl-RNA: 0.8 M DMSO, 24 hr					
+X Disulfide 2'-Conjugates	***Calculated [M-H]⁻***	**Observed [M-H]⁻**	***+Y Reduced Thiol 2'-Conjugates***	***Calculated [M-H]⁻***	**Observed [M-H]⁻**
+0	*5646.7955*	5647.7729	*+1*	*5764.8044*	5764.7139
+1	*5873.8030*	5875.5638	*+2*	*5882.8132*	5882.6669
+2	*6100.8105*	6102.0904	*+3*	*6000.8221*	6000.8663
+3	*6327.8180*	6327.5844	*+4*	*6118.8309*	6118.1829
+4	*6554.8254*	6557.0309	*+5*	*6236.8398*	6236.1819
			+6	*6354.8486*	6354.7499
13-Acyl-RNA: 0.8 M DMSO, 48 hr					
+X Disulfide 2'-Conjugates	***Calculated [M-H]⁻***	**Observed [M-H]⁻**	***+Y Reduced Thiol 2'-Conjugates***	***Calculated [M-H]⁻***	**Observed [M-H]⁻**
+1	*5873.8030*	5878.7925	*+1*	*5764.8044*	5769.3055
+2	*6100.8105*	6106.4950	*+2*	*5882.8132*	5882.5072
+3	*6327.8180*	6330.2836	*+3*	*6000.8221*	6004.1330
+4	*6554.8254*	6559.3486	*+4*	*6118.8309*	6118.7535
+5	*6781.8329*	6784.8094	*+5*	*6236.8398*	6236.8041
+6	*7008.8404*	7015.6309	*+6*	*6354.8486*	6350.1147
+7	*7235.8478*	7239.8560	*+7*	*6472.8575*	6468.5595
			+8	*6590.8663*	6591.4491
			+9	*6708.8752*	6707.9934
			+10	*6826.8840*	6825.0482
13-Acyl-RNA: 0.8 M DMSO, 72 hr					
+X Disulfide 2'-Conjugates	***Calculated [M-H]⁻***	**Observed [M-H]⁻**	***+Y Reduced Thiol 2'-Conjugates***	***Calculated [M-H]⁻***	**Observed [M-H]⁻**
+1	*5873.8030*	5875.8327	*+1*	*5764.8044*	5768.4250
+2	*6100.8105*	6102.1360	*+2*	*5882.8132*	5883.8735
+3	*6327.8180*	6328.4404	*+3*	*6000.8221*	6001.0245
+4	*6554.8254*	6557.2895	*+4*	*6118.8309*	6120.3302
+5	*6781.8329*	6781.0803	*+5*	*6236.8398*	6234.8687
+6	*7008.8404*	7009.9932	*+6*	*6354.8486*	6351.3549
+7	*7235.8478*	7235.4284	*+7*	*6472.8575*	6469.6714
+8	*7462.8553*	7459.7596	*+8*	*6590.8663*	6589.9825
+9	*7689.8628*	7690.2363	*+9*	*6708.8752*	6709.2705
+10	*7916.8703*	7916.1189	*+10*	*6826.8840*	6826.6303
			+11	*6944.8929*	6946.0877

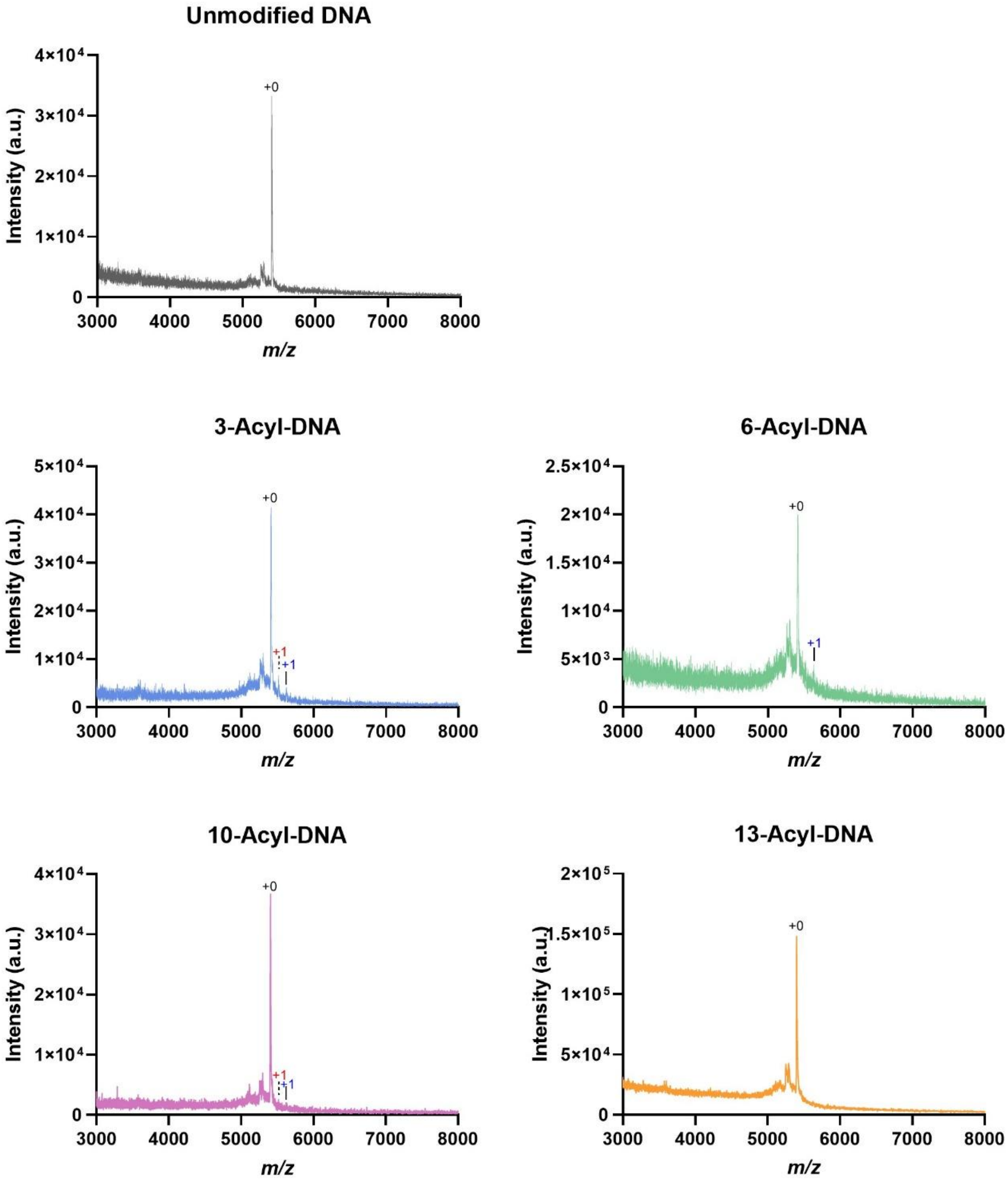

Figure S2A: MALDI-ToF mass spectra of unmodified and acylated **DNA1** models utilising **3**, **6**, **10** & **13**. The number of intact disulfide conjugates (+X) are denoted in blue alongside the number of reduced thiol conjugates (+Y), denoted in red.

Table S6: Calculated and observed *m/z* values based on the above recorded MALDI-ToF spectra for Acyl-DNA models (Figure S2A). The number of intact disulfide conjugates (+X) are denoted in blue alongside the number of reduced thiol conjugates (+Y), denoted in red.

Unmodified DNA					
+X Disulfide 2'-Conjugates	***Calculated [M-H]⁻***	**Observed [M-H]⁻**	***+Y Reduced Thiol 2'-Conjugates***	***Calculated [M-H]⁻***	**Observed [M-H]⁻**
+0	*5400.9340*	5401.3915			
**3-Acyl-DNA**					
+X Disulfide 2'-Conjugates	***Calculated [M-H]⁻***	**Observed [M-H]⁻**	***+Y Reduced Thiol 2'-Conjugates***	***Calculated [M-H]⁻***	**Observed [M-H]⁻**
+0	*5400.9340*	5410.6452	*+1*	*5502.9480*	5510.9185
+1	*5611.9466*	5618.9164			
**6-Acyl-DNA**					
+X Disulfide 2'-Conjugates	***Calculated [M-H]⁻***	**Observed [M-H]⁻**	***+Y Reduced Thiol 2'-Conjugates***	***Calculated [M-H]⁻***	**Observed [M-H]⁻**
+0	*5400.9340*	5407.9565			
+1	*5613.9259*	5623.7047			
**10-Acyl-DNA**					
+X Disulfide 2'-Conjugates	***Calculated [M-H]⁻***	**Observed [M-H]⁻**	***+Y Reduced Thiol 2'-Conjugates***	***Calculated [M-H]⁻***	**Observed [M-H]⁻**
+0	*5400.9340*	5406.4773	*+1*	*5516.9636*	5518.6978
+1	*5625.9622*	5631.7708			
**13-Acyl-DNA**					
+X Disulfide 2'-Conjugates	***Calculated [M-H]⁻***	**Observed [M-H]⁻**	***+Y Reduced Thiol 2'-Conjugates***	***Calculated [M-H]⁻***	**Observed [M-H]⁻**
+0	*5400.9340*	5400.6911			

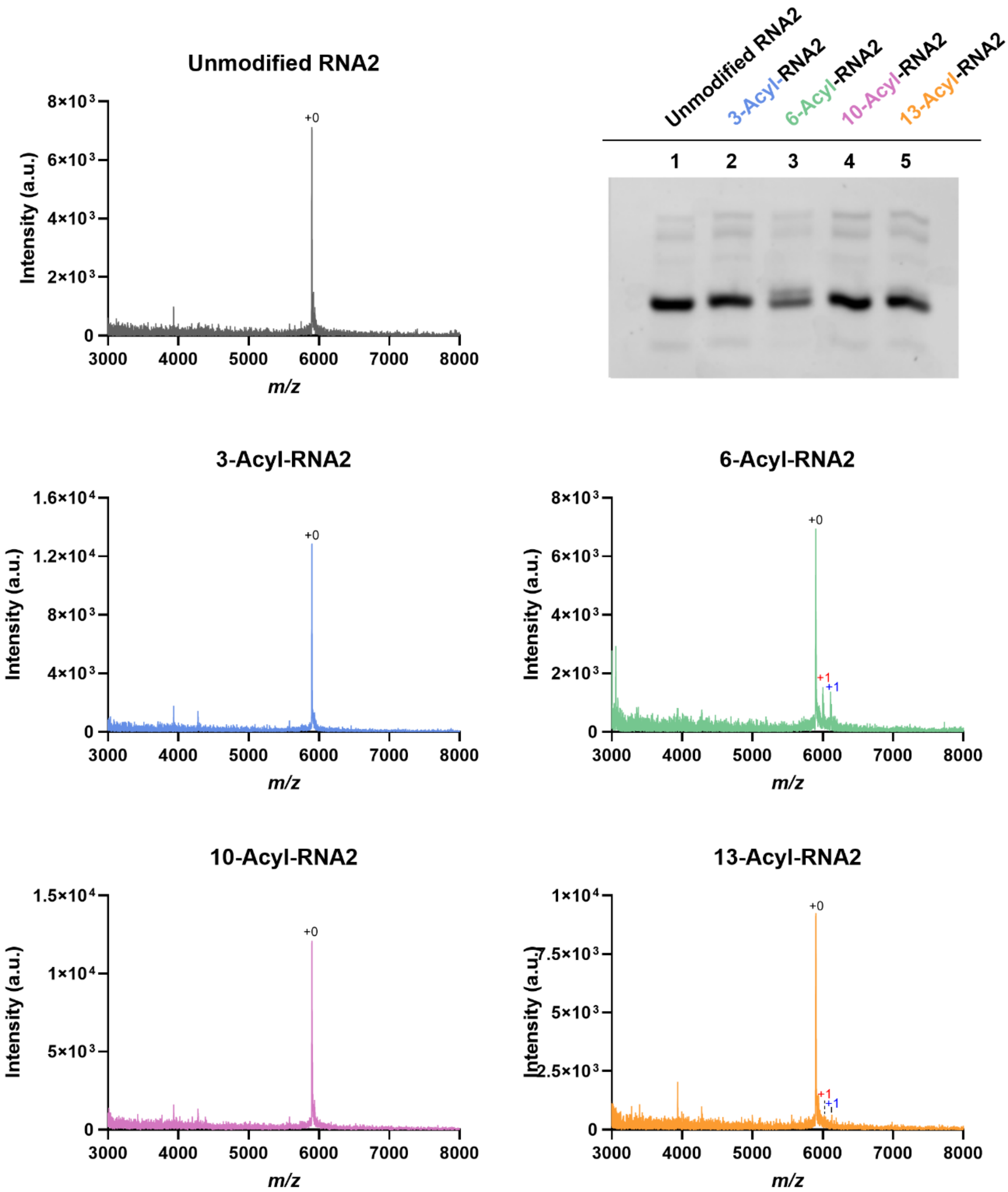

Figure S2B: MALDI-ToF mass spectra and urea-PAGE analysis of unmodified and acylated **RNA2** models utilising **3**, **6**, **10** & **13**. The number of intact disulfide conjugates (+X) are denoted in blue alongside the number of reduced thiol conjugates (+Y), denoted in red.

Table S7: Calculated and observed *m/z* values based on the above recorded MALDI-ToF spectra for unmodified and Acyl-**RNA2** models (Figure S2B). The number of intact disulfide conjugates (+X) are denoted in blue alongside the number of reduced thiol conjugates (+Y), denoted in red.

Unmodified RNA2					
+X Disulfide 2'-Conjugates	***Calculated [M-H]$^-$***	**Observed [M-H]$^-$**	***+Y Reduced Thiol 2'-Conjugates***	***Calculated [M-H]$^-$***	**Observed [M-H]$^-$**
+0	*5899.0772*	5899.4427			
**3-Acyl-RNA2**					
+X Disulfide 2'-Conjugates	***Calculated [M-H]$^-$***	**Observed [M-H]$^-$**	***+Y Reduced Thiol 2'-Conjugates***	***Calculated [M-H]$^-$***	**Observed [M-H]$^-$**
+0	*5899.0772*	5900.0287			
**6-Acyl-RNA2**					
+X Disulfide 2'-Conjugates	***Calculated [M-H]$^-$***	**Observed [M-H]$^-$**	***+Y Reduced Thiol 2'-Conjugates***	***Calculated [M-H]$^-$***	**Observed [M-H]$^-$**
+0	*5899.0772*	5900.7888	*+1*	*6003.0704*	6004.9946
+1	*6112.0691*	6111.3967			
**10-Acyl-RNA2**					
+X Disulfide 2'-Conjugates	***Calculated [M-H]$^-$***	**Observed [M-H]$^-$**	***+Y Reduced Thiol 2'-Conjugates***	***Calculated [M-H]$^-$***	**Observed [M-H]$^-$**
+0	*5899.0772*	5900.1772			
**13-Acyl-RNA2**					
+X Disulfide 2'-Conjugates	***Calculated [M-H]$^-$***	**Observed [M-H]$^-$**	***+Y Reduced Thiol 2'-Conjugates***	***Calculated [M-H]$^-$***	**Observed [M-H]$^-$**
+0	*5899.0772*	5901.7512	*+1*	*6017.0861*	6020.7589
+1	*6126.0847*	6130.6991			

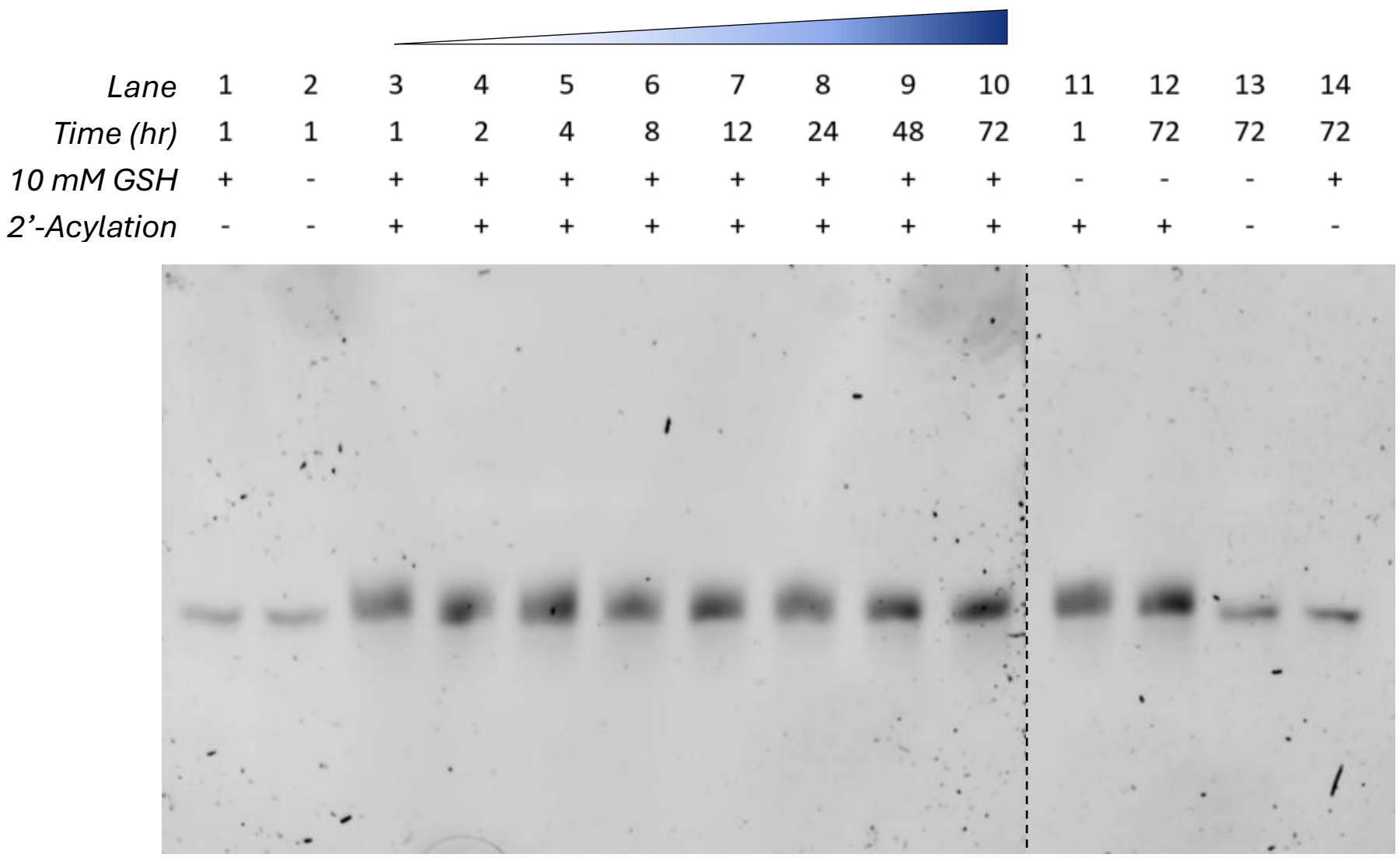

Figure S3A: Urea-PAGE analysis of unmodified and **3**-Acyl-RNA samples incubated in the presence or absence of 10 mM reduced glutathione at various time points.

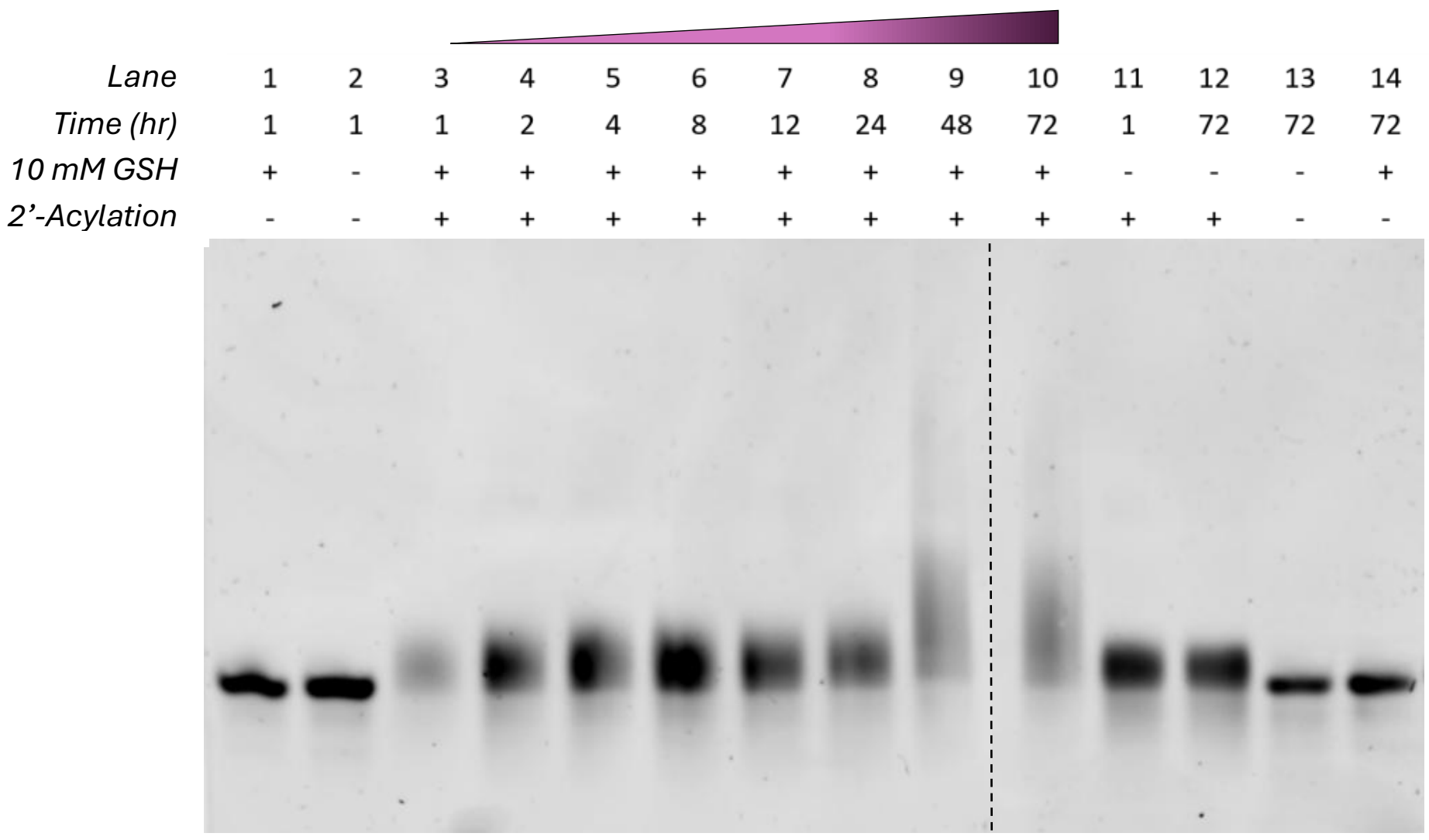

Figure S3B: Urea-PAGE analysis of unmodified and **10**-Acyl-RNA samples incubated in the presence or absence of 10 mM reduced glutathione at various time points.

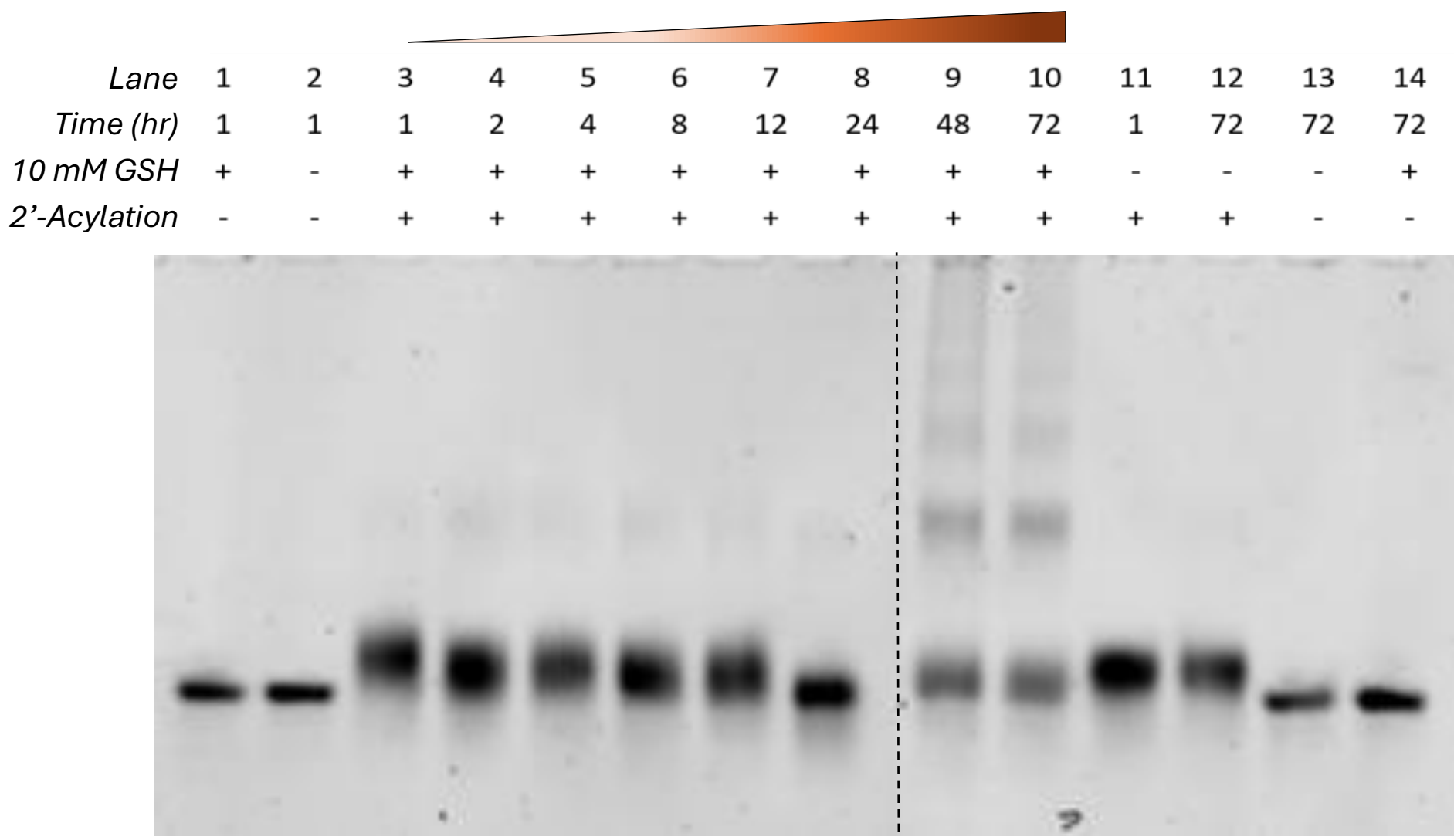

Figure S3C: Urea-PAGE analysis of unmodified and **13**-Acyl-RNA samples incubated in the presence or absence of 10 mM reduced glutathione at various time points.

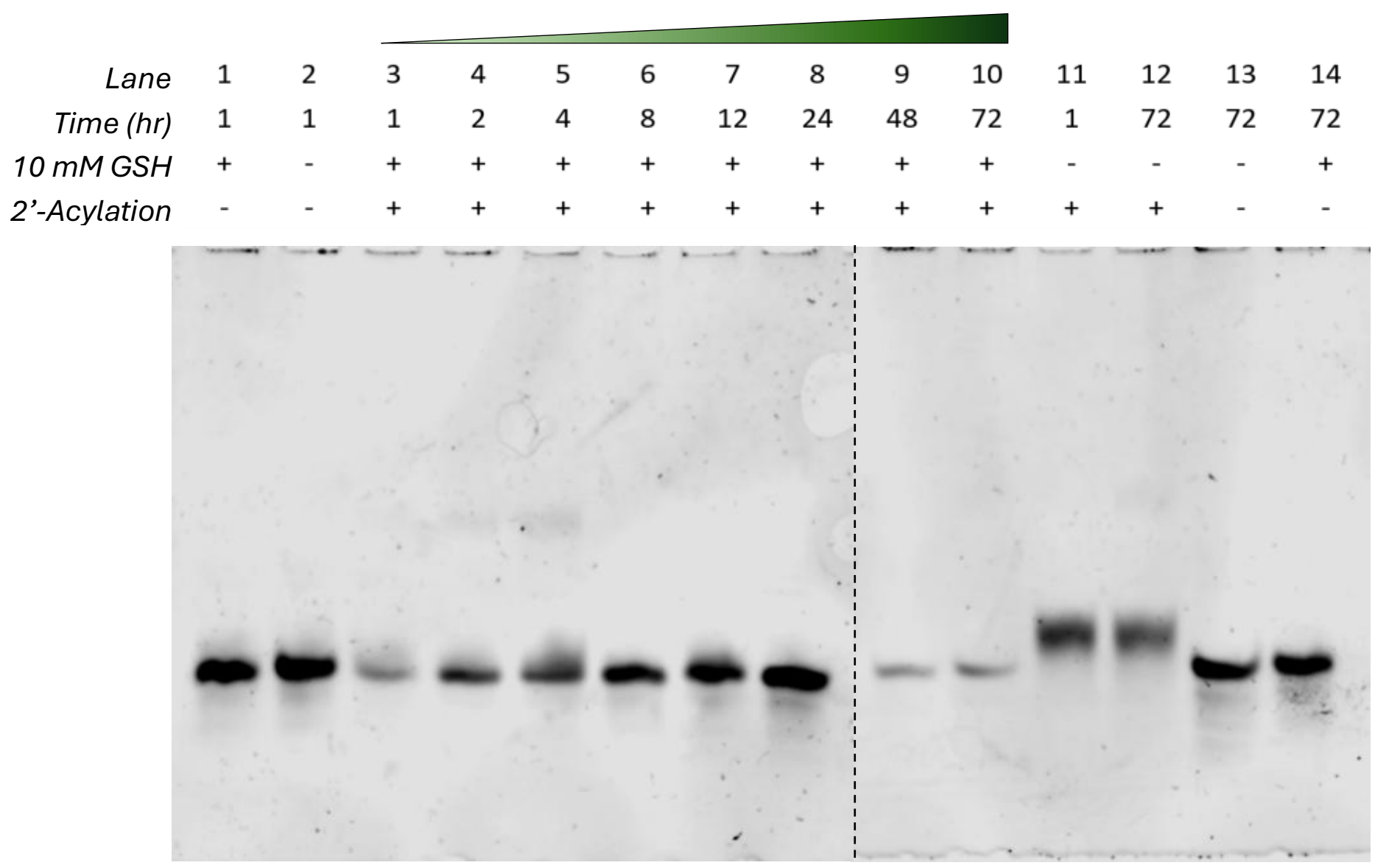

Figure S3D: Urea-PAGE analysis of unmodified and **6**-Acyl-RNA samples incubated in the presence or absence of 10 mM reduced glutathione at various time points.

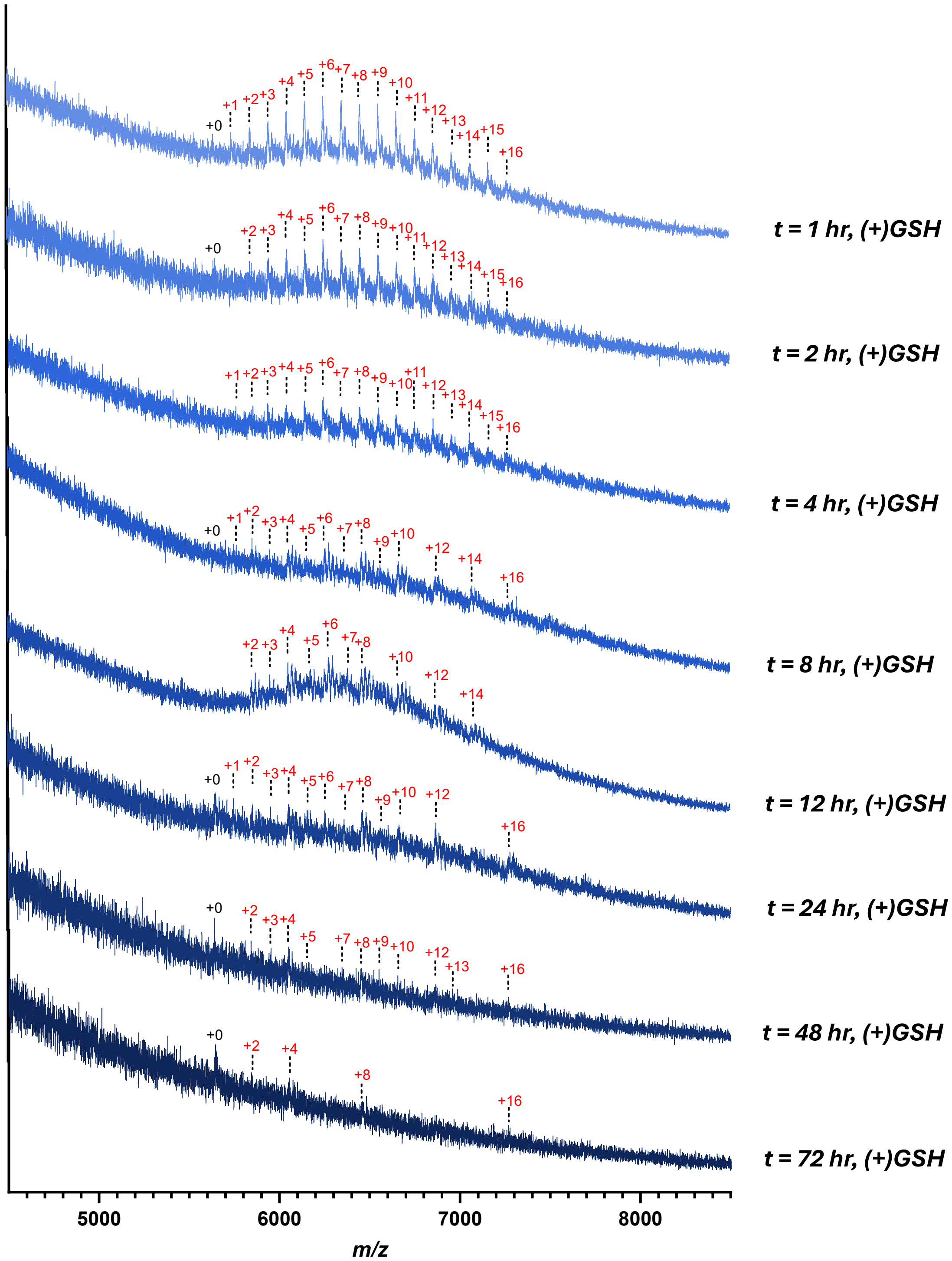

Figure S4A: MALDI-ToF mass spectra of **3**-Acyl-RNA samples incubated in the presence of 10 mM reduced glutathione at various time points, with number of reduced thiol conjugates (+Y) denoted in red.

Table S8: Calculated and observed *m/z* values based on the above recorded MALDI-ToF spectra for **3**-Acyl-RNA samples in the presence of 10 mM reduced glutathione at various timepoints (Figure S4A). The number of intact disulfide conjugates (+X) are denoted in blue alongside the number of reduced thiol conjugates (+Y), denoted in red.

3-Acyl-RNA						
t = 1 hr, (+)GSH	***+X Disulfide 2'-Conjugates***	***Calculated $[M-H]^-$***	**Observed $[M-H]^-$**	***+Y Reduced Thiol 2'-Conjugates***	***Calculated $[M-H]^-$***	**Observed $[M-H]^-$**
	+0	*5646.7955*	5644.8117	*+1*	*5748.8095*	5747.0480
				+2	*5850.8234*	5849.1609
				+3	*5952.8374*	5951.1982
				+4	*6054.8513*	6051.6889
				+5	*6156.8652*	6154.6073
				+6	*6258.8792*	6257.6379
				+7	*6360.8931*	6357.0671
				+8	*6462.9070*	6459.3439
				+9	*6564.9210*	6561.9665
				+10	*6666.9349*	6663.9118
				+11	*6768.9488*	6765.1178
				+12	*6870.9628*	6867.0472
				+13	*6972.9767*	6968.4365
				+14	*7074.9906*	7072.3005
				+15	*7177.0046*	7173.4164
				+16	*7279.0185*	7277.3237
t = 2 hr, (+)GSH	***+X Disulfide 2'-Conjugates***	***Calculated $[M-H]^-$***	**Observed $[M-H]^-$**	***+Y Reduced Thiol 2'-Conjugates***	***Calculated $[M-H]^-$***	**Observed $[M-H]^-$**
	+0	*5646.7955*	5644.0049	*+2*	*5850.8234*	5847.0008
				+3	*5952.8374*	5951.3519
				+4	*6054.8513*	6052.1608
				+5	*6156.8652*	6156.2807
				+6	*6258.8792*	6255.5240
				+7	*6360.8931*	6358.6038
				+8	*6462.9070*	6460.4326
				+9	*6564.9210*	6561.6624
				+10	*6666.9349*	6663.6441
				+11	*6768.9488*	6764.3743
				+12	*6870.9628*	6869.7942
				+13	*6972.9767*	6969.3998
				+14	*7074.9906*	7076.9280
				+15	*7177.0046*	7173.2904
				+16	*7279.0185*	7276.0587
t = 4 hr, (+)GSH	***+X Disulfide 2'-Conjugates***	***Calculated $[M-H]^-$***	**Observed $[M-H]^-$**	***+Y Reduced Thiol 2'-Conjugates***	***Calculated $[M-H]^-$***	**Observed $[M-H]^-$**
				+1	*5748.8095*	5750.1649
				+2	*5850.8234*	5854.2703
				+3	*5952.8374*	5953.1585
				+4	*6054.8513*	6054.1879
				+5	*6156.8652*	6154.4530
				+6	*6258.8792*	6254.3932
				+7	*6360.8931*	6361.4646
				+8	*6462.9070*	6464.8762
				+9	*6564.9210*	6566.8945
				+10	*6666.9349*	6666.8888
				+11	*6768.9488*	6767.9090
				+12	*6870.9628*	6867.3570
				+13	*6972.9767*	6966.0512
				+14	*7074.9906*	7068.2464
				+15	*7177.0046*	7176.4259
				+16	*7279.0185*	7275.5053
t = 8 hr, (+)GSH	***+X Disulfide 2'-Conjugates***	***Calculated $[M-H]^-$***	**Observed $[M-H]^-$**	***+Y Reduced Thiol 2'-Conjugates***	***Calculated $[M-H]^-$***	**Observed $[M-H]^-$**
	+0	*5646.7955*	5647.3910	*+1*	*5748.8095*	5748.8936
				+2	*5850.8234*	5852.2379
				+3	*5952.8374*	5952.5882
				+4	*6054.8513*	6053.5922
				+5	*6156.8652*	6155.8003
				+6	*6258.8792*	6258.0860
				+7	*6360.8931*	6361.7870
				+8	*6462.9070*	6462.9340
				+9	*6564.9210*	6565.7062
				+10	*6666.9349*	6665.6304
				+12	*6870.9628*	6868.2264
				+14	*7074.9906*	7073.3832
				+16	*7279.0185*	7277.4984

t = 12 hr, (+)GSH	***+X Disulfide 2'-Conjugates***	***Calculated $[M-H]^-$***	**Observed $[M-H]^-$**	***+Y Reduced Thiol 2'-Conjugates***	***Calculated $[M-H]^-$***	**Observed $[M-H]^-$**
				+2	*5850.8234*	5851.4634
				+3	*5952.8374*	5954.3156
				+4	*6054.8513*	6053.8051
				+5	*6156.8652*	6156.1286
				+6	*6258.8792*	6257.7437
				+7	*6360.8931*	6359.2867
				+8	*6462.9070*	6463.4846
				+10	*6666.9349*	6666.9295
				+12	*6870.9628*	6870.7081
				+14	*7074.9906*	7077.5560
t = 24 hr, (+)GSH	***+X Disulfide 2'-Conjugates***	***Calculated $[M-H]^-$***	**Observed $[M-H]^-$**	***+Y Reduced Thiol 2'-Conjugates***	***Calculated $[M-H]^-$***	**Observed $[M-H]^-$**
	+0	*5646.7955*	5645.6101	*+1*	*5748.8095*	5750.4625
				+2	*5850.8234*	5852.6091
				+3	*5952.8374*	5957.3908
				+4	*6054.8513*	6056.6673
				+5	*6156.8652*	6158.1854
				+6	*6258.8792*	6256.4435
				+7	*6360.8931*	6363.7408
				+8	*6462.9070*	6461.3659
				+9	*6564.9210*	6563.8802
				+10	*6666.9349*	6665.1433
				+12	*6870.9628*	6872.0182
				+16	*7279.0185*	7282.2079
t = 48 hr, (+)GSH	***+X Disulfide 2'-Conjugates***	***Calculated $[M-H]^-$***	**Observed $[M-H]^-$**	***+Y Reduced Thiol 2'-Conjugates***	***Calculated $[M-H]^-$***	**Observed $[M-H]^-$**
	+0	*5646.7955*	5645.0277	*+2*	*5850.8234*	5846.1946
				+3	*5952.8374*	5956.4076
				+4	*6054.8513*	6054.2176
				+5	*6156.8652*	6154.6728
				+7	*6360.8931*	6358.8948
				+8	*6462.9070*	6459.6595
				+9	*6564.9210*	6565.0989
				+10	*6666.9349*	6667.9757
				+12	*6870.9628*	6868.7692
				+13	*6972.9767*	6970.0269
				+16	*7279.0185*	7276.1228
t = 72 hr, (+)GSH	***+X Disulfide 2'-Conjugates***	***Calculated $[M-H]^-$***	**Observed $[M-H]^-$**	***+Y Reduced Thiol 2'-Conjugates***	***Calculated $[M-H]^-$***	**Observed $[M-H]^-$**
	+0	*5646.7955*	5645.8309	*+2*	*5850.8234*	5851.9398
				+4	*6054.8513*	6053.7537
				+8	*6462.9070*	6461.1043
				+16	*7279.0185*	7277.1240

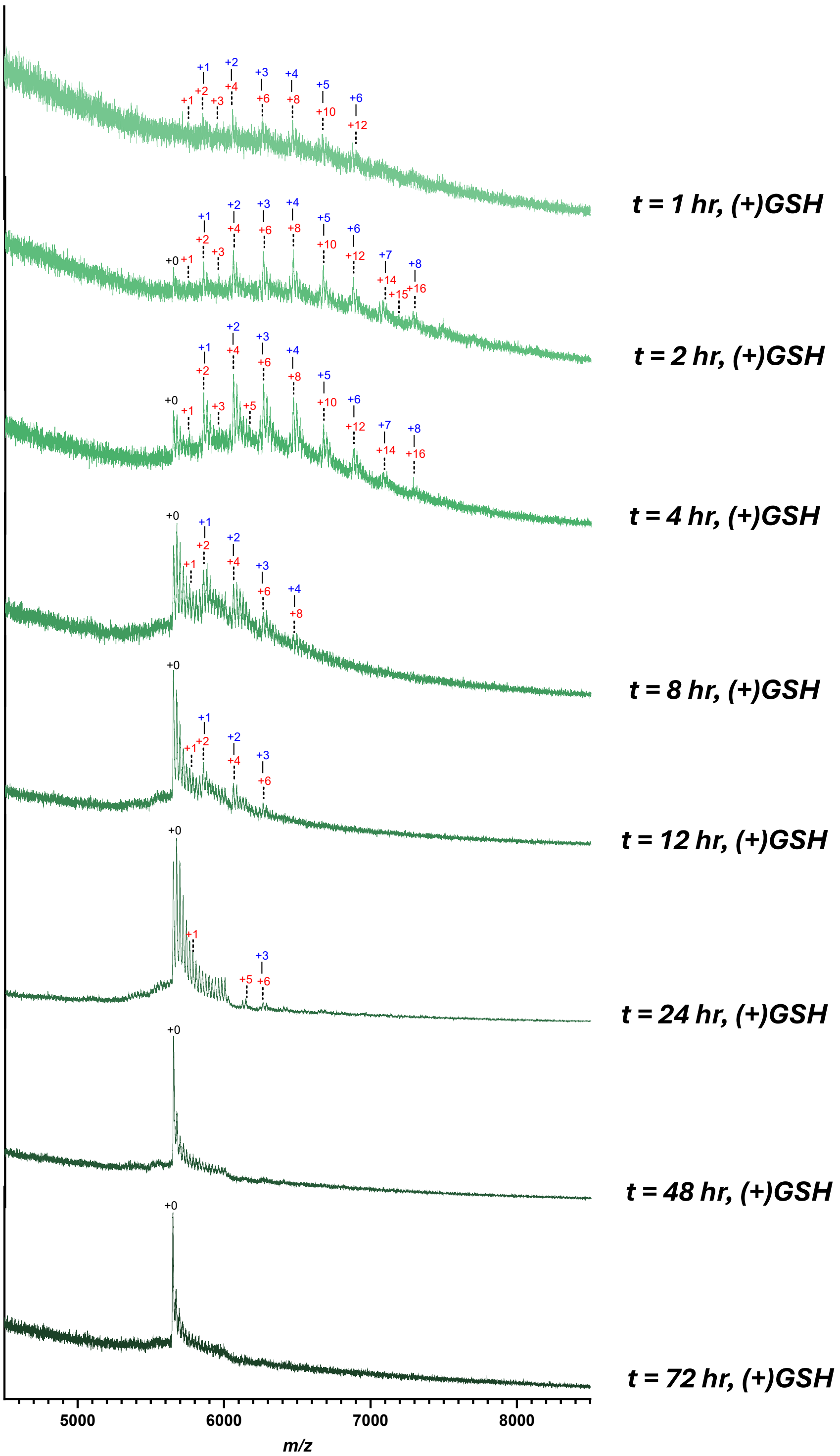

Figure S4B:MALDI-ToF mass spectra of **6**-Acyl-RNA samples incubated in the presence of 10 mM reduced glutathione at various time points. The number of intact disulfide conjugates (+X) are denoted in blue alongside the number of reduced thiol conjugates (+Y), denoted in red.

Table S9: Calculated and observed *m/z* values based on the above recorded MALDI-ToF spectra for **6**-Acyl-RNA samples in the presence of 10 mM reduced glutathione at various timepoints (Figure S4B). The number of intact disulfide conjugates (+X) are denoted in blue alongside the number of reduced thiol conjugates (+Y), denoted in red.

6-Acyl-RNA						
t = 1 hr, (+)GSH	***+X Disulfide 2'-Conjugates***	***Calculated [M-H]$^-$***	**Observed [M-H]$^-$**	***+Y Reduced Thiol 2'-Conjugates***	***Calculated [M-H]$^-$***	**Observed [M-H]$^-$**
	+1	*5859.7874*	5856.8359	*+1*	*5750.7887*	5753.1865
	+2	*6072.7792*	6070.1600	*+2*	*5854.7819*	5856.1907
	+3	*6285.7710*	6286.9406	*+3*	*5958.7751*	5958.4994
	+4	*6498.7628*	6495.6637	*+4*	*6062.7683*	6061.2270
	+5	*6711.7546*	6712.8155	*+6*	*6270.7547*	6266.4668
	+6	*6924.7465*	6924.2582	*+8*	*6478.7411*	6477.0592
				+10	*6686.7275*	6680.4045
				+12	*6894.7139*	6896.8043
t = 2 hr, (+)GSH	***+X Disulfide 2'-Conjugates***	***Calculated [M-H]$^-$***	**Observed [M-H]$^-$**	***+Y Reduced Thiol 2'-Conjugates***	***Calculated [M-H]$^-$***	**Observed [M-H]$^-$**
	+0	*5646.7955*	5646.8664	*+1*	*5750.7887*	5753.4895
	+1	*5859.7874*	5856.5238	*+2*	*5854.7819*	5855.2746
	+2	*6072.7792*	6071.9178	*+3*	*5958.7751*	5957.4785
	+3	*6285.7710*	6285.7331	*+4*	*6062.7683*	6061.3636
	+4	*6498.7628*	6494.9575	*+6*	*6270.7547*	6376.9292
	+5	*6711.7546*	6712.4720	*+8*	*6478.7411*	6478.7409
	+6	*6924.7465*	6923.6784	*+10*	*6686.7275*	6689.8919
	+7	*7137.7383*	7136.1261	*+12*	*6894.7139*	6897.5923
	+8	*7350.7301*	7348.1101	*+14*	*7102.7004*	7105.6856
				+15	*7206.6936*	7206.3820
				+16	*7310.6868*	7308.5334
t = 4 hr, (+)GSH	***+X Disulfide 2'-Conjugates***	***Calculated [M-H]$^-$***	**Observed [M-H]$^-$**	***+Y Reduced Thiol 2'-Conjugates***	***Calculated [M-H]$^-$***	**Observed [M-H]$^-$**
	+0	*5646.7955*	5646.2914	*+1*	*5750.7887*	5751.9879
	+1	*5859.7874*	5859.3897	*+2*	*5854.7819*	5855.4584
	+2	*6072.7792*	6077.8435	*+3*	*5958.7751*	5959.4972
	+3	*6285.7710*	6285.4187	*+4*	*6062.7683*	6060.0375
	+4	*6498.7628*	6497.5283	*+5*	*6166.7615*	6163.5753
	+5	*6711.7546*	6709.6179	*+6*	*6270.7547*	6266.8945
	+6	*6924.7465*	6922.2756	*+8*	*6478.7411*	6475.4723
	+7	*7137.7383*	7137.8151	*+10*	*6686.7275*	6688.5438
	+8	*7350.7301*	7351.4710	*+12*	*6894.7139*	6896.5896
				+14	*7102.7004*	7102.8904
				+16	*7310.6868*	7311.0457
t = 8 hr, (+)GSH	***+X Disulfide 2'-Conjugates***	***Calculated [M-H]$^-$***	**Observed [M-H]$^-$**	***+Y Reduced Thiol 2'-Conjugates***	***Calculated [M-H]$^-$***	**Observed [M-H]$^-$**
	+0	*5646.7955*	5649.8613	*+1*	*5750.7887*	5752.3641
	+1	*5859.7874*	5858.6361	*+2*	*5854.7819*	5855.3615
	+2	*6072.7792*	6073.4883	*+4*	*6062.7683*	6061.0531
	+3	*6285.7710*	6284.3634	*+6*	*6270.7547*	6265.6842
	+4	*6498.7628*	6497.1867	*+8*	*6478.7411*	6475.9089
t = 12 hr, (+)GSH	***+X Disulfide 2'-Conjugates***	***Calculated [M-H]$^-$***	**Observed [M-H]$^-$**	***+Y Reduced Thiol 2'-Conjugates***	***Calculated [M-H]$^-$***	**Observed [M-H]$^-$**
	+0	*5646.7955*	5649.8347	*+1*	*5750.7887*	5748.1352
	+1	*5859.7874*	5855.6006	*+2*	*5854.7819*	5854.2897
	+2	*6072.7792*	6073.4984	*+4*	*6062.7683*	6062.6186
	+3	*6285.7710*	6284.9128	*+6*	*6270.7547*	6373.5255
t = 24 hr, (+)GSH	***+X Disulfide 2'-Conjugates***	***Calculated [M-H]$^-$***	**Observed [M-H]$^-$**	***+Y Reduced Thiol 2'-Conjugates***	***Calculated [M-H]$^-$***	**Observed [M-H]$^-$**
	+0	*5646.7955*	5649.6792	*+1*	*5750.7887*	5753.4531
	+3	*6285.7710*	6285.5593	*+5*	*6166.7615*	6168.6623
				+6	*6270.7547*	6269.3749
t = 48 hr, (+)GSH	***+X Disulfide 2'-Conjugates***	***Calculated [M-H]$^-$***	**Observed [M-H]$^-$**	***+Y Reduced Thiol 2'-Conjugates***	***Calculated [M-H]$^-$***	**Observed [M-H]$^-$**
	+0	*5646.7955*	5650.9886			
t = 72 hr, (+)GSH	***+X Disulfide 2'-Conjugates***	***Calculated [M-H]$^-$***	**Observed [M-H]$^-$**	***+Y Reduced Thiol 2'-Conjugates***	***Calculated [M-H]$^-$***	**Observed [M-H]$^-$**
	+0	*5646.7955*	5651.1217			

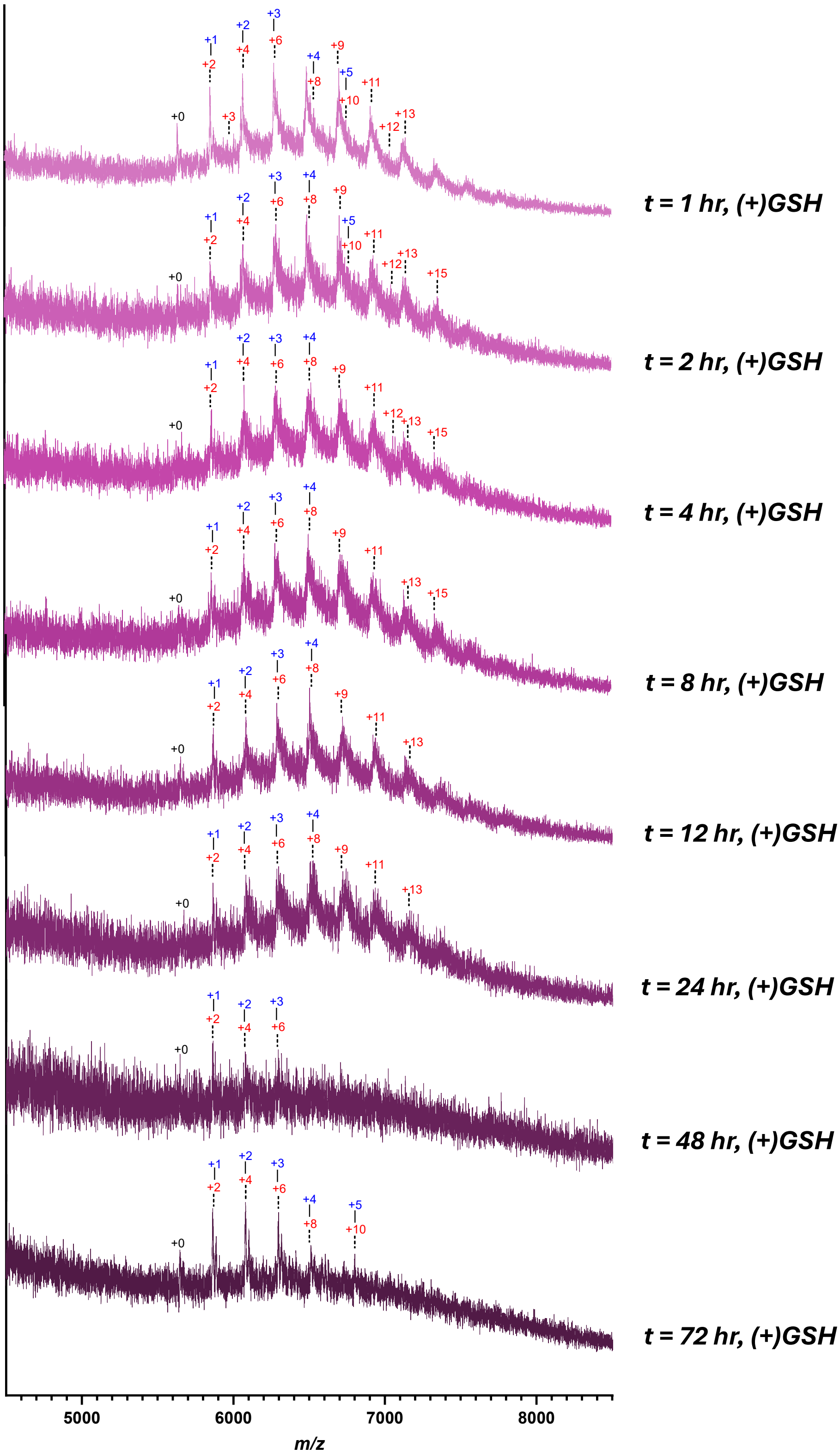

Figure S4C: MALDI-ToF mass spectra of **10**-Acyl-RNA samples incubated in the presence of 10 mM reduced glutathione at various time points. The number of intact disulfide conjugates (+X) are denoted in blue alongside the number of reduced thiol conjugates (+Y), denoted in red.

Table S10: Calculated and observed *m/z* values based on the above recorded MALDI-ToF spectra for **10**-Acyl-RNA samples in the presence of 10 mM reduced glutathione at various timepoints (Figure S4C). The number of intact disulfide conjugates (+X) are denoted in blue alongside the number of reduced thiol conjugates (+Y), denoted in red.

10-Acyl-RNA						
***t* = 1 hr, (+)GSH**	***+X Disulfide 2'-Conjugates***	***Calculated $[M-H]^-$***	**Observed $[M-H]^-$**	***+Y Reduced Thiol 2'-Conjugates***	***Calculated $[M-H]^-$***	**Observed $[M-H]^-$**
	+0	*5646.7955*	5643.1670	*+2*	*5882.8132*	5881.5340
	+1	*5871.8237*	5875.5837	*+3*	*6000.8221*	6006.1545
	+2	*6096.8520*	6093.7633	*+4*	*6118.8309*	6117.1679
	+3	*6321.8802*	6321.3282	*+6*	*6354.8486*	6356.8987
	+4	*6546.9084*	6544.3711	*+8*	*6590.8663*	6595.1192
	+5	*6771.9366*	6769.8796	*+9*	*6708.8752*	6706.8264
				+10	*6826.8840*	6822.7820
				+11	*6944.8929*	6945.6977
				+12	*7062.9018*	7063.5338
				+13	*7180.9106*	7175.3635
***t* = 2 hr, (+)GSH**	***+X Disulfide 2'-Conjugates***	***Calculated $[M-H]^-$***	**Observed $[M-H]^-$**	***+Y Reduced Thiol 2'-Conjugates***	***Calculated $[M-H]^-$***	**Observed $[M-H]^-$**
	+0	*5646.7955*	5646.5632	*+2*	*5882.8132*	5879.8769
	+1	*5871.8237*	5868.8106	*+4*	*6118.8309*	6114.5769
	+2	*6096.8520*	6097.7848	*+6*	*6354.8486*	6353.0447
	+3	*6321.8802*	6323.1793	*+8*	*6590.8663*	6595.8533
	+4	*6546.9084*	6544.5285	*+9*	*6708.8752*	6708.8180
	+5	*6771.9366*	6767.2664	*+10*	*6826.8840*	6827.0623
				+11	*6944.8929*	6944.2058
				+12	*7062.9018*	7062.1154
				+13	*7180.9106*	7174.1121
				+15	*7416.9283*	7413.2233
***t* = 4 hr, (+)GSH**	***+X Disulfide 2'-Conjugates***	***Calculated $[M-H]^-$***	**Observed $[M-H]^-$**	***+Y Reduced Thiol 2'-Conjugates***	***Calculated $[M-H]^-$***	**Observed $[M-H]^-$**
	+0	*5646.7955*	5644.4740	*+2*	*5882.8132*	5883.4787
	+1	*5871.8237*	5867.3118	*+4*	*6118.8309*	6117.0790
	+2	*6096.8520*	6098.5199	*+6*	*6354.8486*	6352.6864
	+3	*6321.8802*	6322.6229	*+8*	*6590.8663*	6593.0417
	+4	*6546.9084*	6544.3907	*+9*	*6708.8752*	6706.7911
				+11	*6944.8929*	6945.5682
				+12	*7062.9018*	7062.4643
				+13	*7180.9106*	7181.5360
				+15	*7416.9283*	7419.1072
***t* = 8 hr, (+)GSH**	***+X Disulfide 2'-Conjugates***	***Calculated $[M-H]^-$***	**Observed $[M-H]^-$**	***+Y Reduced Thiol 2'-Conjugates***	***Calculated $[M-H]^-$***	**Observed $[M-H]^-$**
	+0	*5646.7955*	5647.0031	*+2*	*5882.8132*	5881.6131
	+1	*5871.8237*	5870.9349	*+4*	*6118.8309*	6114.2377
	+2	*6096.8520*	6094.1199	*+6*	*6354.8486*	6351.6812
	+3	*6321.8802*	6322.4859	*+8*	*6590.8663*	6591.5937
	+4	*6546.9084*	6543.3301	*+9*	*6708.8752*	6707.3491
				+11	*6944.8929*	6943.1727
				+13	*7180.9106*	7180.2731
				+15	*7416.9283*	7420.4386
***t* = 12 hr, (+)GSH**	***+X Disulfide 2'-Conjugates***	***Calculated $[M-H]^-$***	**Observed $[M-H]^-$**	***+Y Reduced Thiol 2'-Conjugates***	***Calculated $[M-H]^-$***	**Observed $[M-H]^-$**
	+0	*5646.7955*	5648.5172	*+2*	*5882.8132*	5880.8125
	+1	*5871.8237*	5870.1565	*+4*	*6118.8309*	6114.6947
	+2	*6096.8520*	6095.2051	*+6*	*6354.8486*	6353.5887
	+3	*6321.8802*	6321.4587	*+8*	*6590.8663*	6589.9265
	+4	*6546.9084*	6548.0595	*+9*	*6708.8752*	6708.4587
				+11	*6944.8929*	6939.8821
				+13	*7180.9106*	7175.2275
***t* = 24 hr, (+)GSH**	***+X Disulfide 2'-Conjugates***	***Calculated $[M-H]^-$***	**Observed $[M-H]^-$**	***+Y Reduced Thiol 2'-Conjugates***	***Calculated $[M-H]^-$***	**Observed $[M-H]^-$**
	+0	*5646.7955*	5647.4894	*+2*	*5882.8132*	5884.3383
	+1	*5871.8237*	5868.2726	*+4*	*6118.8309*	6121.8007
	+2	*6096.8520*	6096.5332	*+6*	*6354.8486*	6355.1146
	+3	*6321.8802*	6320.0067	*+8*	*6590.8663*	6591.3137
	+4	*6546.9084*	6543.9176	*+9*	*6708.8752*	6708.7176
				+11	*6944.8929*	6949.2576
				+13	*7180.9106*	7175.0408
***t* = 48 hr, (+)GSH**	***+X Disulfide 2'-Conjugates***	***Calculated $[M-H]^-$***	**Observed $[M-H]^-$**	***+Y Reduced Thiol 2'-Conjugates***	***Calculated $[M-H]^-$***	**Observed $[M-H]^-$**
	+0	*5646.7955*	5647.0322	*+2*	*5882.8132*	5879.5640
	+1	*5871.8237*	5869.0784	*+4*	*6118.8309*	6122.5448
	+2	*6096.8520*	6096.8198	*+6*	*6354.8486*	6352.4775
	+3	*6321.8802*	6321.0587			
***t* = 72 hr, (+)GSH**	***+X Disulfide 2'-Conjugates***	***Calculated $[M-H]^-$***	**Observed $[M-H]^-$**	***+Y Reduced Thiol 2'-Conjugates***	***Calculated $[M-H]^-$***	**Observed $[M-H]^-$**

	+0	*5646.7955*	5647.2148	*+2*	*5882.8132*	5880.9581
	+1	*5871.8237*	5867.1798	*+4*	*6118.8309*	6115.2484
	+2	*6096.8520*	6095.1569	*+6*	*6354.8486*	6353.1666
	+3	*6321.8802*	6319.0624	*+8*	*6590.8663*	6586.6140
	+4	*6546.9084*	6548.9257	*+10*	*6826.8840*	6824.1291
	+5	*6771.9366*	6771.7173			

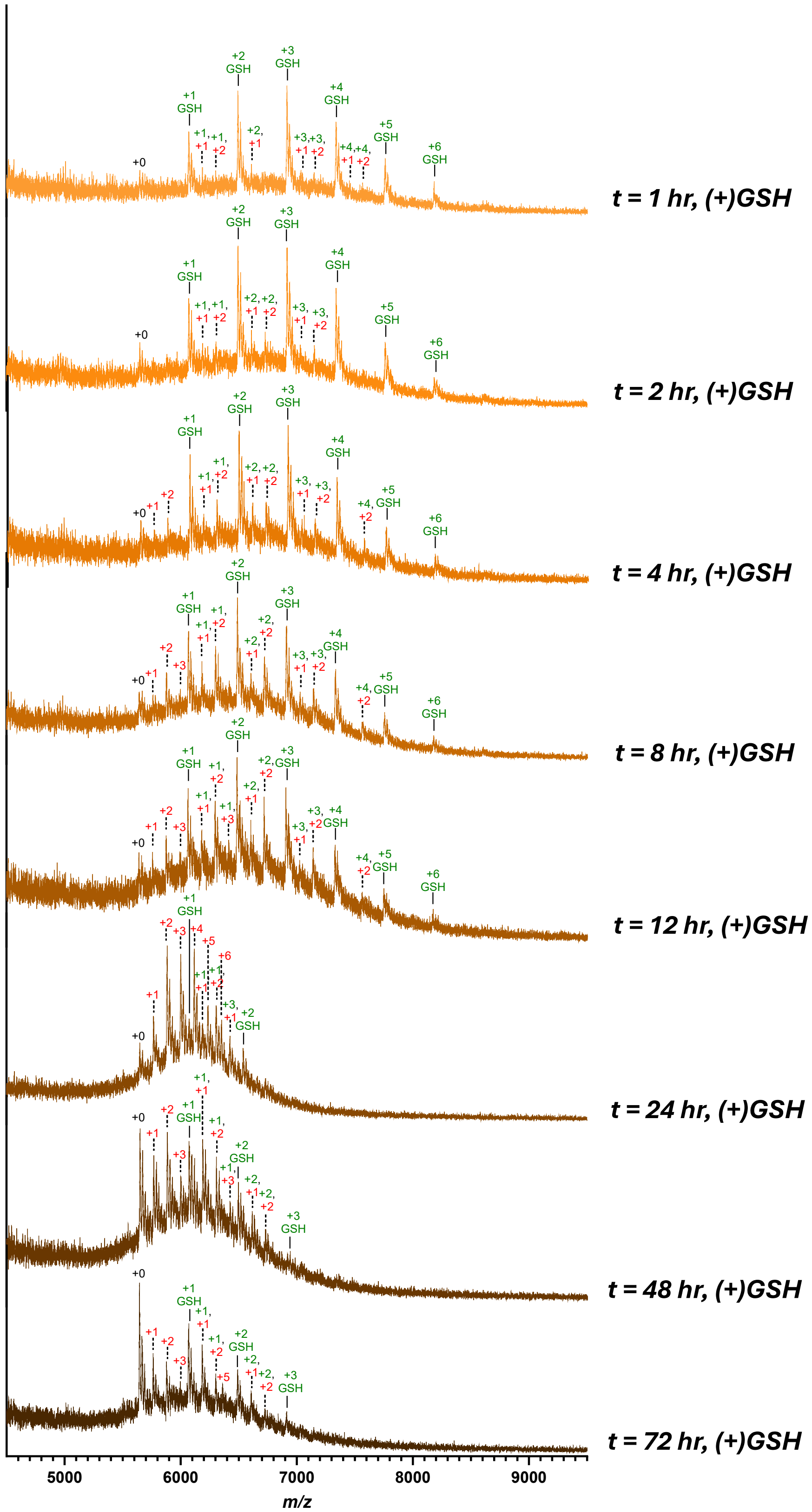

Figure S4D: MALDI-ToF mass spectra of **13**-Acyl-RNA samples incubated in the presence of 10 mM reduced glutathione at various time points, confirming the formation of up to +6 stable RNA-GSH conjugates per RNA strand. The number of intact disulfide conjugates (+X) are denoted in blue, the number of reduced thiol conjugates (+Y) are denoted in red, and the number of RNA-GSH conjugates (+Z) are denoted in green.

Table S11: Calculated and observed *m/z* values based on the above recorded MALDI-ToF spectra for **13**-Acyl-RNA samples in the presence of 10 mM reduced glutathione at various timepoints (Figure S4D). The number of intact disulfide conjugates (+X) are denoted in blue, the number of reduced thiol conjugates (+Y) are denoted in red, and the number of RNA-GSH conjugates (+Z) are denoted in green.

13-Acyl-RNA						
t = 1 hr, (+)GSH	***+X Disulfide 2'-Conjugates***	***Calculated [M-H]-***	***Observed [M-H]-***	***+Y Reduced Thiol 2'-Conjugates***	***Calculated [M-H]-***	***Observed [M-H]-***
	+0	*5646.7955*	5647.2291			
	+Z RNA-GSH 2'-conjugates	***Calculated [M-H]-***	***Observed [M-H]-***	***+Z RNA-GSH / + Y Reduced Thiol 2'-Conjugates***	***Calculated [M-H]-***	***Observed [M-H]-***
	+1	*6069.8726*	6069.6999	*+1, +1*	*6187.8814*	6187.1676
	+2	*6492.9496*	6491.0216	*+1, +2*	*6305.8903*	6301.6293
	+3	*6916.0266*	6915.1883	*+2, +1*	*6610.9584*	6609.7862
	+4	*7339.1036*	7337.8700	*+3, +1*	*7034.0354*	7034.8301
	+5	*7762.1806*	7760.2988	*+3, +2*	*7152.0443*	7149.7997
	+6	*8185.2576*	8182.1742	*+4, +1*	*7457.1124*	7459.9782
				+4, +2	*7575.1213*	7574.5996
t = 2 hr, (+)GSH	***+X Disulfide 2'-Conjugates***	***Calculated [M-H]-***	***Observed [M-H]-***	***+Y Reduced Thiol 2'-Conjugates***	***Calculated [M-H]-***	***Observed [M-H]-***
	+0	*5646.7955*	5647.8772	*+1*	*5764.8044*	5764.9265
				+2	*5882.8132*	5882.5034
	+Z RNA-GSH 2'-conjugates	***Calculated [M-H]-***	***Observed [M-H]-***	***+Z RNA-GSH / + Y Reduced Thiol 2'-Conjugates***	***Calculated [M-H]-***	***Observed [M-H]-***
	+1	*6069.8726*	6070.72904	*+1, +1*	*6187.8814*	6188.1290
	+2	*6492.9496*	6494.1348	*+1, +2*	*6305.8903*	6304.6438
	+3	*6916.0266*	6918.7903	*+2, +1*	*6610.9584*	6609.7549
	+4	*7339.1036*	7340.0659	*+2, +2*	*6728.9673*	6728.9407
	+5	*7762.1806*	7762.0736	*+3, +1*	*7034.0354*	7030.4934
	+6	*8185.2576*	8183.3982	*+3, +2*	*7152.0443*	7152.6788
t = 4 hr, (+)GSH	***+X Disulfide 2'-Conjugates***	***Calculated [M-H]-***	***Observed [M-H]-***	***+Y Reduced Thiol 2'-Conjugates***	***Calculated [M-H]-***	***Observed [M-H]-***
	+0	*5646.7955*	5646.8226	*+1*	*5764.8044*	5763.0489
				+2	*5882.8132*	5881.5932
	+Z RNA-GSH 2'-conjugates	***Calculated [M-H]-***	***Observed [M-H]-***	***+Z RNA-GSH / + Y Reduced Thiol 2'-Conjugates***	***Calculated [M-H]-***	***Observed [M-H]-***
	+1	*6069.8726*	6071.0618	*+1, +1*	*6187.8814*	6187.1965
	+2	*6492.9496*	6493.8664	*+1, +2*	*6305.8903*	6302.7338
	+3	*6916.0266*	6914.9276	*+2, +1*	*6610.9584*	6610.1568
	+4	*7339.1036*	7338.2466	*+2, +2*	*6728.9673*	6727.1306
	+5	*7762.1806*	7762.1346	*+3, +1*	*7034.0354*	7035.3377
	+6	*8185.2576*	8183.0081	*+3, +2*	*7152.0443*	7151.8996
				+4, +2	*7575.1213*	7576.3404
t = 8 hr, (+)GSH	***+X Disulfide 2'-Conjugates***	***Calculated [M-H]-***	***Observed [M-H]-***	***+Y Reduced Thiol 2'-Conjugates***	***Calculated [M-H]-***	***Observed [M-H]-***
	+0	*5646.7955*	5645.7263	*+1*	*5764.8044*	5762.5165
				+2	*5882.8132*	5880.0033
				+3	*6000.8221*	5998.0931
	+Z RNA-GSH 2'-conjugates	***Calculated [M-H]-***	***Observed [M-H]-***	***+Z RNA-GSH / + Y Reduced Thiol 2'-Conjugates***	***Calculated [M-H]-***	***Observed [M-H]-***
	+1	*6069.8726*	6067.8332	*+1, +1*	*6187.8814*	6187.0801
	+2	*6492.9496*	6487.9742	*+1, +2*	*6305.8903*	6300.6676
	+3	*6916.0266*	6912.3186	*+2, +1*	*6610.9584*	6606.7314
	+4	*7339.1036*	7334.2449	*+2, +2*	*6728.9673*	6724.8639
	+5	*7762.1806*	7758.3552	*+3, +1*	*7034.0354*	7031.8539
	+6	*8185.2576*	8182.7735	*+3, +2*	*7152.0443*	7147.7925
				+4, +2	*7575.1213*	7574.2775

t = 12 hr, (+)GSH	***+X Disulfide 2'-Conjugates***	***Calculated $[M-H]^-$***	***Observed $[M-H]^-$***	***+Y Reduced Thiol 2'-Conjugates***	***Calculated $[M-H]^-$***	***Observed $[M-H]^-$***
	+0	*5646.7955*	5642.2747	*+1*<br>*+2*<br>*+3*	*5764.8044*<br>*5882.8132*<br>*6000.8221*	5760.5715<br>5877.2098<br>6000.0192
	+Z RNA-GSH 2'-conjugates	***Calculated $[M-H]^-$***	***Observed $[M-H]^-$***	***+Z RNA-GSH / + Y Reduced Thiol 2'-Conjugates***	***Calculated $[M-H]^-$***	***Observed $[M-H]^-$***
	+1<br>*+2*<br>*+3*<br>*+4*<br>*+5*<br>*+6*	*6069.8726*<br>*6492.9496*<br>*6916.0266*<br>*7339.1036*<br>*7762.1806*<br>*8185.2576*	6063.6045<br>6487.3712<br>6913.6411<br>7336.5780<br>7762.1173<br>8187.5907	*+1, +1*<br>*+1, +2*<br>*+1, +3*<br>*+2, +1*<br>*+2, +2*<br>*+3, +1*<br>*+3, +2*<br>*+4, +2*	*6187.8814*<br>*6305.8903*<br>*6423.8991*<br>*6610.9584*<br>*6728.9673*<br>*7034.0354*<br>*7152.0443*<br>*7575.1213*	6186.2113<br>6300.6343<br>6428.5225<br>6606.4754<br>6727.0723<br>7033.6649<br>7148.6200<br>7572.1851
t = 24 hr, (+)GSH	***+X Disulfide 2'-Conjugates***	***Calculated $[M-H]^-$***	***Observed $[M-H]^-$***	***+Y Reduced Thiol 2'-Conjugates***	***Calculated $[M-H]^-$***	***Observed $[M-H]^-$***
	+0	*5646.7955*	5648.7485	*+1*<br>*+2*<br>*+3*<br>*+4*<br>*+5*<br>*+6*	*5764.8044*<br>*5882.8132*<br>*6000.8221*<br>*6118.8309*<br>*6236.8398*<br>*6354.8486*	5766.8077<br>5884.4107<br>6001.3710<br>6119.1583<br>6237.1408<br>6353.2492
	+Z RNA-GSH 2'-conjugates	***Calculated $[M-H]^-$***	***Observed $[M-H]^-$***	***+Z RNA-GSH / + Y Reduced Thiol 2'-Conjugates***	***Calculated $[M-H]^-$***	***Observed $[M-H]^-$***
	+1<br>*+2*	*6069.8726*<br>*6492.9496*	6068.8751<br>6493.3344	*+1, +1*<br>*+1, +2*<br>*+1, +3*	*6187.8814*<br>*6305.8903*<br>*6423.8991*	6187.4860<br>6306.2556<br>6422.2880
t = 48 hr, (+)GSH	***+X Disulfide 2'-Conjugates***	***Calculated $[M-H]^-$***	***Observed $[M-H]^-$***	***+Y Reduced Thiol 2'-Conjugates***	***Calculated $[M-H]^-$***	***Observed $[M-H]^-$***
	+0	*5646.7955*	5644.6307	*+1*<br>*+2*<br>*+3*	*5764.8044*<br>*5882.8132*<br>*6000.8221*	5763.2454<br>5880.5269<br>5997.2836
	+Z RNA-GSH 2'-conjugates	***Calculated $[M-H]^-$***	***Observed $[M-H]^-$***	***+Z RNA-GSH / + Y Reduced Thiol 2'-Cnjugates***	***Calculated $[M-H]^-$***	***Observed $[M-H]^-$***
	+1<br>*+2*<br>*+3*	*6069.8726*<br>*6492.9496*<br>*6916.0266*	6069.1551<br>6491.5095<br>6914.7695	*+1, +1*<br>*+1, +2*<br>*+1, +3*<br>*+2, +1*<br>*+2, +2*	*6187.8814*<br>*6305.8903*<br>*6423.8991*<br>*6610.9584*<br>*6728.9673*	6185.9729<br>6302.8382<br>6421.8729<br>6608.1302<br>6725.7494
t = 72 hr, (+)GSH	***+X Disulfide 2'-Conjugates***	***Calculated $[M-H]^-$***	***Observed $[M-H]^-$***	***+Y Reduced Thiol 2'-Conjugates***	***Calculated $[M-H]^-$***	***Observed $[M-H]^-$***
	+0	*5646.7955*	5645.8123	*+1*<br>*+2*<br>*+3*<br>*+5*	*5764.8044*<br>*5882.8132*<br>*6000.8221*<br>*6236.8398*	5764.6564<br>5880.5997<br>5997.4078<br>6233.4080
	+Z RNA-GSH 2'-conjugates	***Calculated $[M-H]^-$***	***Observed $[M-H]^-$***	***+Z RNA-GSH / + Y Reduced Thiol 2'-Conjugates***	***Calculated $[M-H]^-$***	***Observed $[M-H]^-$***
	+1<br>*+2*<br>*+3*	*6069.8726*<br>*6492.9496*<br>*6916.0266*	6070.4482<br>6494.3441<br>6913.0294	*+1, +1*<br>*+1, +2*<br>*+2, +1*<br>*+2, +2*	*6187.8814*<br>*6305.8903*<br>*6610.9584*<br>*6728.9673*	6187.4565<br>6304.5446<br>6610.4781<br>6726.4438

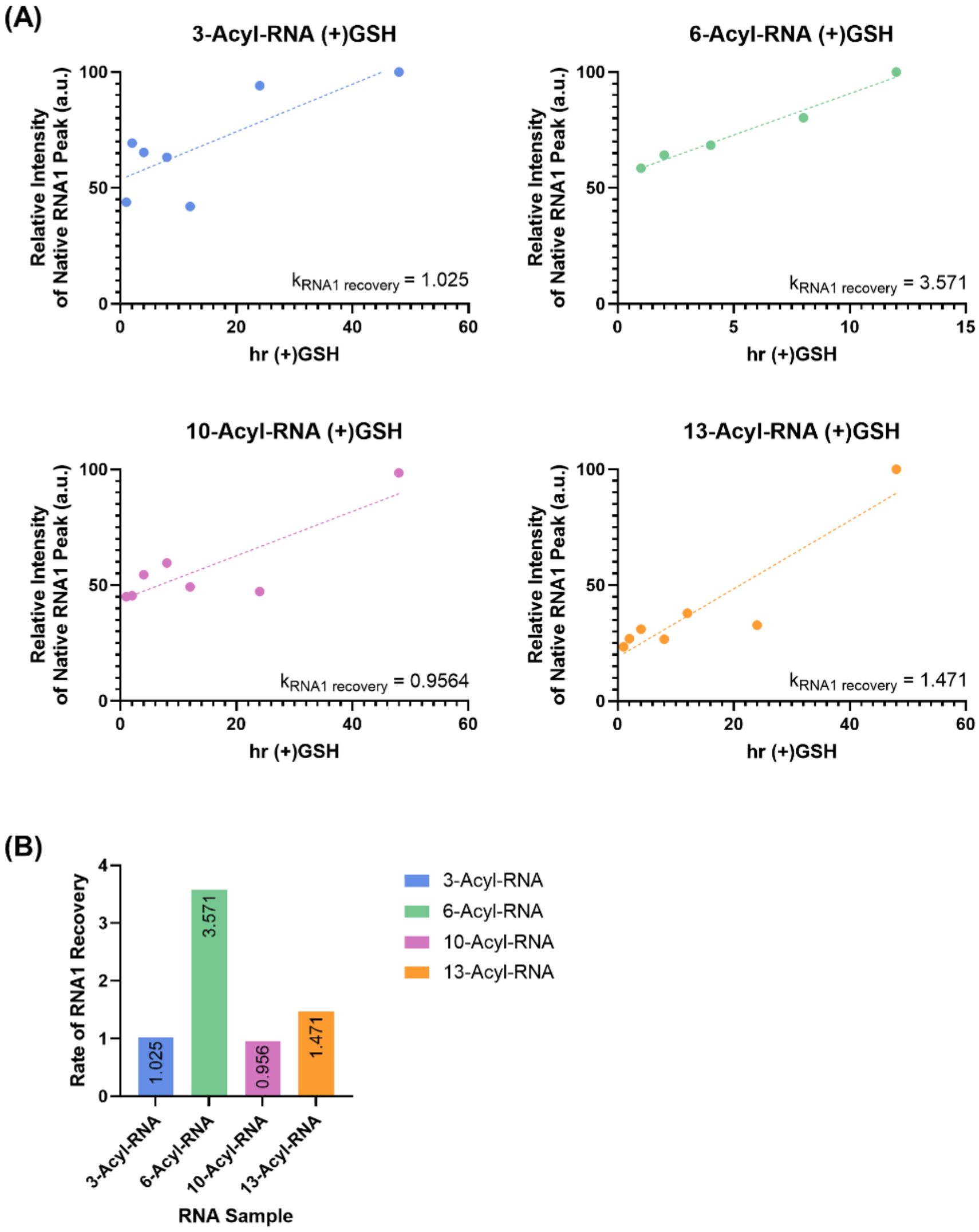

Figure S4E: (A) Increase in the relative intensity of the native **RNA1** *m/z* signal from the recorded MALDI-ToF mass spectra reported in Figures S4A-D, with the relative rates of **RNA1** recovery (k) extrapolated from the corresponding lines of best fit. (B) Relative rates of **RNA1** recovery (k) from **3**-Acyl-RNA, **6**-Acyl-RNA, **10**-Acyl-RNA and **13**-Acyl-RNA samples incubated in the presence of 10 mM reduced glutathione at 37 °C.

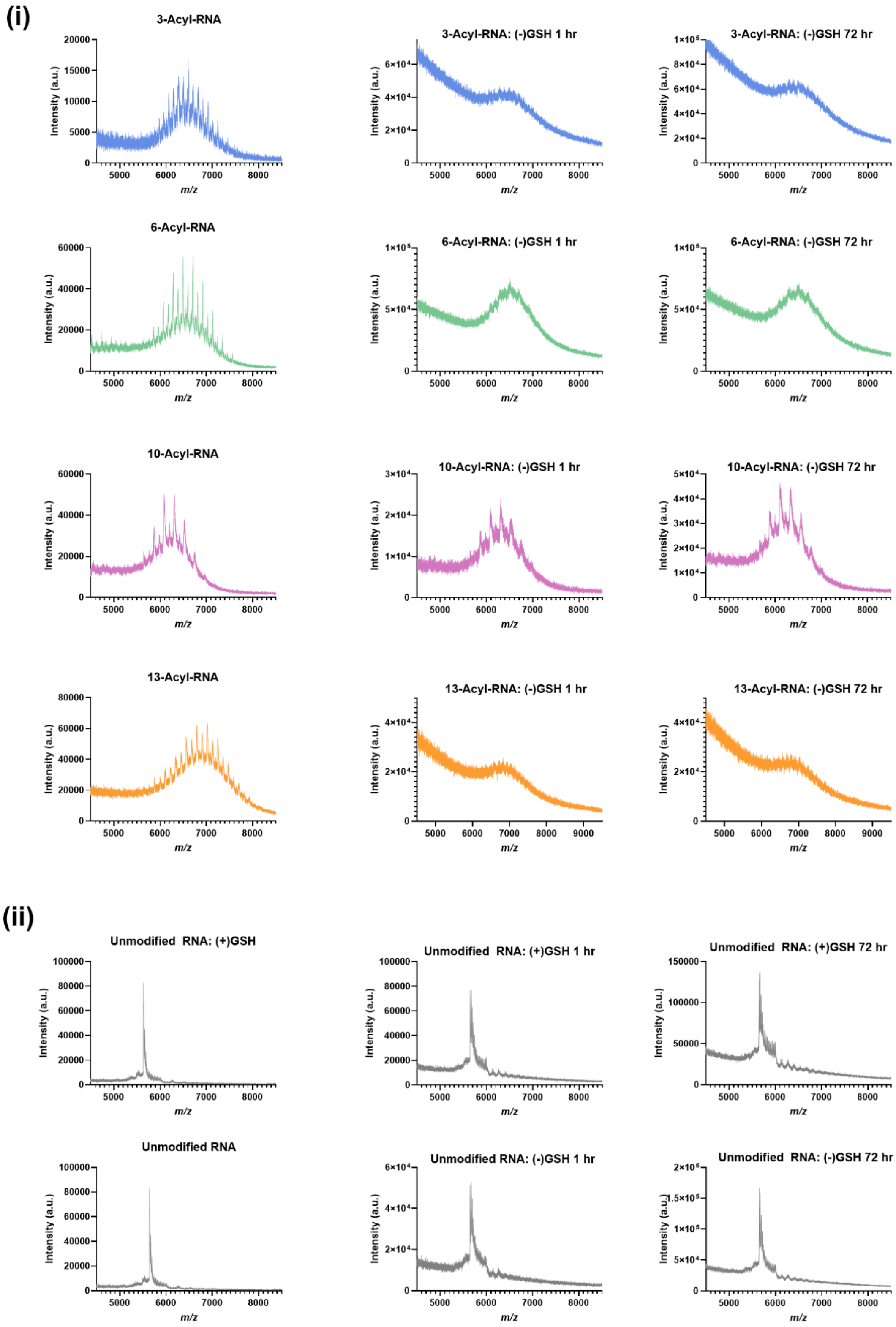

Figure S5: MALDI-ToF mass spectra indicating (i) the aqueous stability of Acyl-RNA strands in (-)GSH conditions (10 mM sodium phosphate buffer, pH = 7.4, 37 °C) at 1 hr and 72 hr incubation and (ii) the aqueous stability of unmodified RNA in (+)GSH conditions (10 mM reduced glutathione, 10 mM sodium phosphate buffer, pH = 7.4, 37 °C) and (-)GSH conditions (10 mM sodium phosphate buffer, pH = 7.4, 37 °C) at 1 hr and 72 hr incubation.

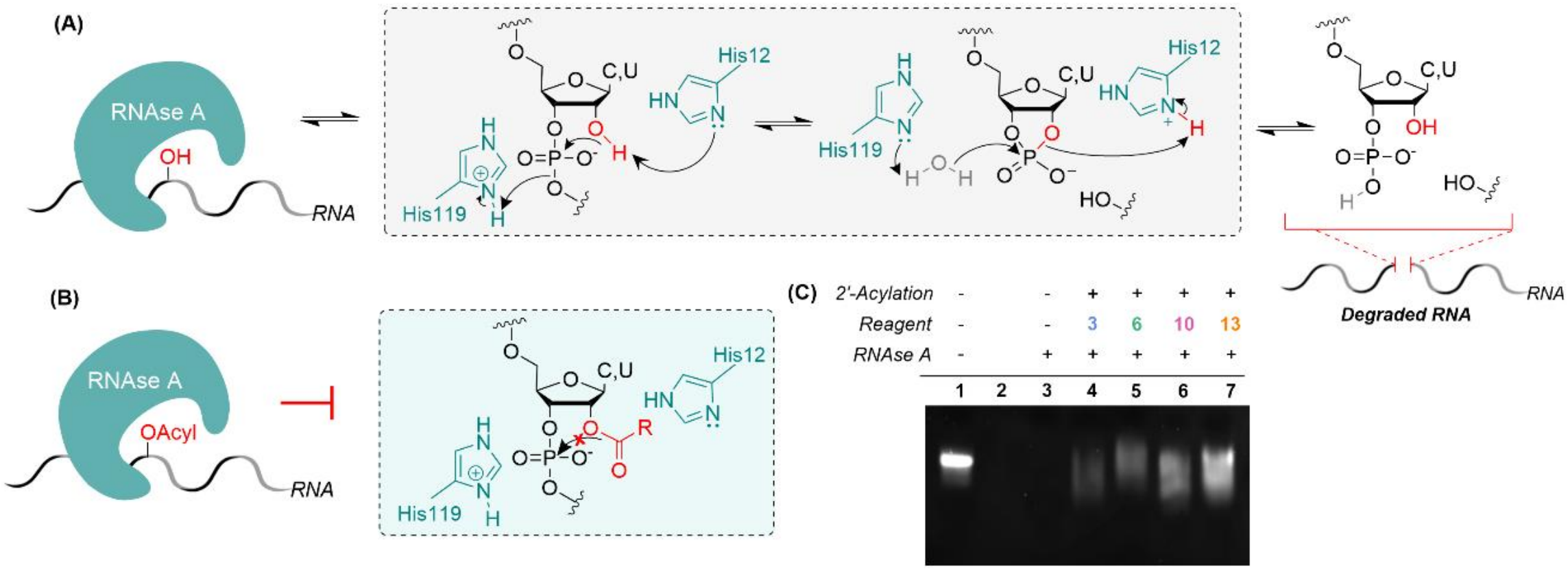

Figure S6: (A) RNase A-mediated digestion of an unmodified, single-stranded RNA at the 3'-end of pyrimidine residues.[56] (B) Reduced RNase A recognition of a 2'-acylated single-stranded RNA, resulting in a lower extent of endoribonuclease digestion. (C) Evaluation of RNase A-mediated digestion of unmodified RNA (Lane 3) and **3**-, **6**-, **10**- and **13**-Acyl-RNA samples (Lanes 4-7) *via* urea-PAGE.

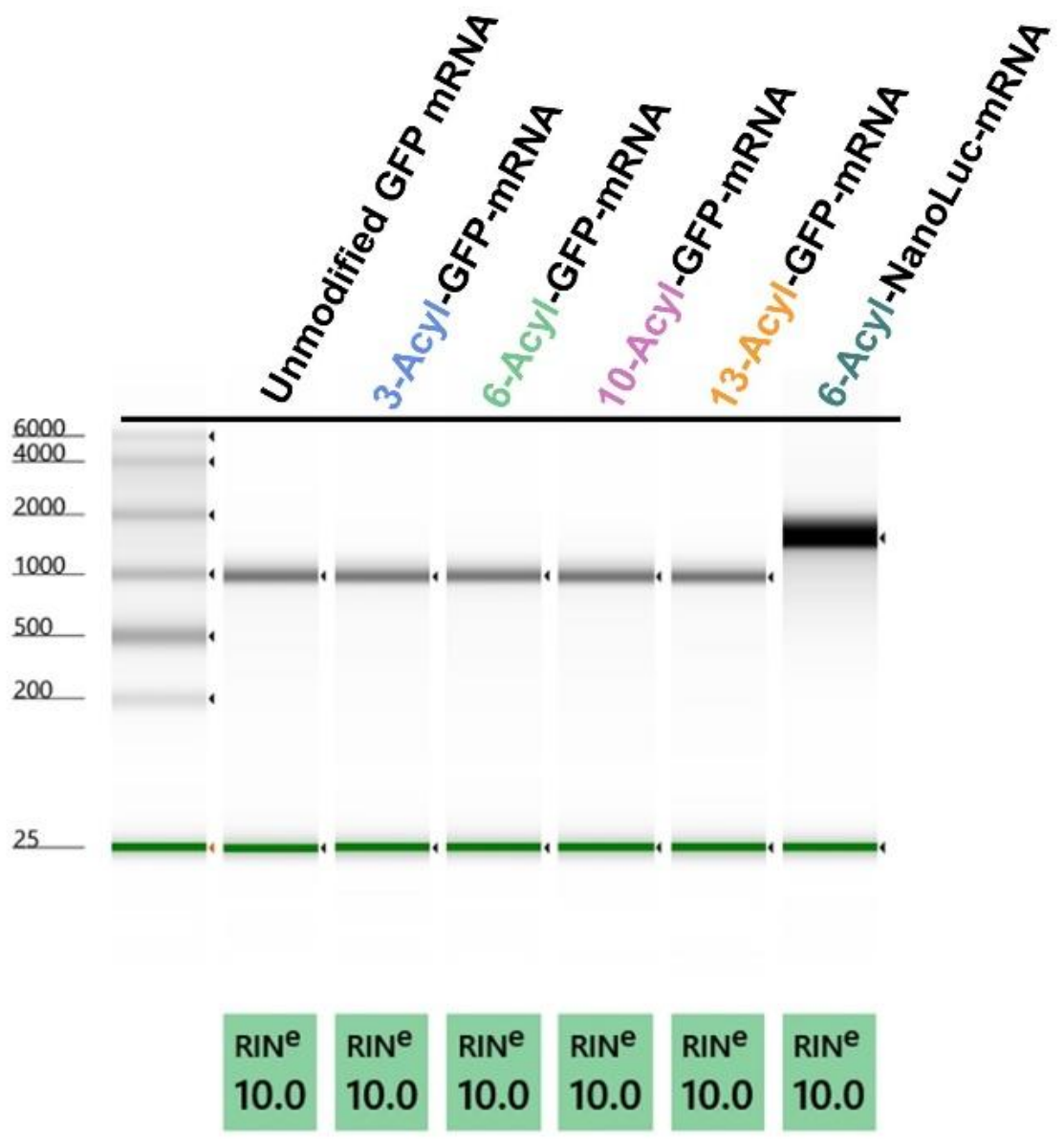

Figure S7. Assessment of mRNA integrity after 2'-acylation *via* Tapestation capillary electrophoresis. Digitalized gel images of GFP and NanoLuc IVT mRNA modified with **3**, **6**, **10**, and **13** are shown. All 2'-acylated mRNA samples exhibit a sharp, full-length peak at the expected size with minimal baseline noise and no detectable degradation products. Peak profiles and RNA Integrity Number equivalent (RINe) values remain consistent with unmodified mRNA controls, demonstrating that the acylation procedure preserves transcript integrity across all constructs.

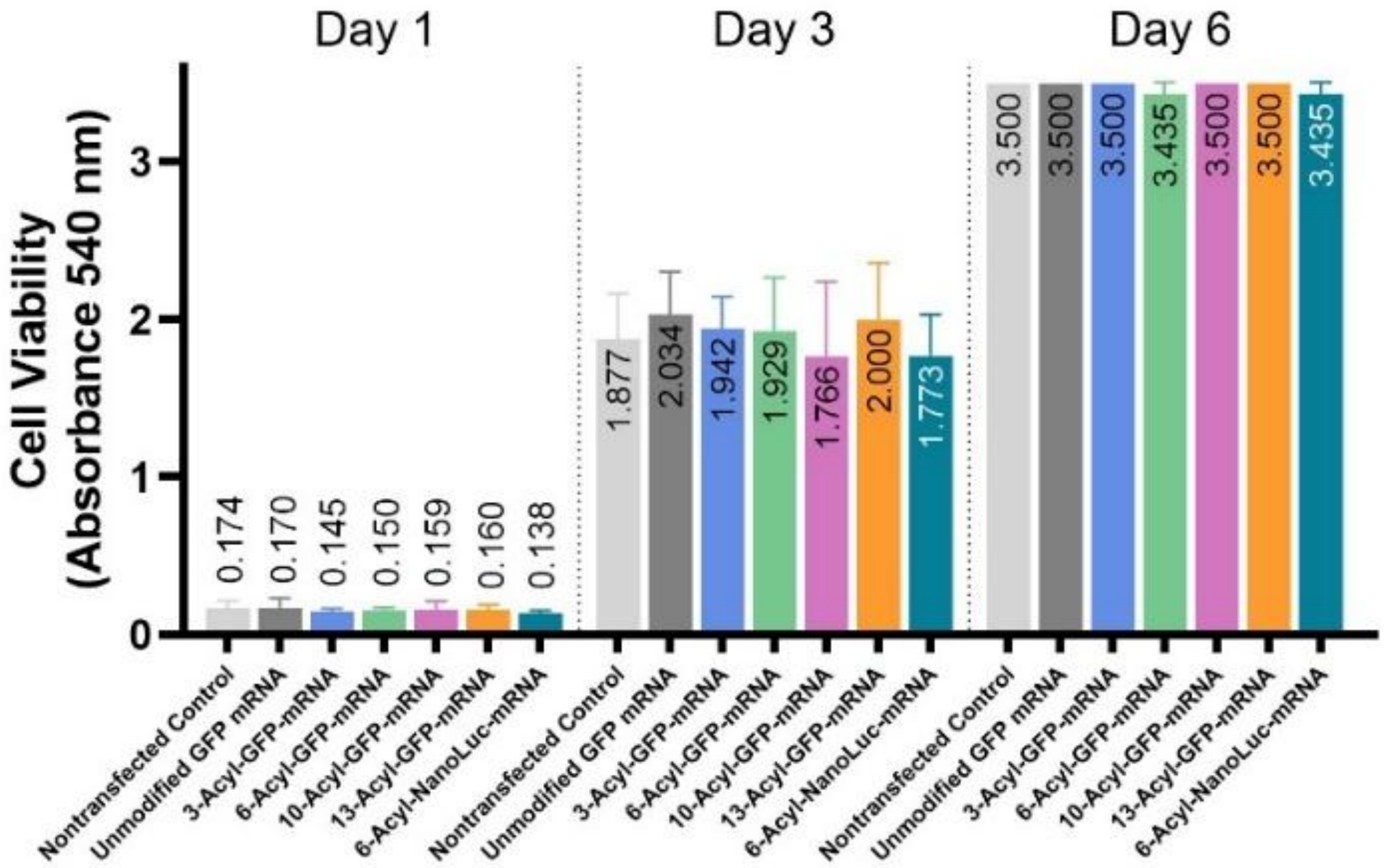

Figure S8. Cytotoxicity of Acyl-GFP-mRNA and Acyl-NanoLuc-mRNA constructs in HEK293 cells assessed by MTT assay. HEK293 cells were transfected with GFP IVT mRNA and Nanoluciferase IVT mRNA 2'-conjugated with reagents **3**, **6**, **10**, and **13**, and cell viability was measured on day 1, day 3, and day 6 post-transfection using the MTT assay. Formazan production was quantified by absorbance at 540 nm and normalised to untreated control cells (mean ± SD; *n* = 3 independent experiments).

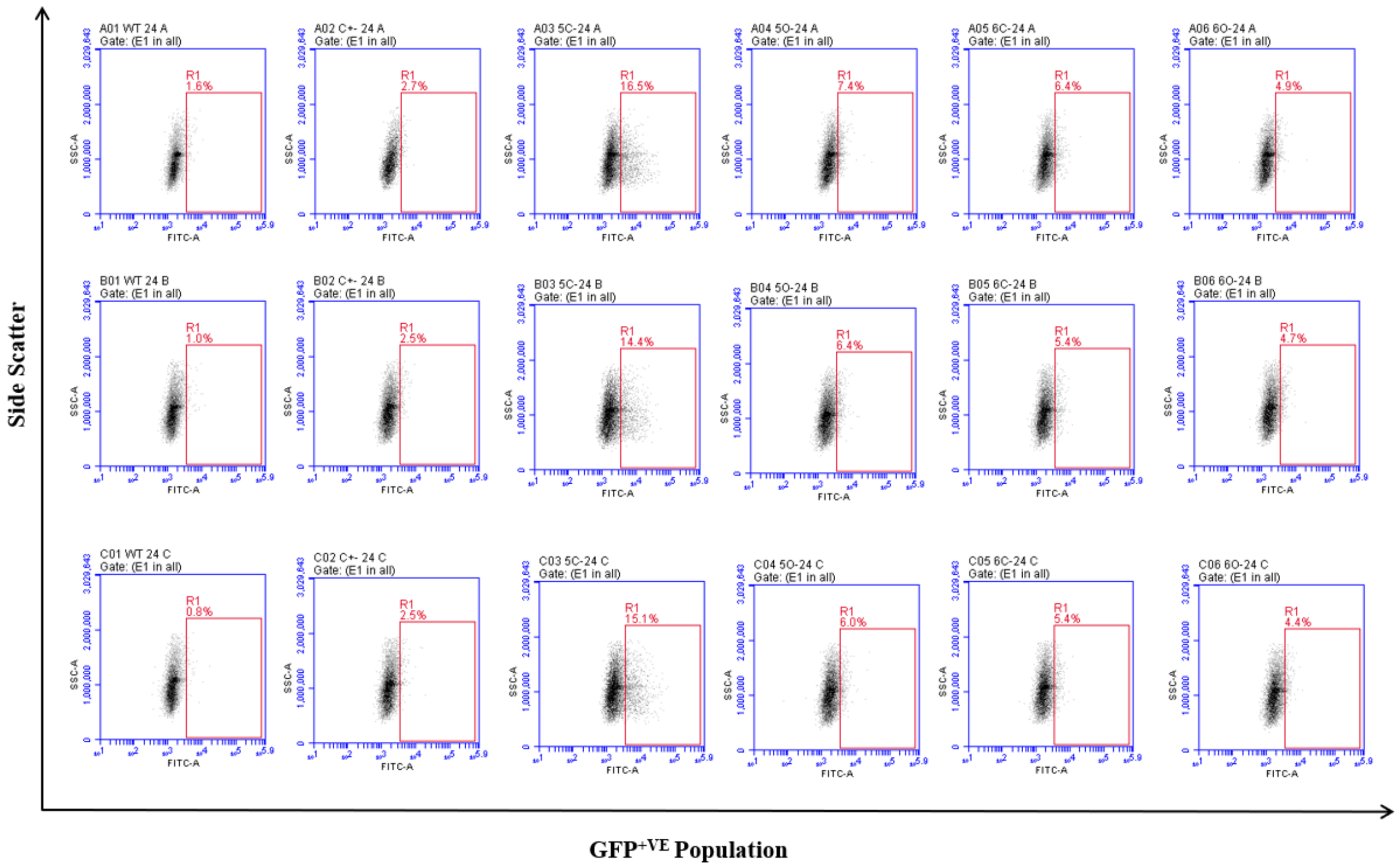

Figure S9: Representative gating strategies indicating the effect of **3**, **6**, **10** & **13** on GFP expression in HEK293 cells.

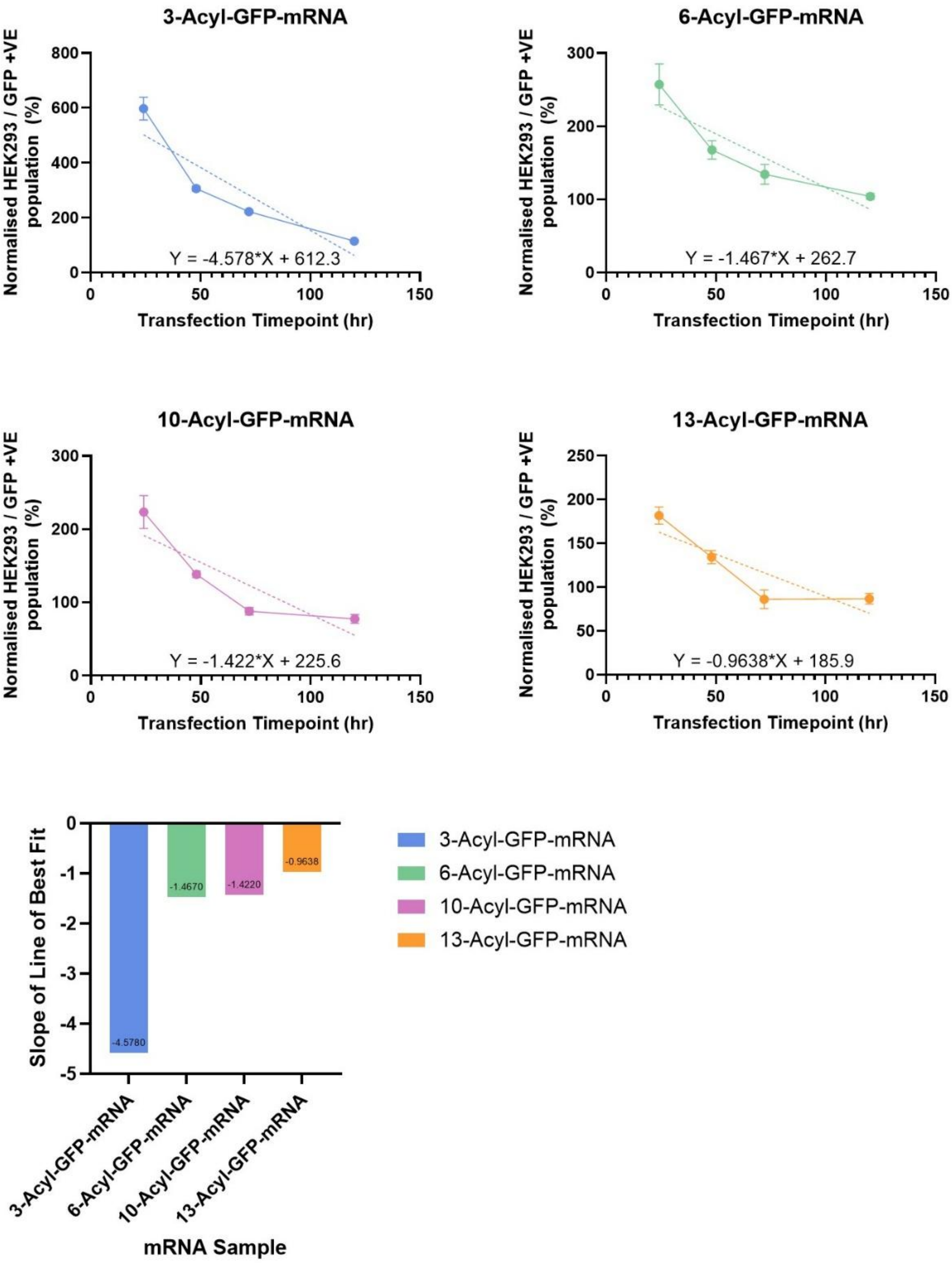

Figure S10: (A) Population percentages of HEK293 cells expressing GFP signal obtained from flow cytometry analysis for Acyl-GFP-mRNA samples conjugated to **3**, **6**, **10** & **13** respectively, normalised to an unmodified GFP mRNA control at each transfection timepoint. (B) Rates of decreasing GFP fluorescent signal for Acyl-GFP-mRNA samples, based on the individual slopes of the lines of best fit.

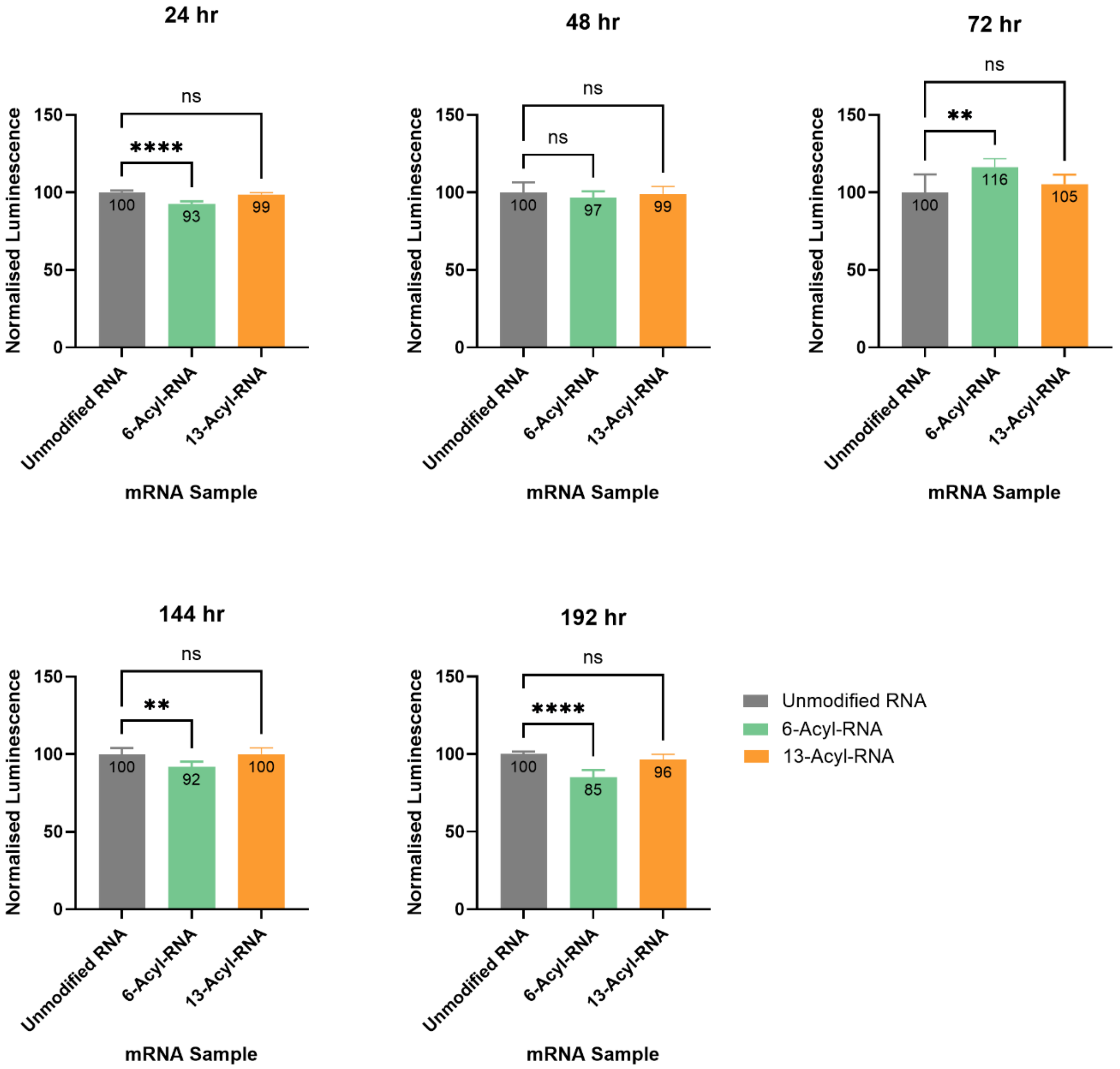

Figure S11: Nanoluciferase activity in HEK293 cells of unmodified nanoluciferase mRNA, **6**-Acyl-NanoLuc-mRNA and **13**-Acyl-NanoLuc-mRNA, quantified using Nano-Glo Luciferase Assay System at 24, 48, 72, 144 & 192 hr post-transfection and normalised to the unmodified nanoluciferase mRNA control (mean ± SD; *n* = 6 independent experiments). Statistical significance was calculated using one-way ANOVA followed by Tukey's post-hoc test (*ns* = non-significant, *$p \leq 0.05$, **$p \leq 0.01$, and ****$p \leq 0.0001$.)

^{1}H & ^{13}C NMR Spectra and LC/MS (ESI+) Mass Spectra

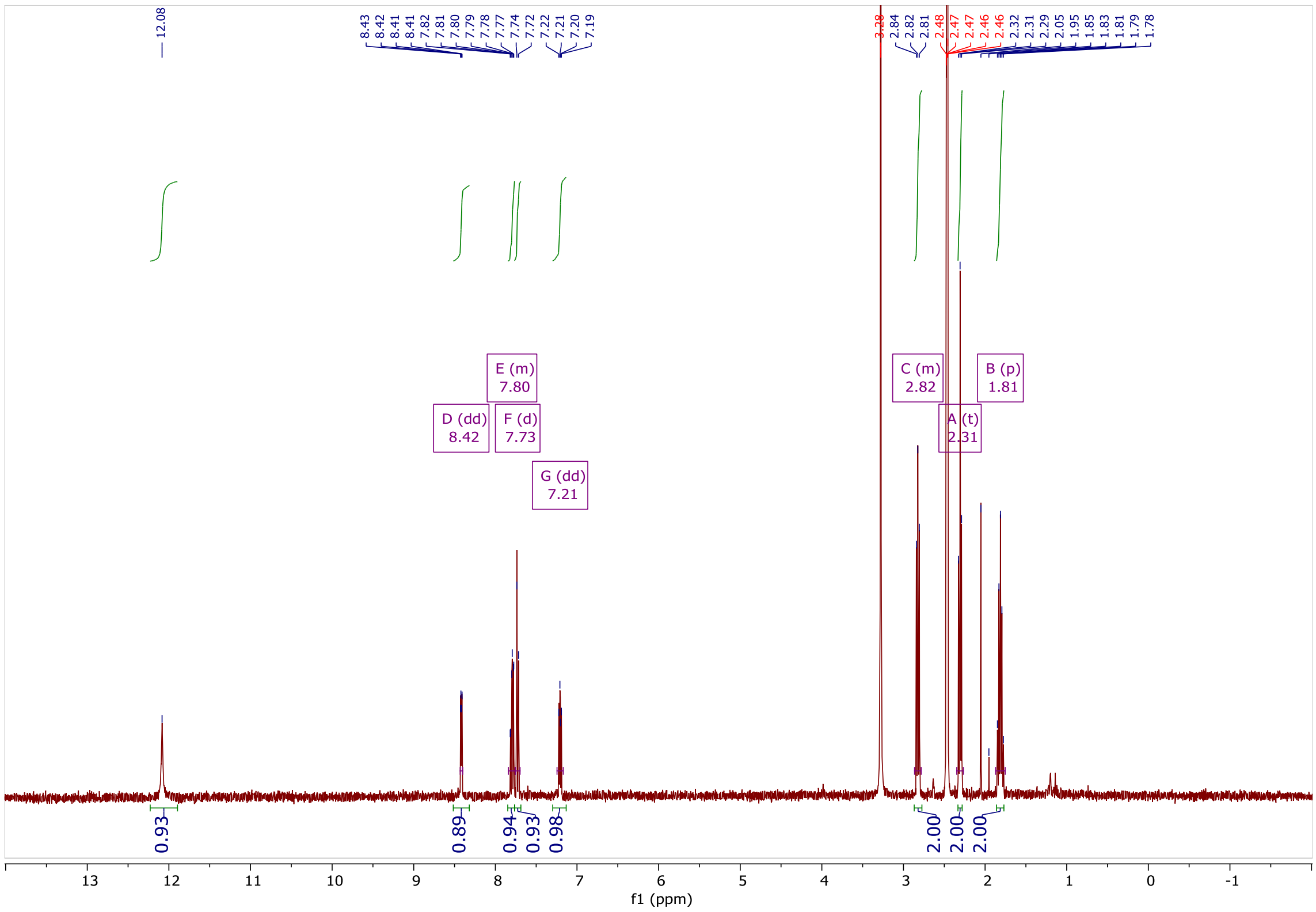

Figure S12A: ^{1}H NMR spectrum of **2**.

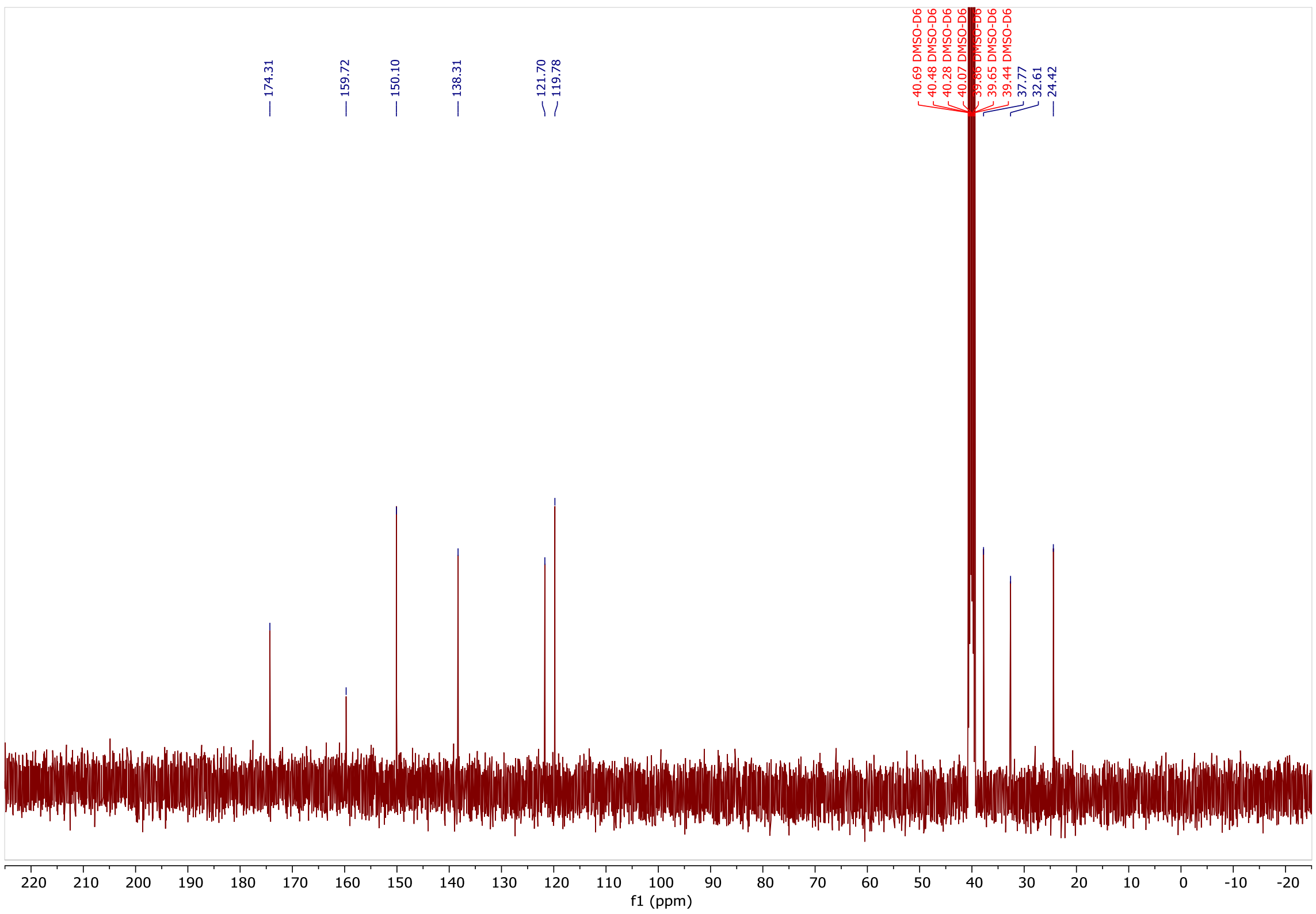

Figure S12B: ^{13}C NMR spectrum of **2**.

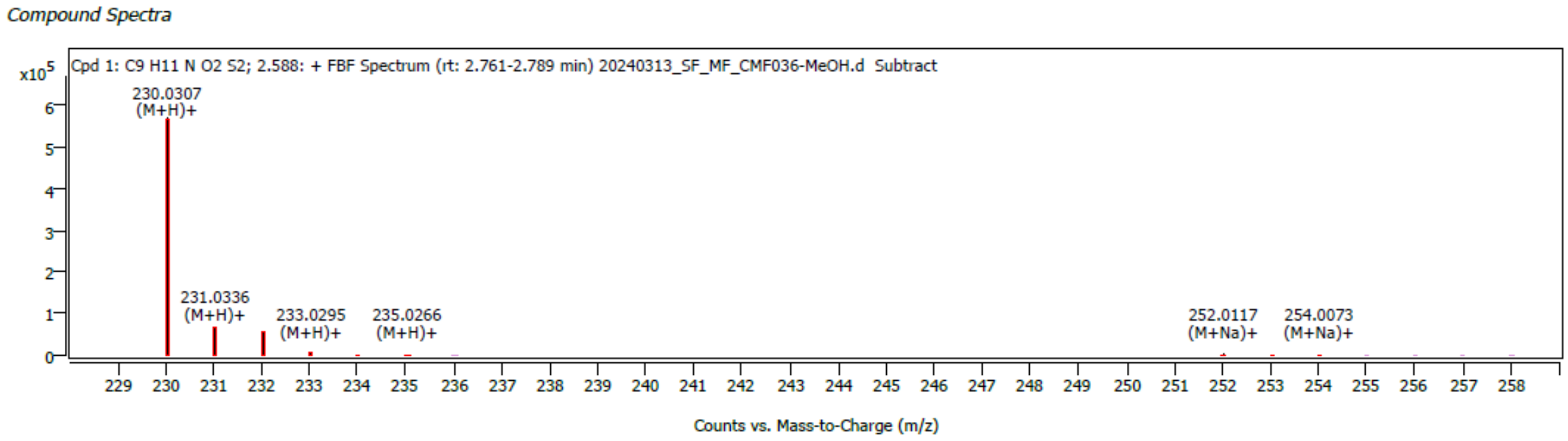

Figure S12C: LC/MS (ESI+) mass spectrum of **2**.

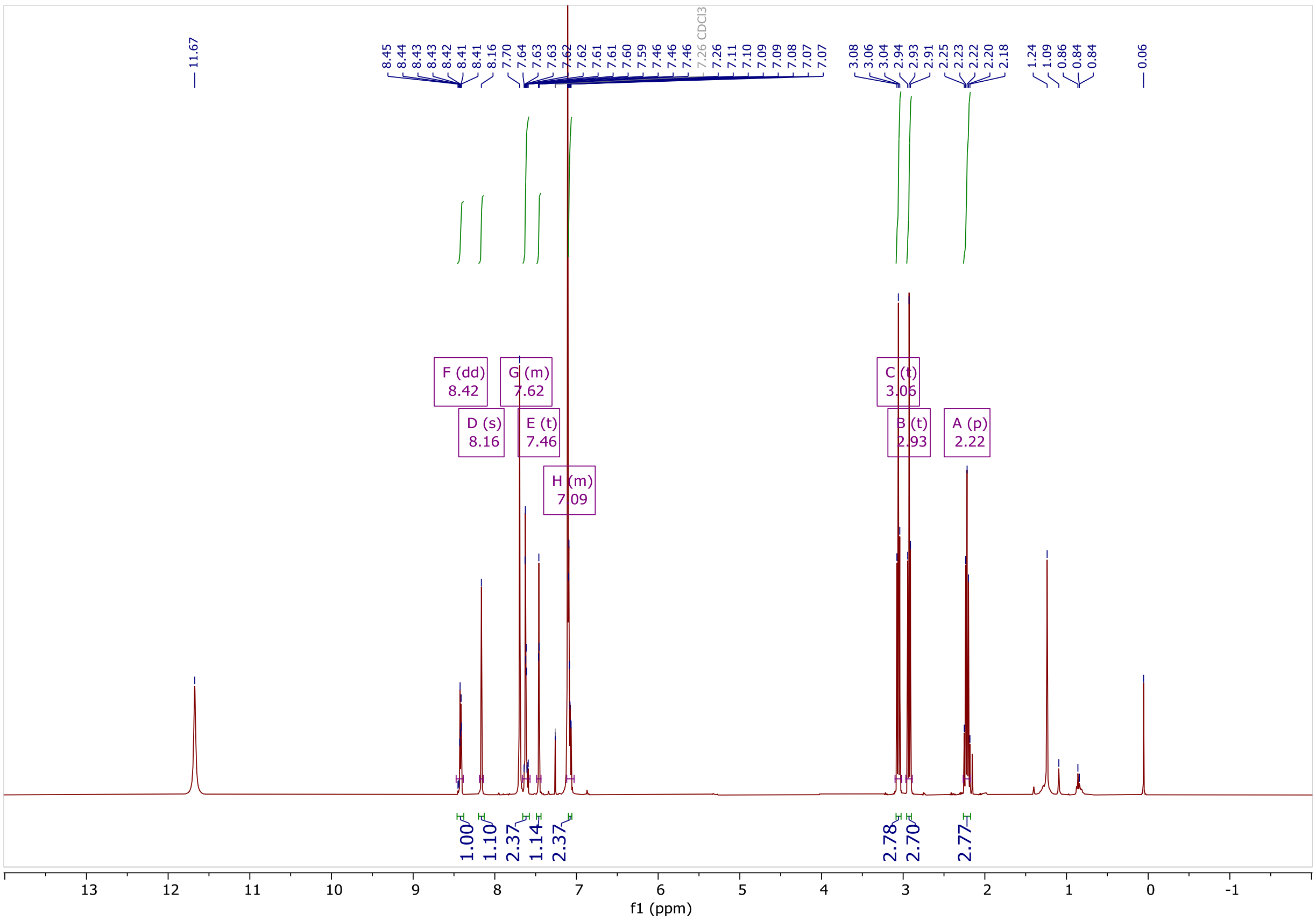

Figure S13A: ^{1}H NMR spectrum of **3**.

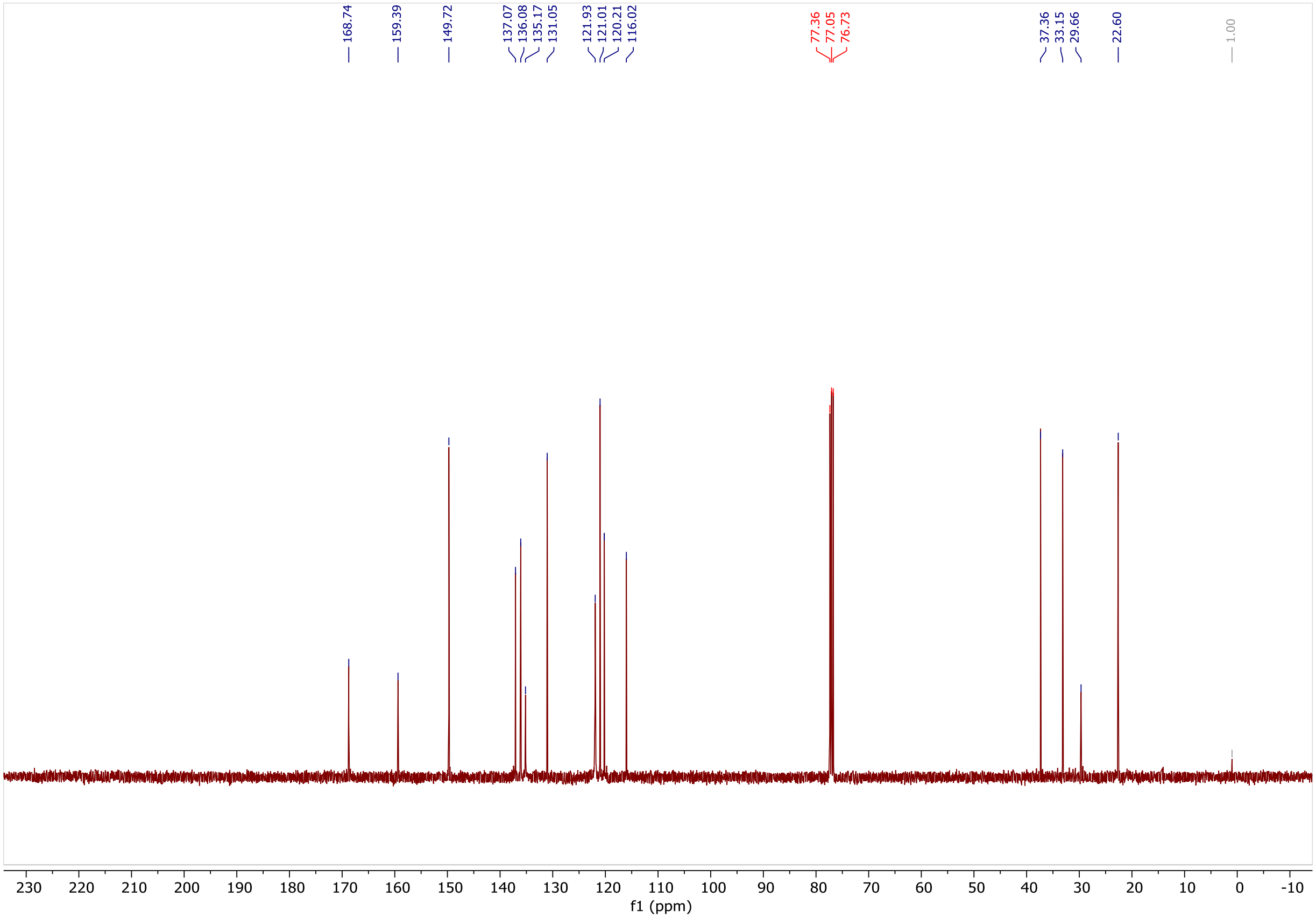

Figure S13B: ^{13}C NMR spectrum of **3**.

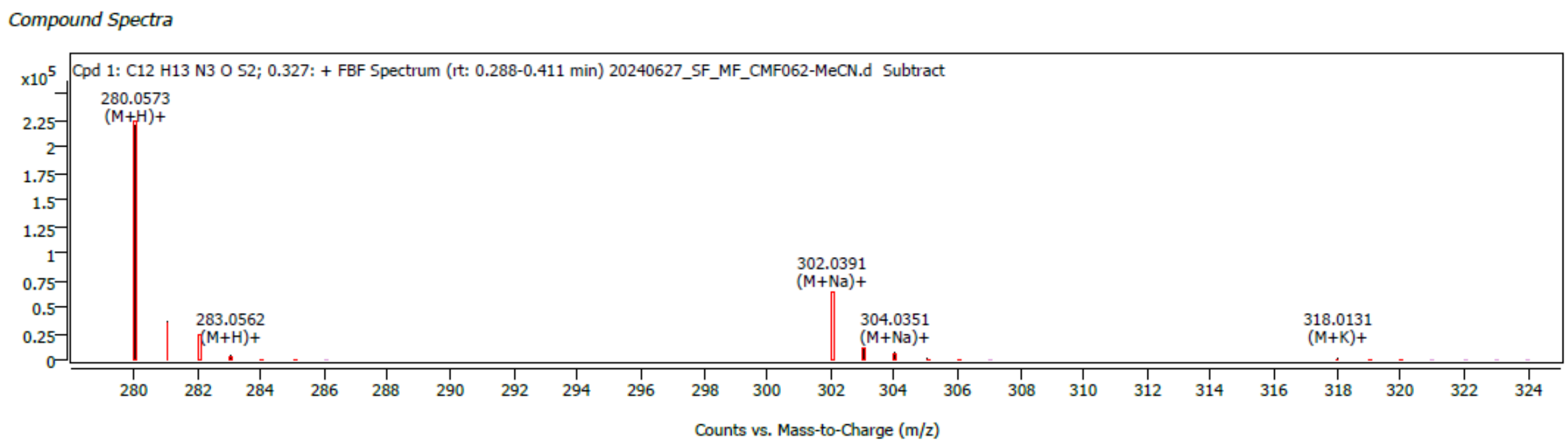

Figure S13C: LC/MS (ESI+) mass spectrum of **3**.

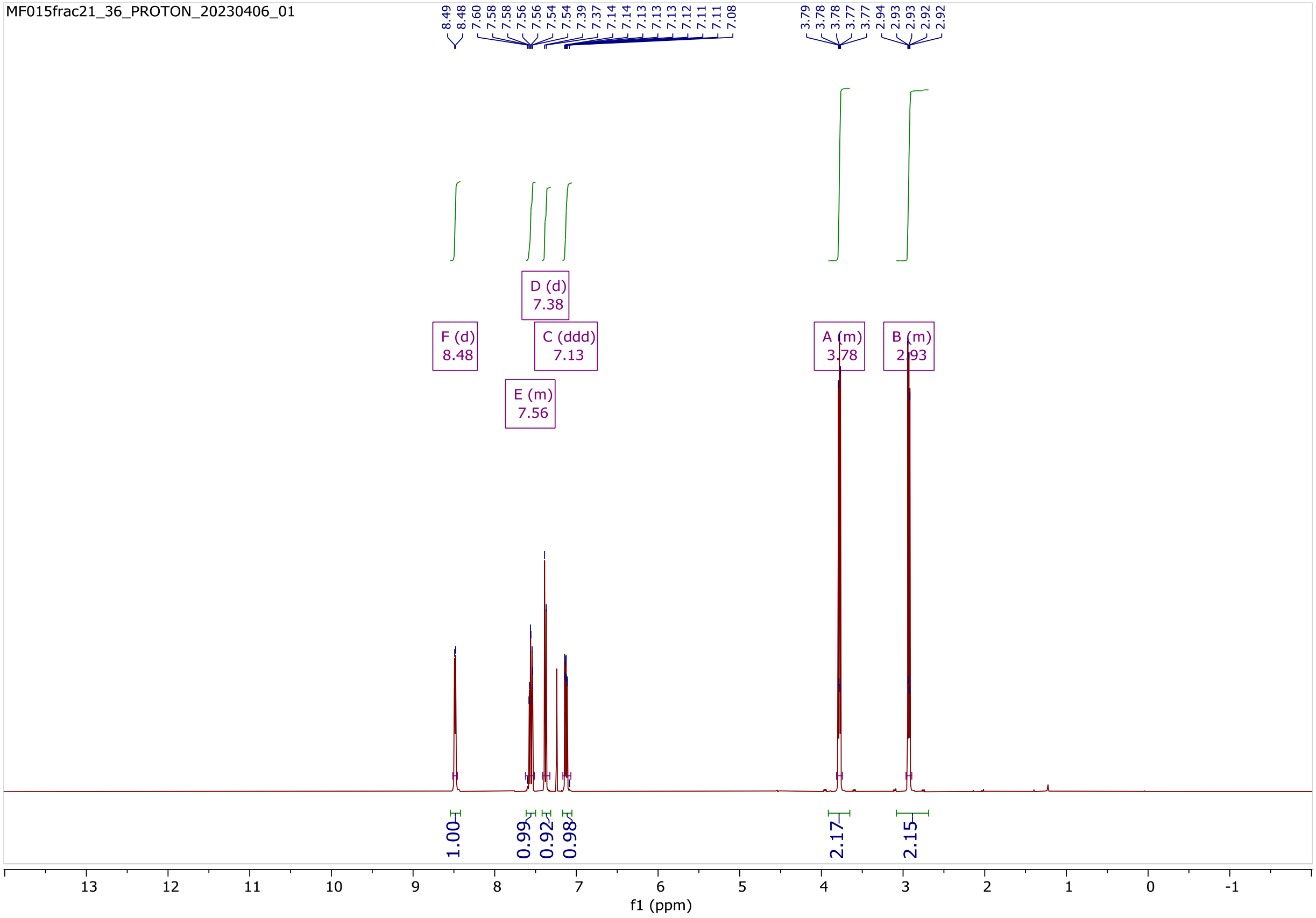

Figure S14A: ^{1}H NMR spectrum of **5**.

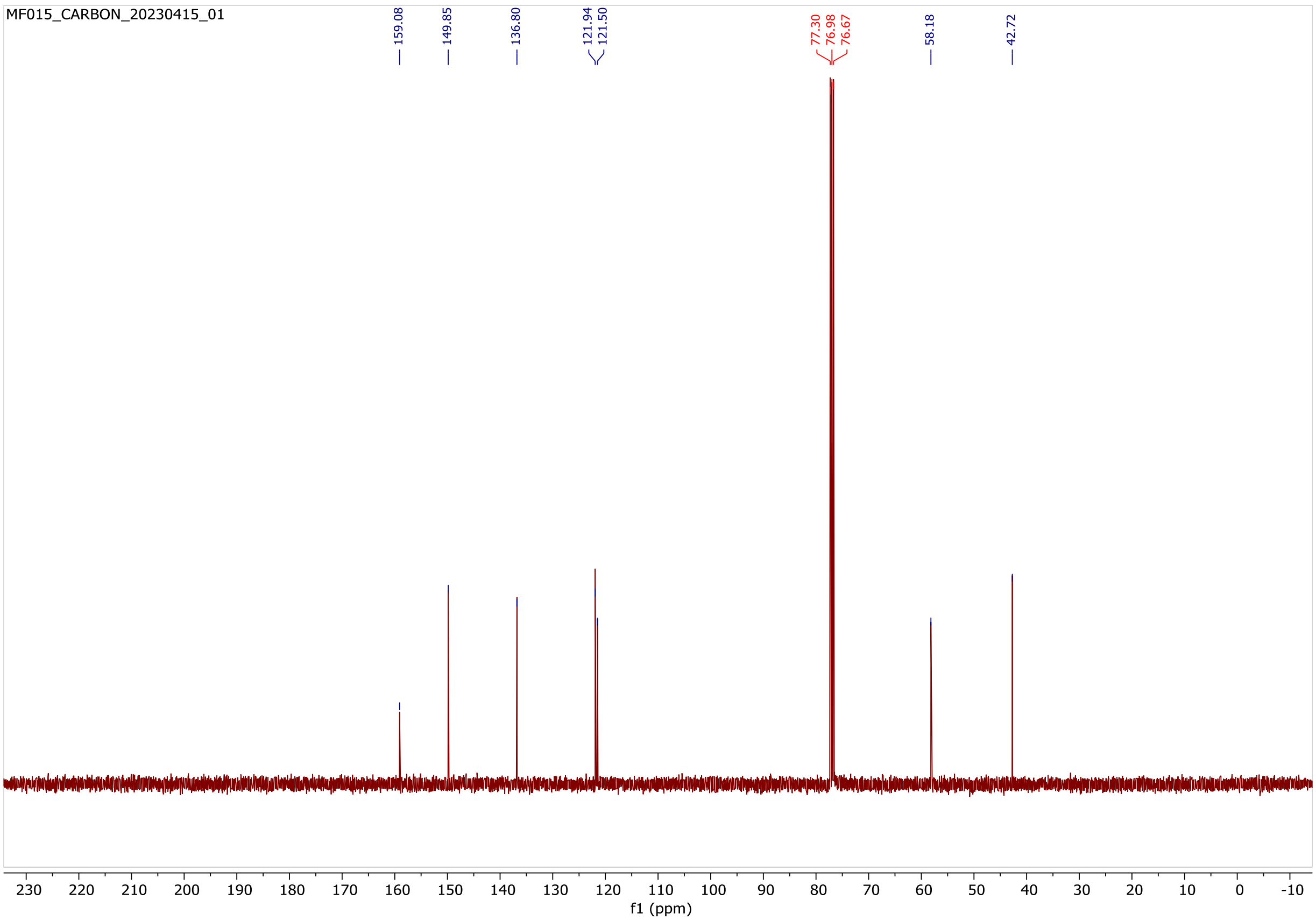

Figure S14B: ^{13}C NMR spectrum of **5**.

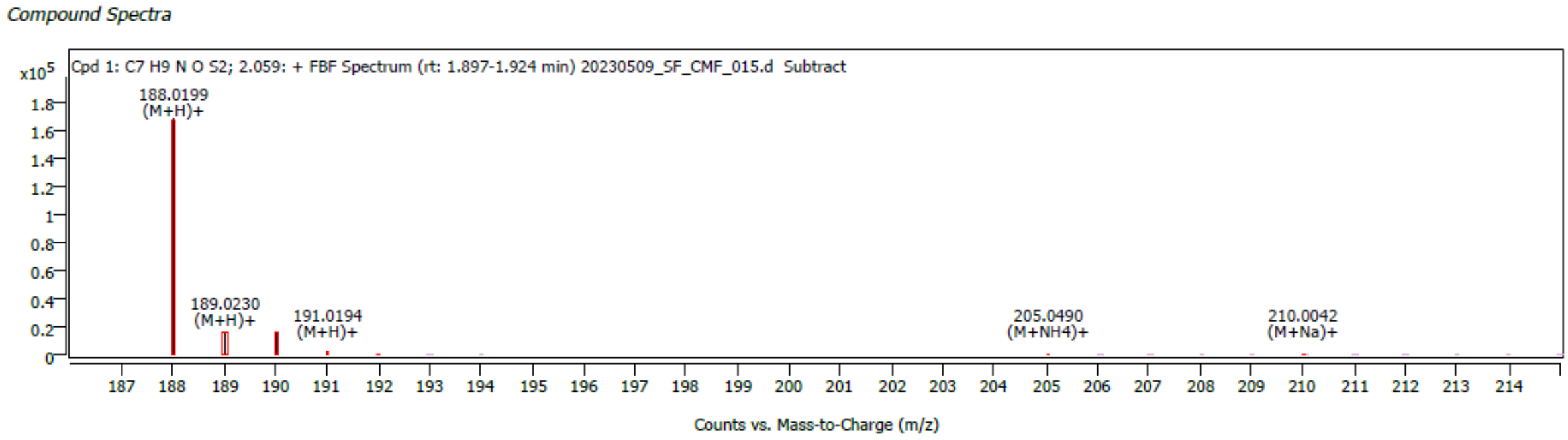

Figure S14C: LC/MS (ESI+) mass spectrum of **5**.

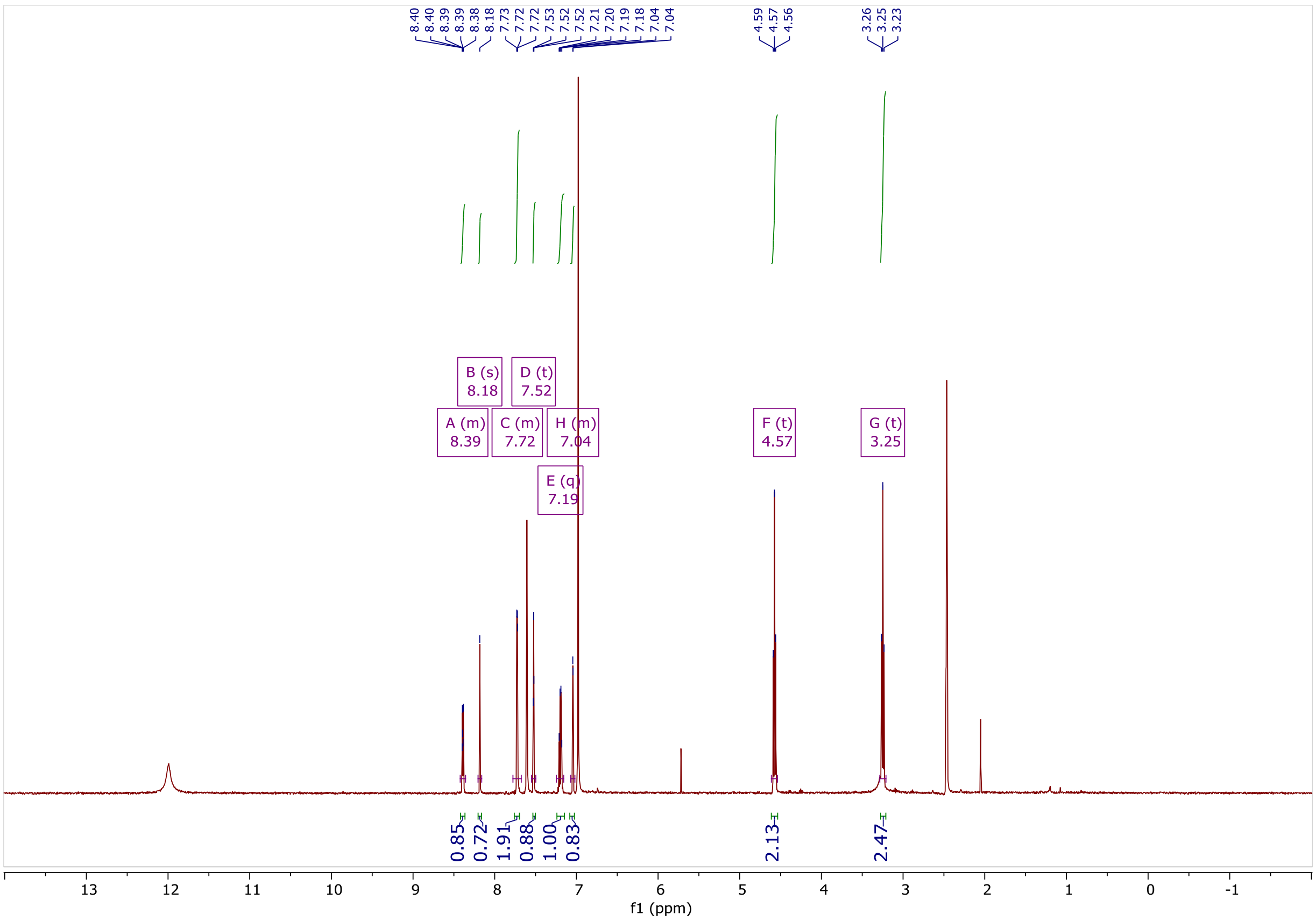

Figure S15A: ^{1}H NMR spectrum of **6**.

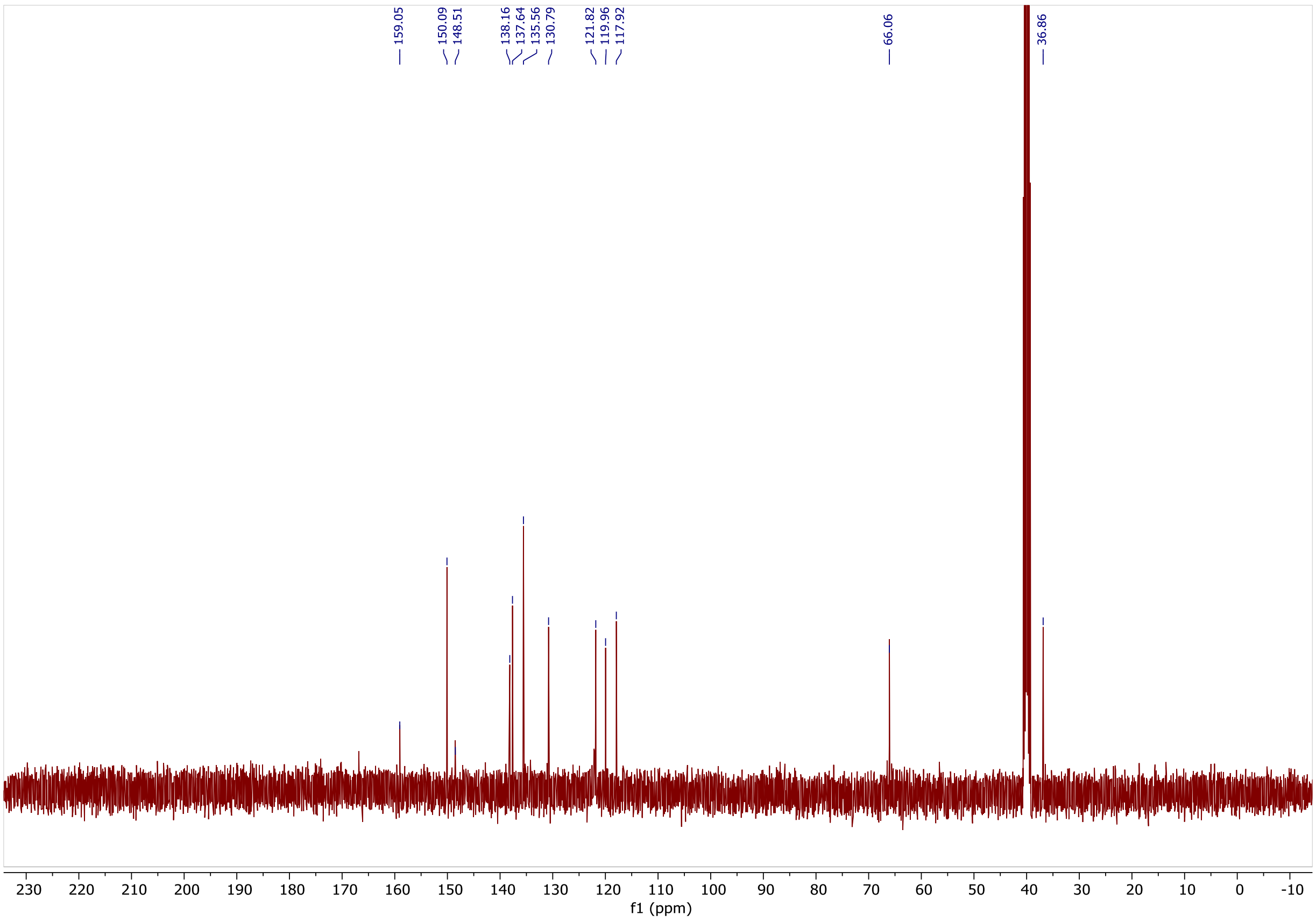

Figure S15B: ^{13}C NMR spectrum of **6**.

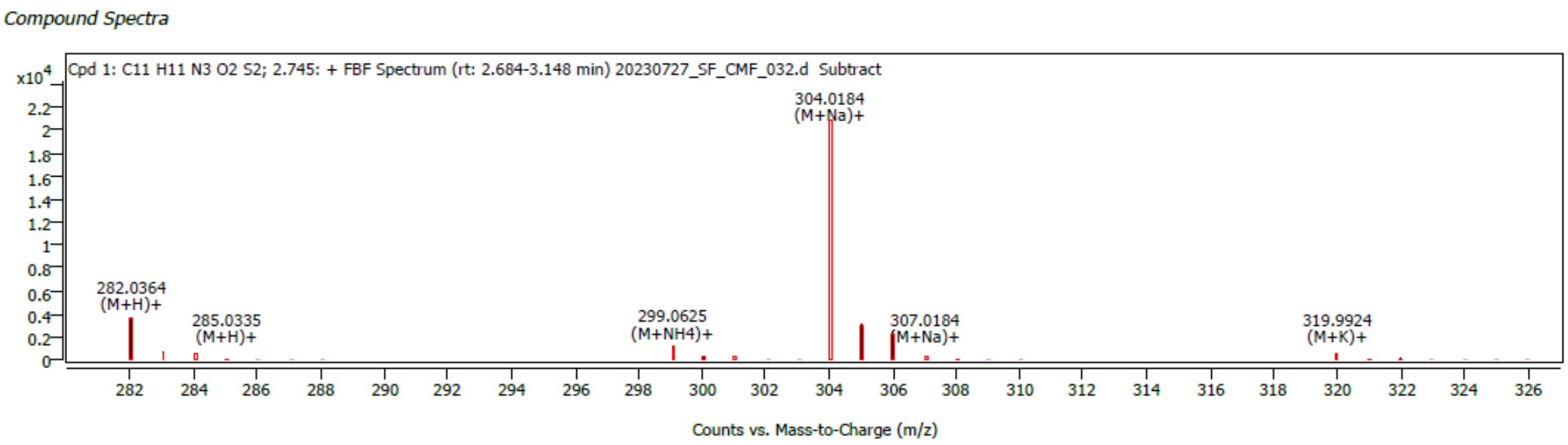

Figure S15C: LC/MS (ESI+) mass spectrum of **6**.

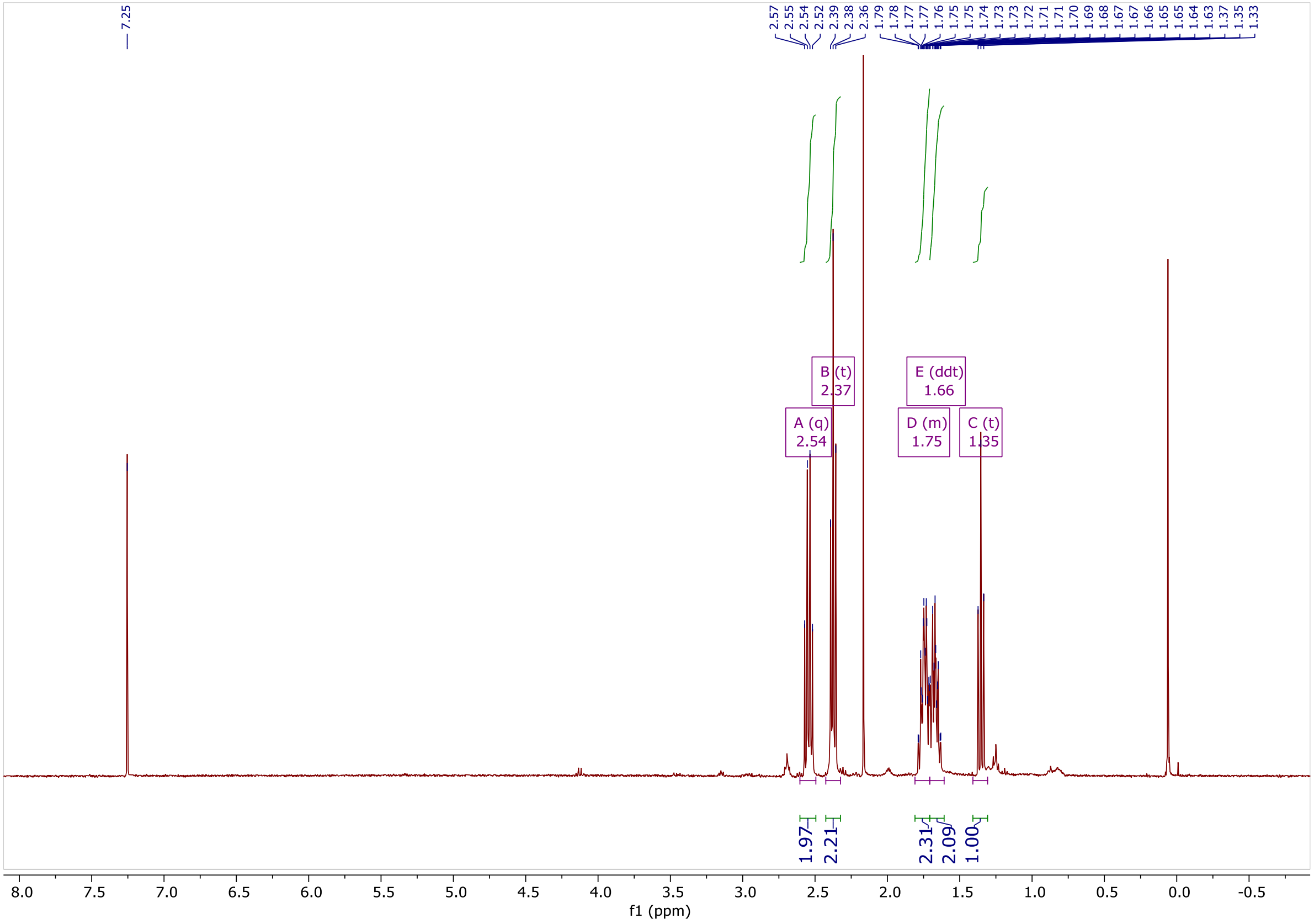

Figure S16A: ^{1}H NMR spectrum of **8**.

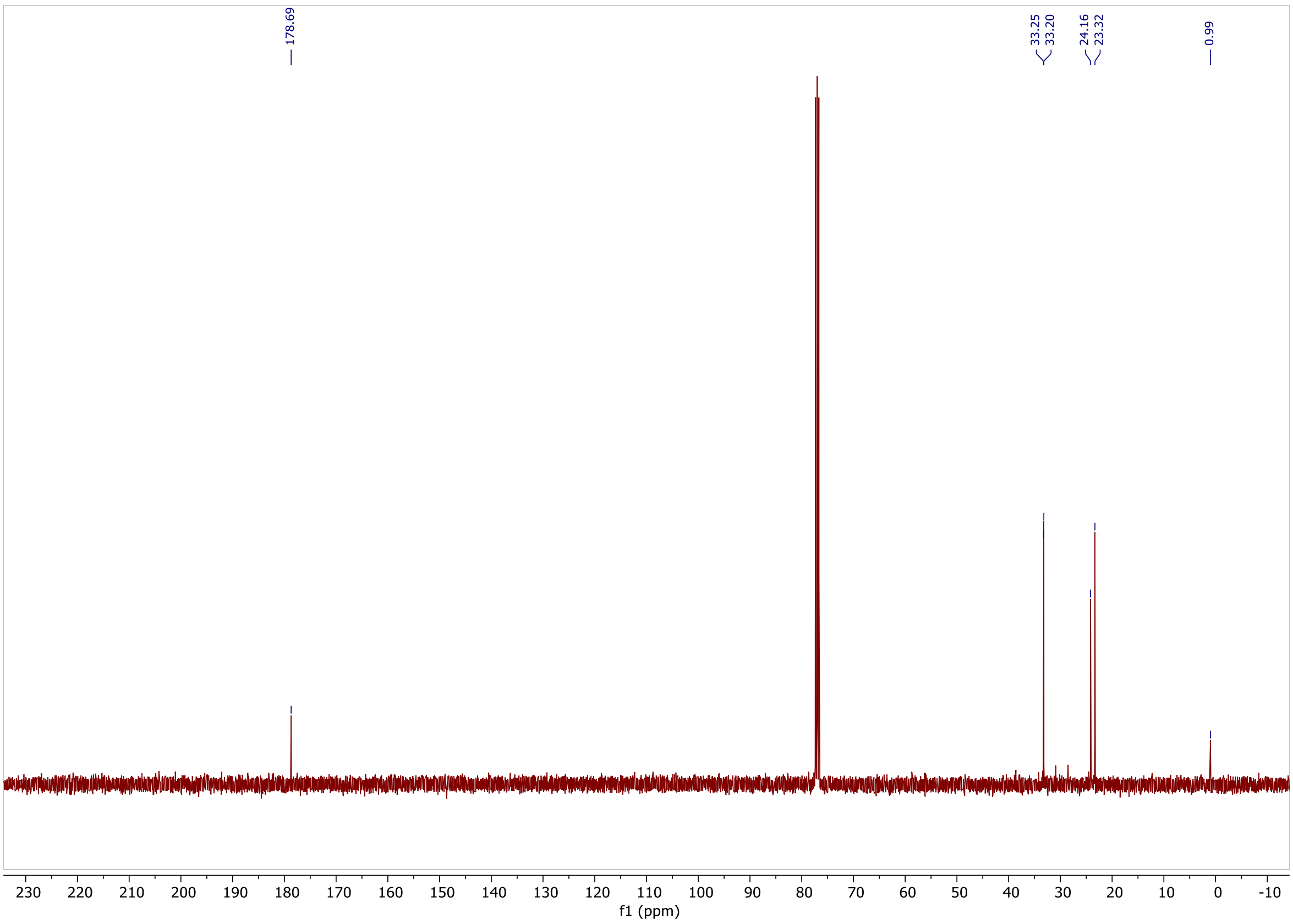

Figure S16B: ^{13}C NMR spectrum of **8**.

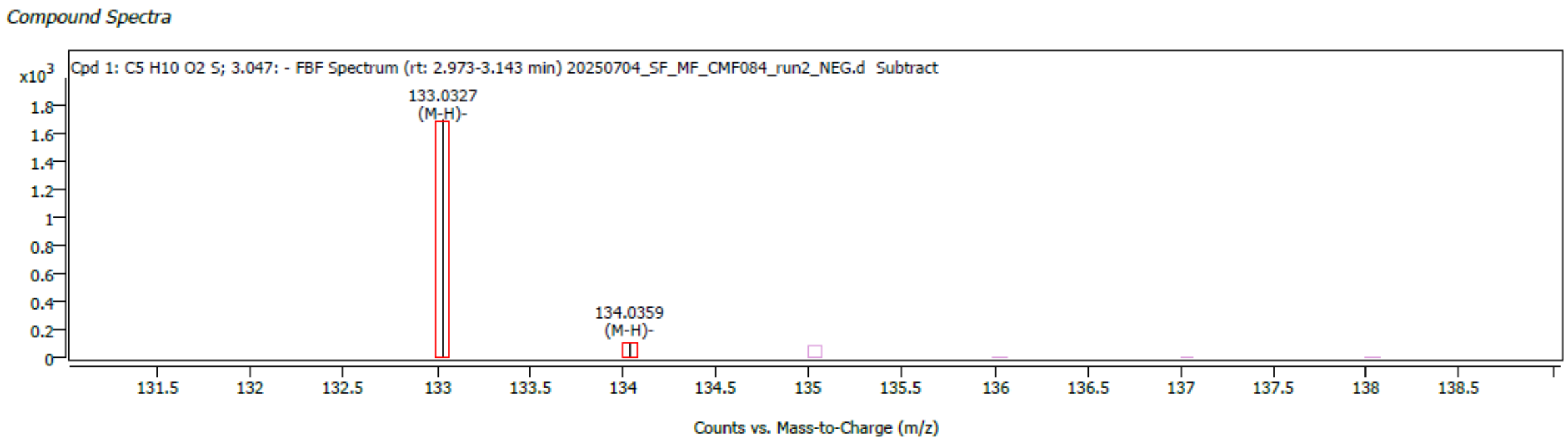

Figure S16C: LC/MS (ESI+) mass spectrum of **8**.

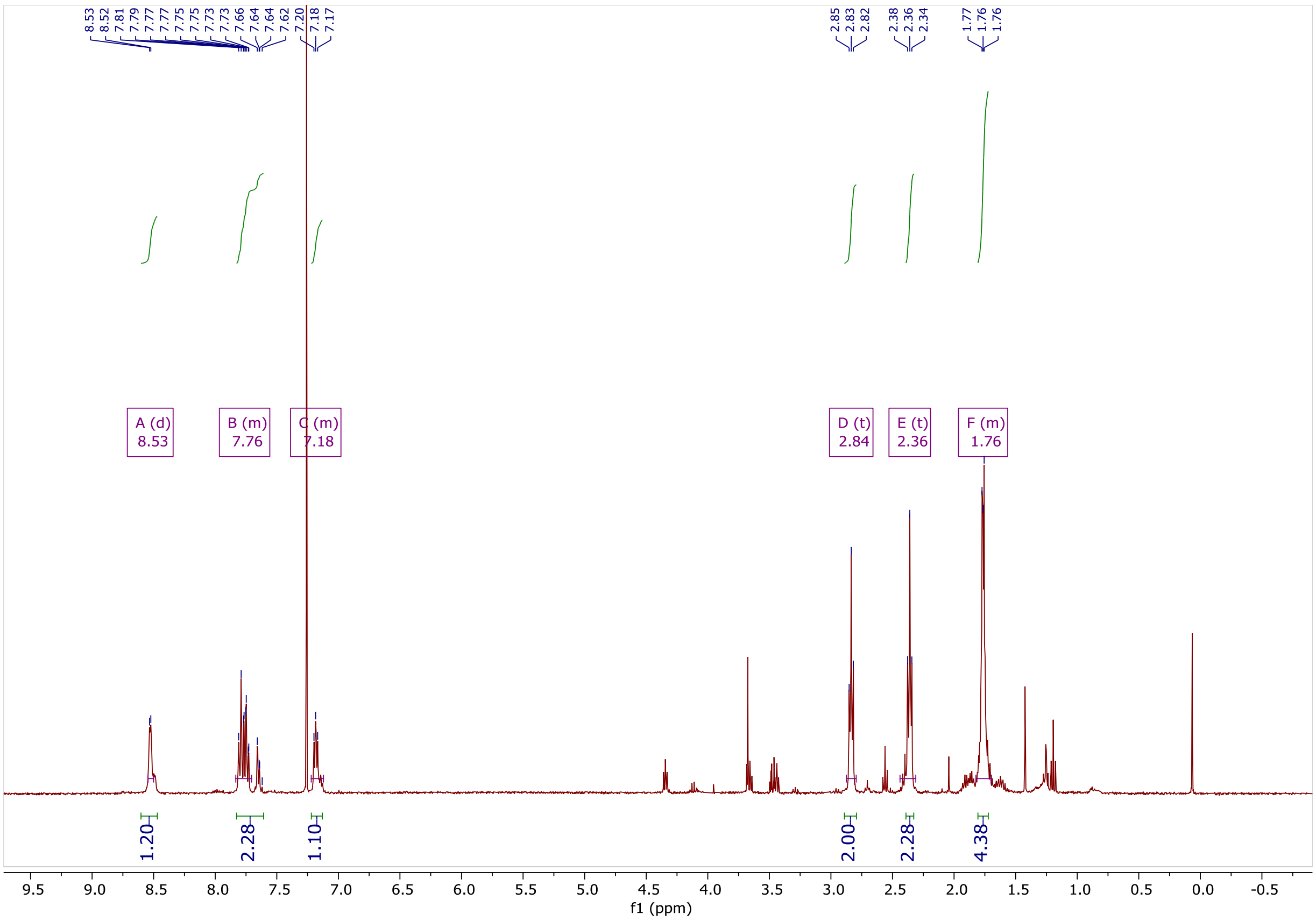

Figure S17A: ^{1}H NMR spectrum of **9**.

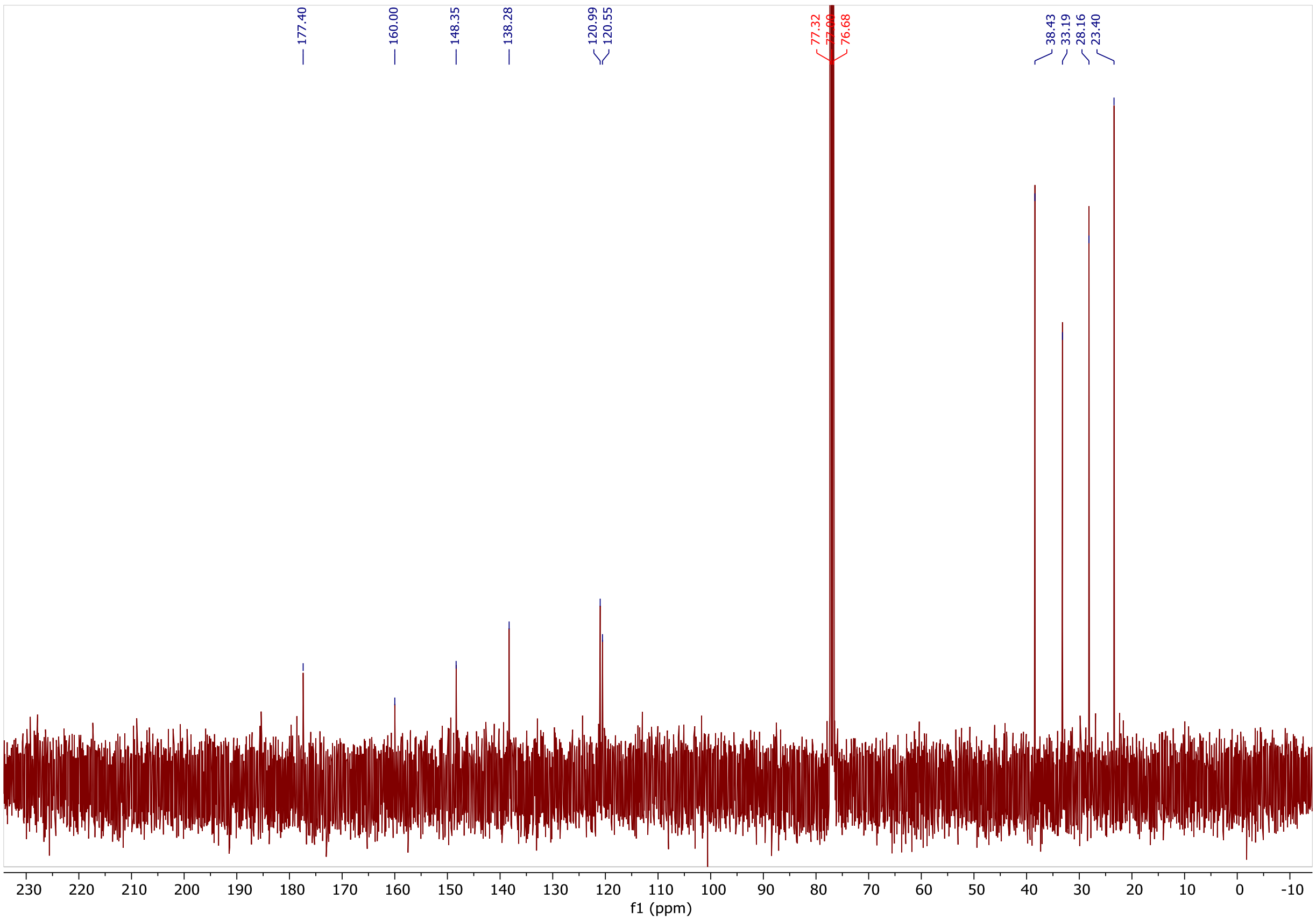

Figure S17B: ^{13}C NMR spectrum of **9**.

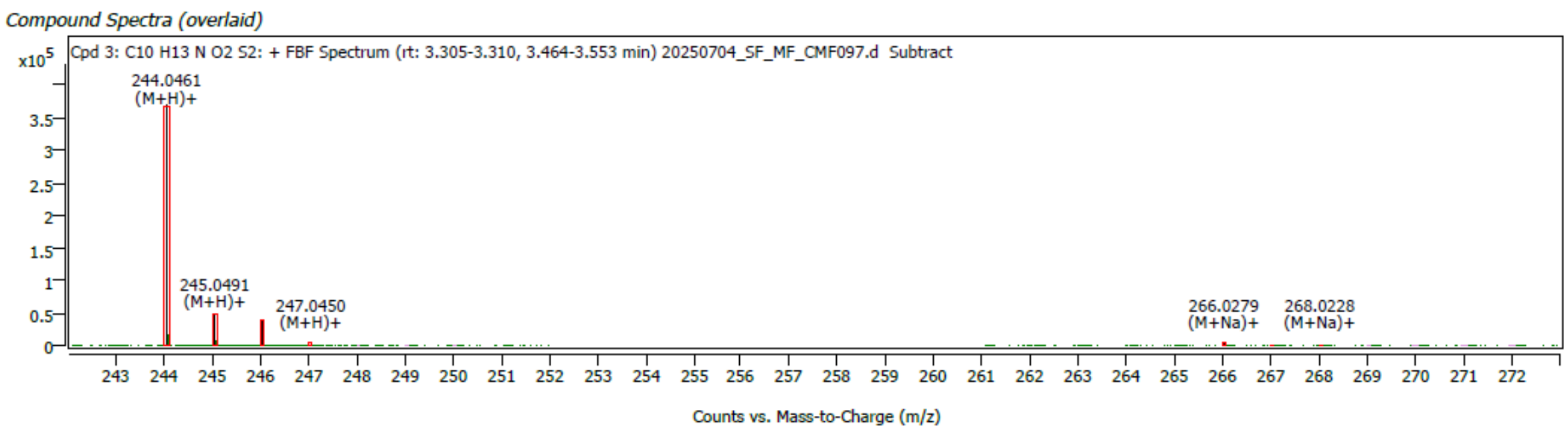

Figure S17C: LC/MS (ESI+) mass spectrum of **9**.

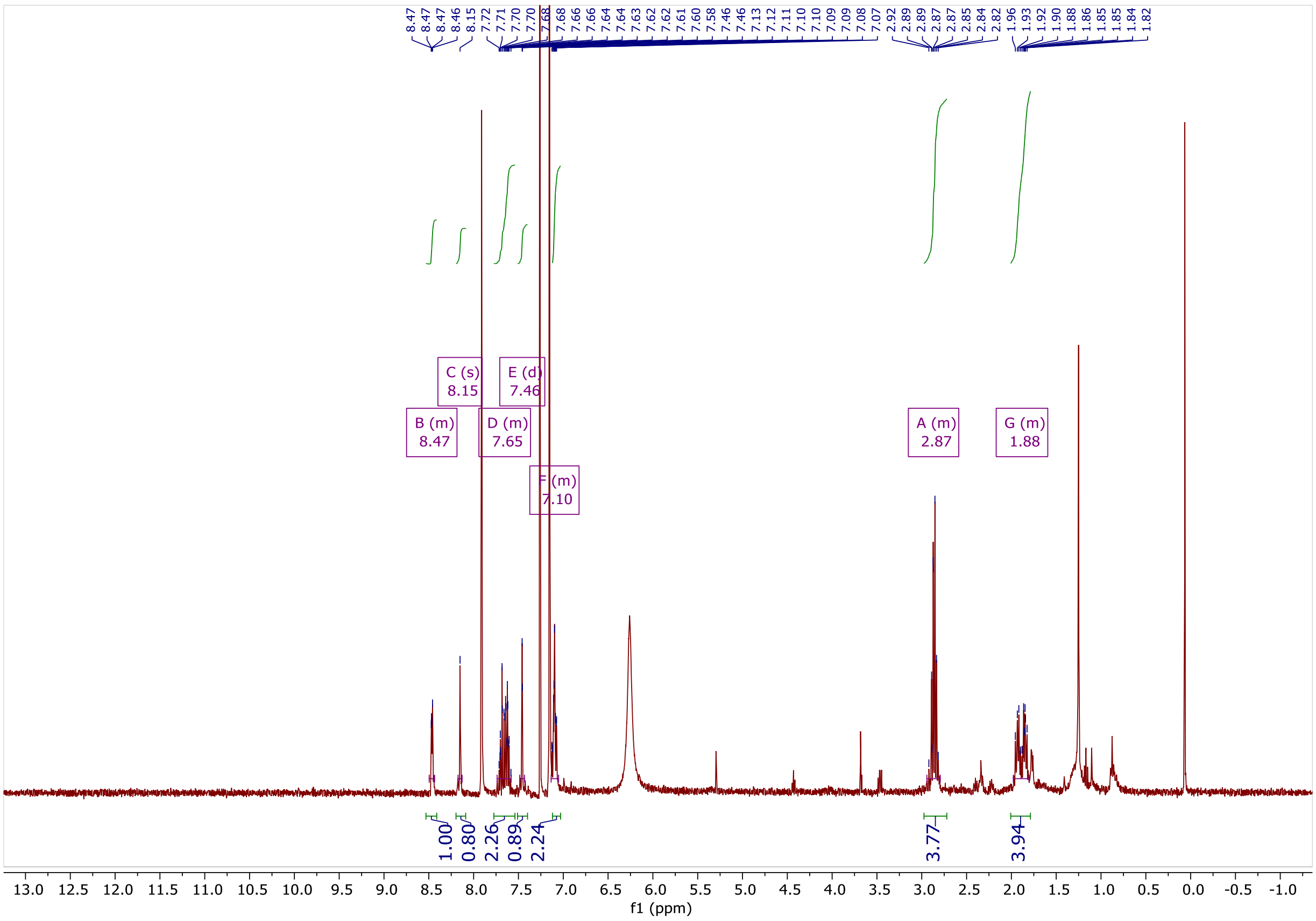

Figure S18A: ^{1}H NMR spectrum of **10**.

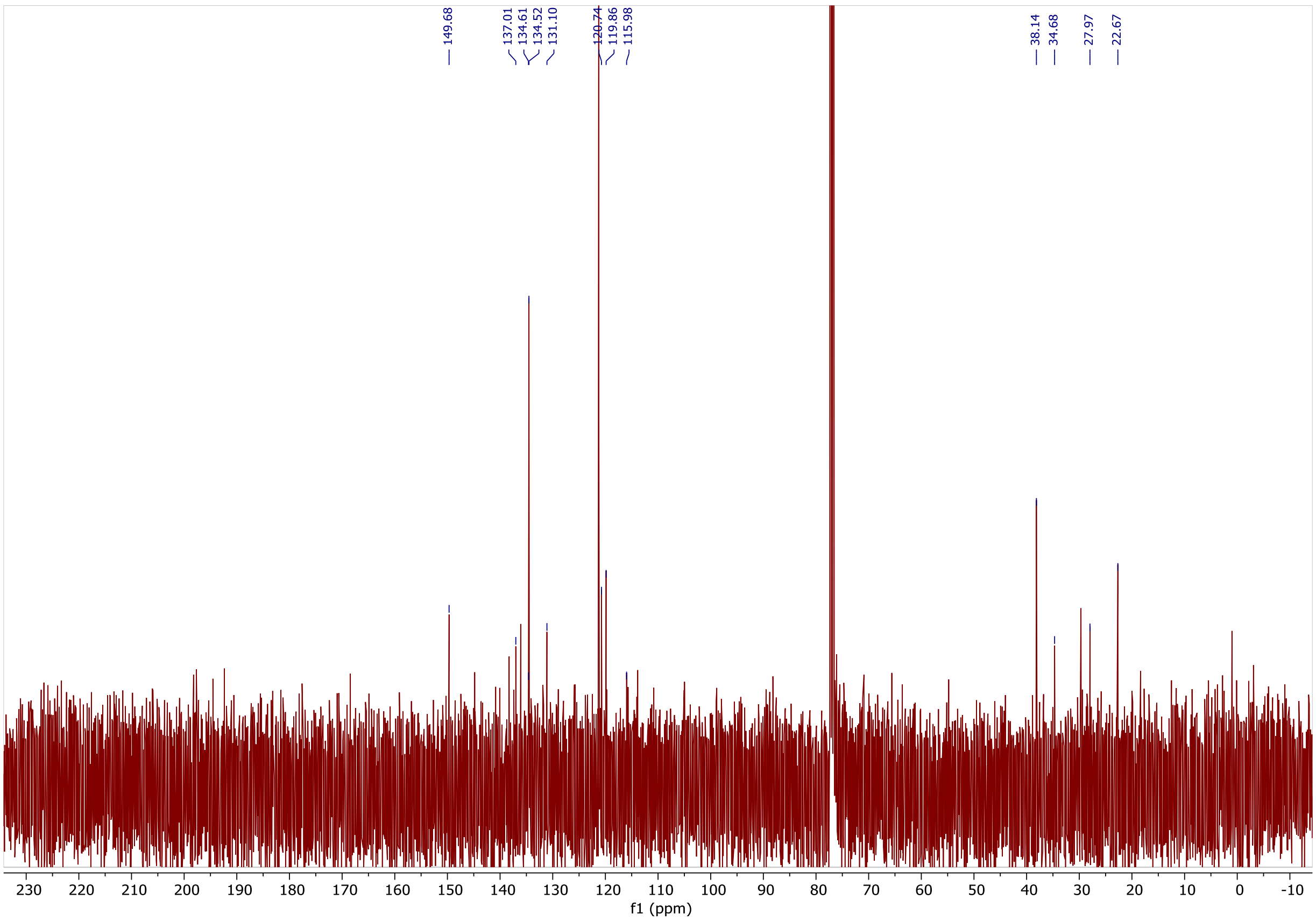

Figure S18B: ^{13}C NMR spectrum of **10**.

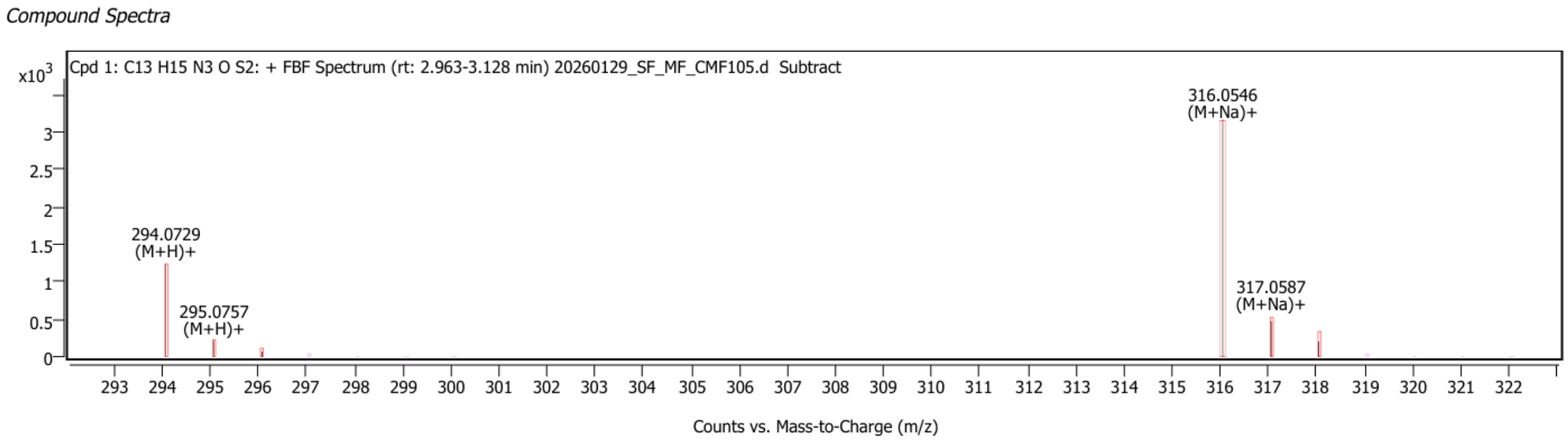

Figure S18C: LC/MS (ESI+) mass spectrum of **10**.

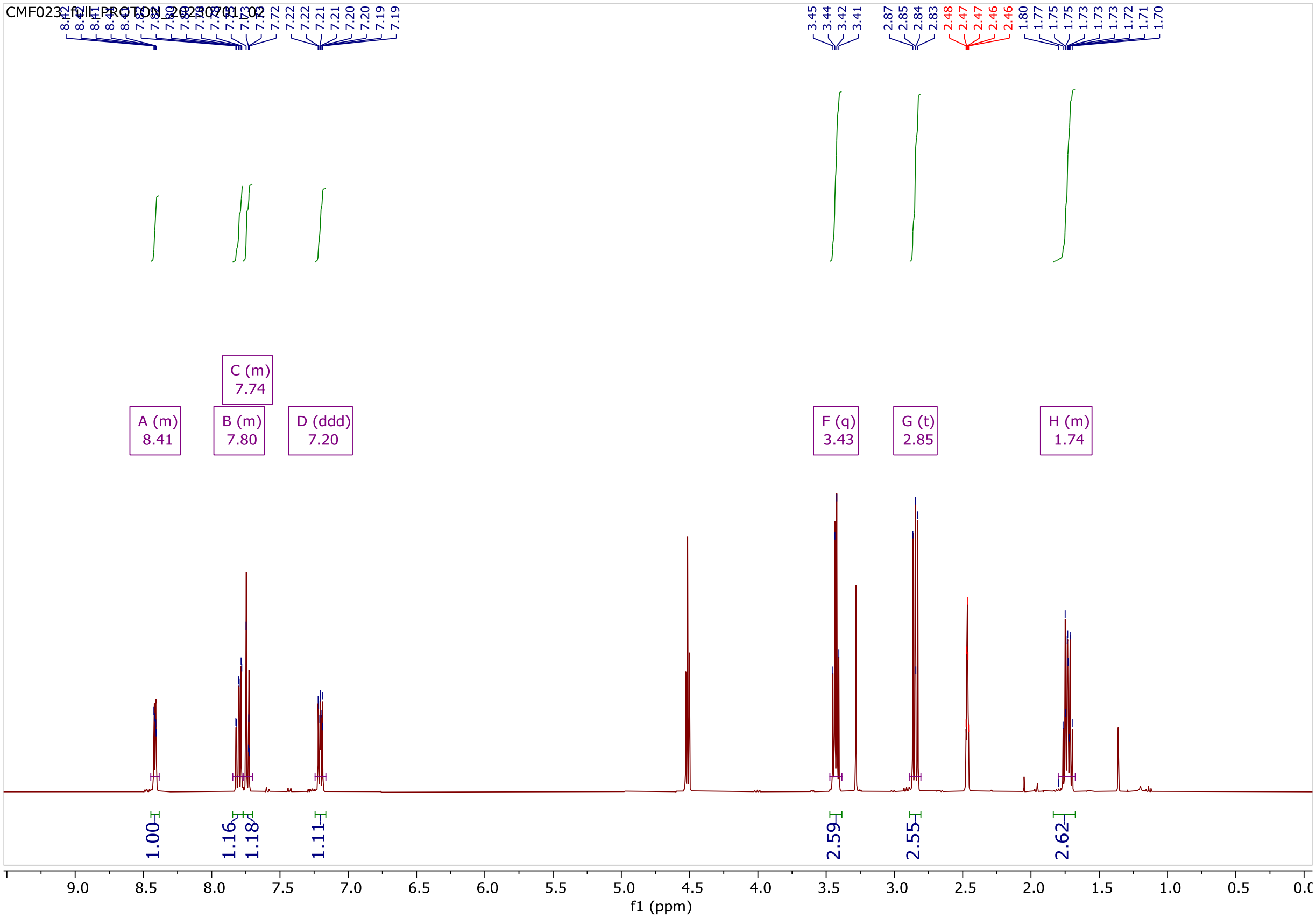

Figure S19A: ^{1}H NMR spectrum of **13**.

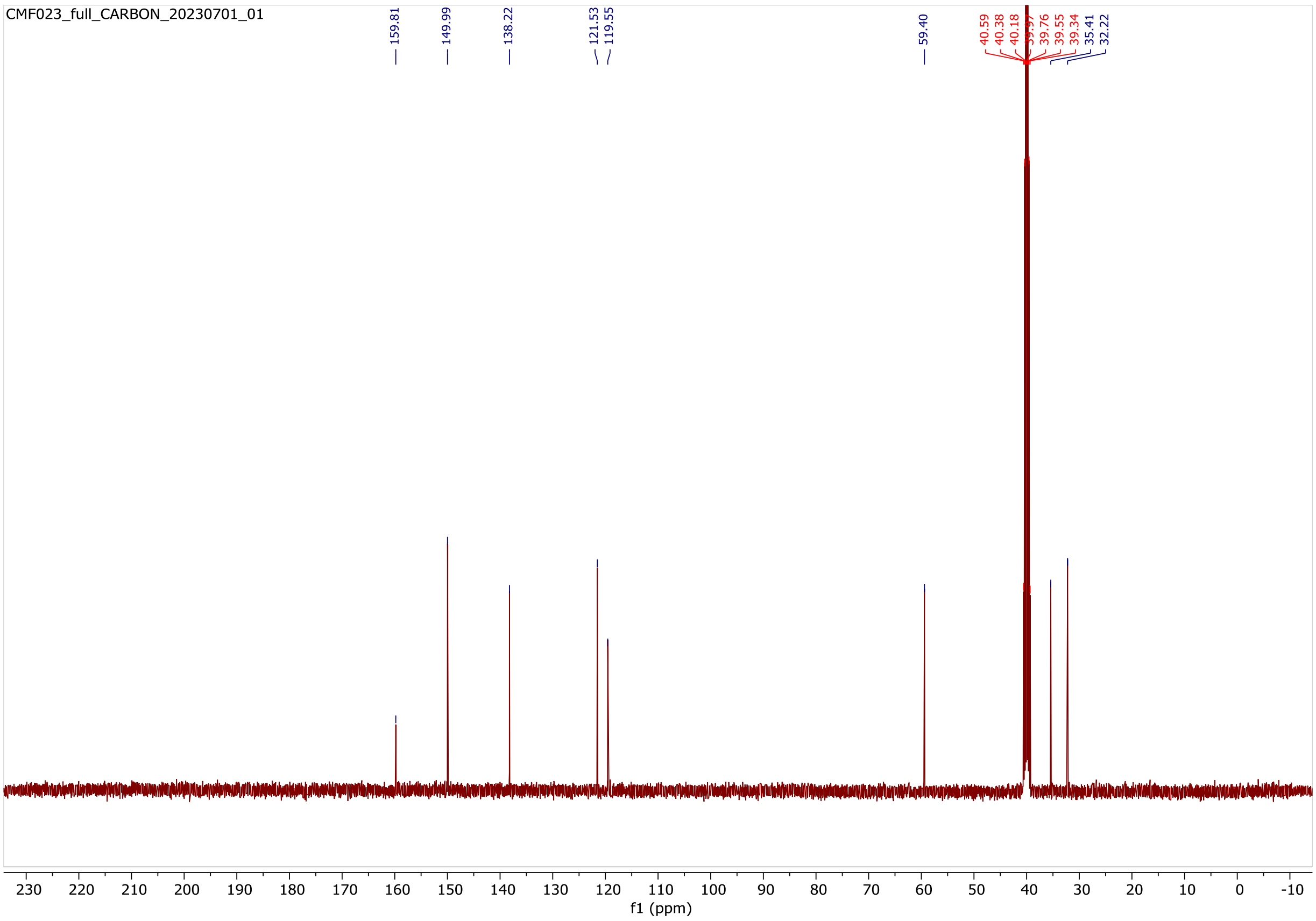

Figure S19B: ^{13}C NMR spectrum of **12**.

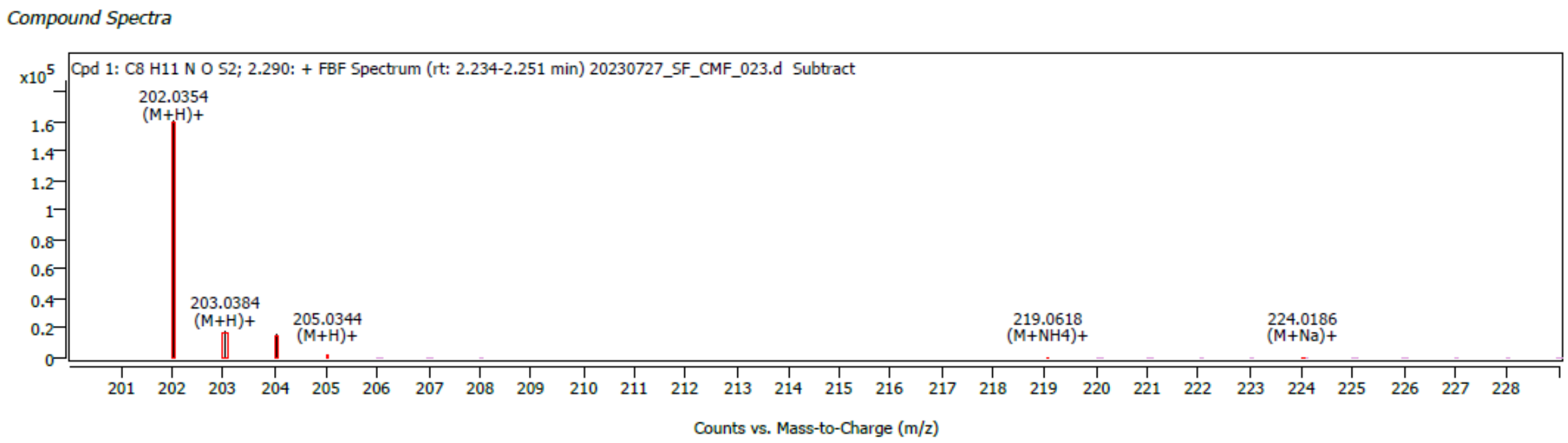

Figure S19C: LC/MS (ESI+) mass spectrum of **12**.

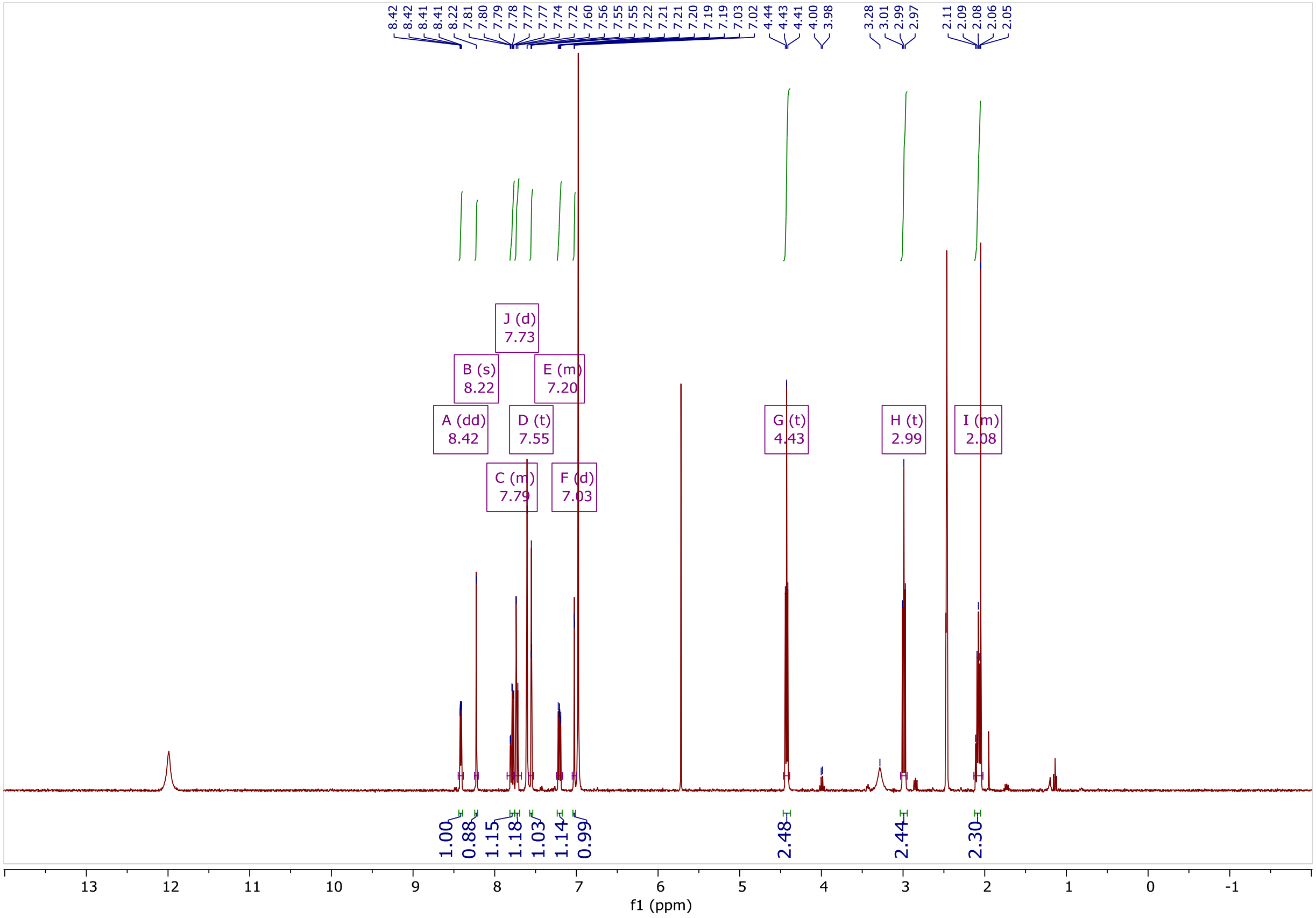

Figure S20A: ^{1}H NMR spectrum of **13**.

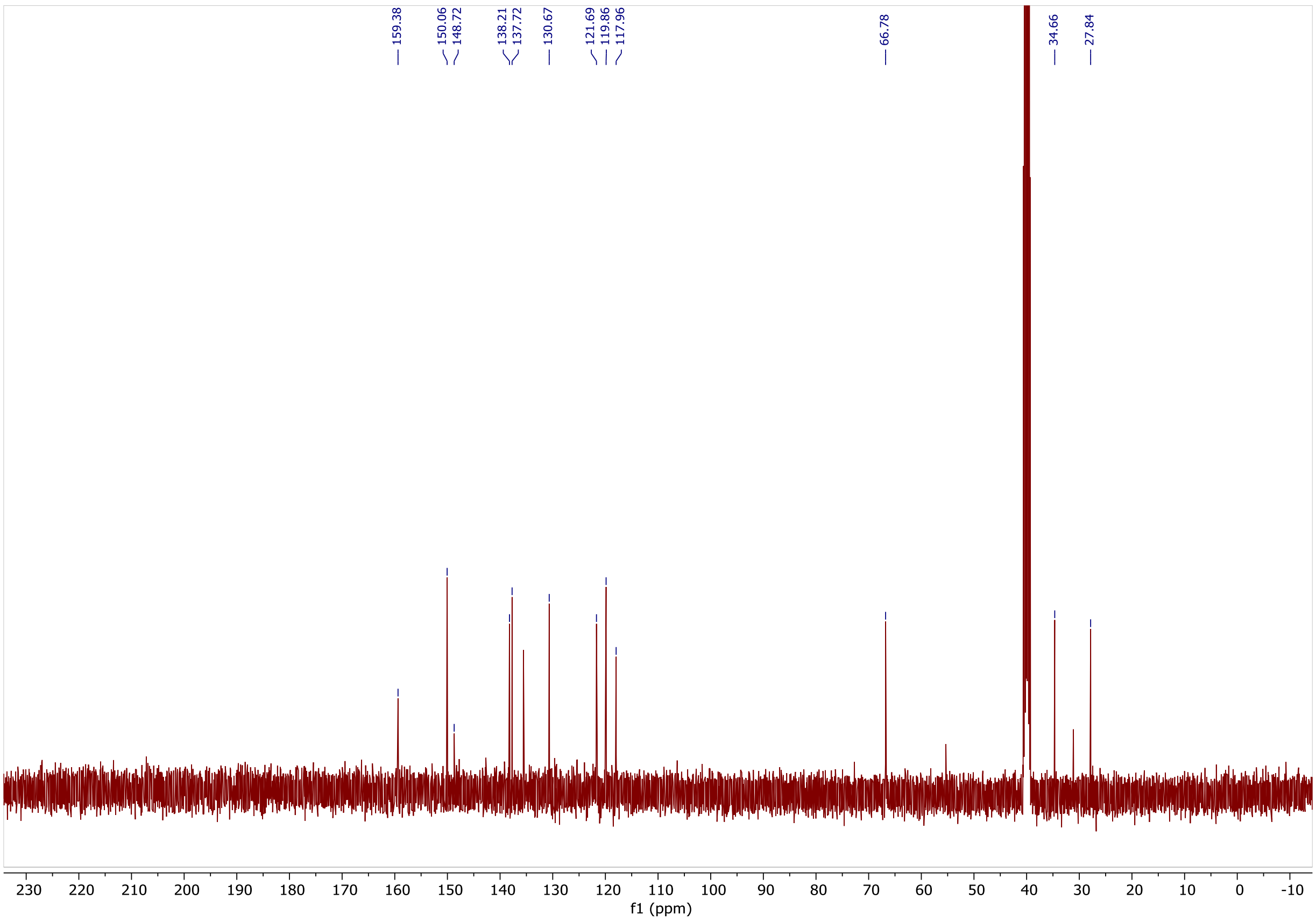

Figure S20B: ^{13}C NMR spectrum of **13**.

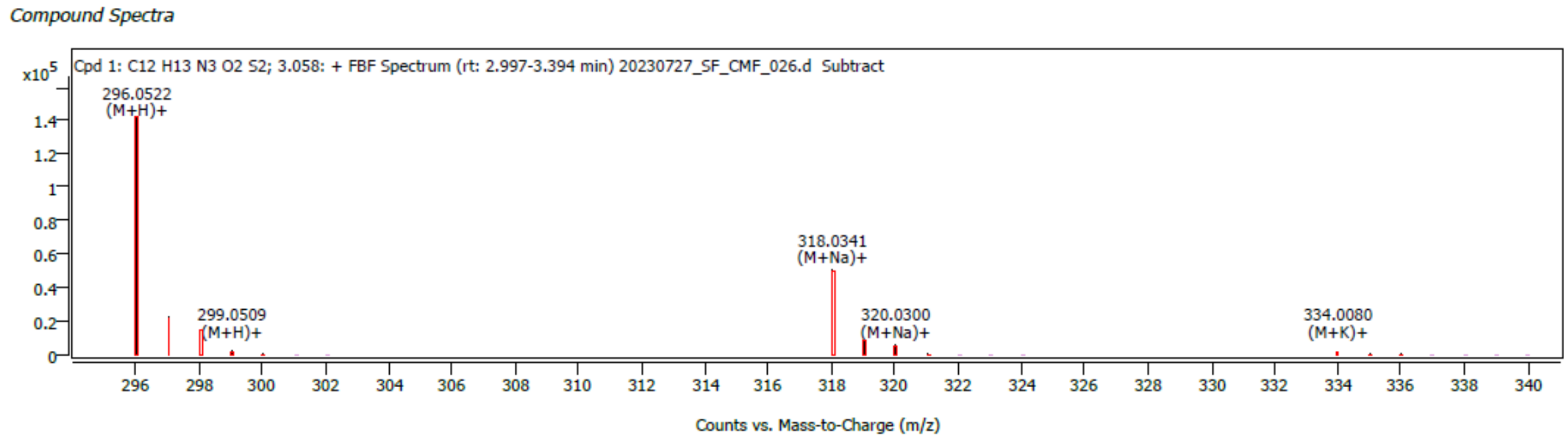

Figure S20C: LC/MS (ESI+) mass spectrum of **13**.

References:

[59] J. Deacon, K. Wang, D. Engelman, D. Hoyer, A. Wangweerawong, W. Hungerford, V. Sabbasani, D. Spiegel (Yale University), US20210085796A1, (2018).

[60] Y. Zhang, J. Wang, C. Liu, H. Xing, Y. Jiang, X. Li, "Novel disulfide bond bridged 7-ethyl-10-hydroxyl camptothecin-undecanoic acid conjugate/human serum albumin nanoparticles for breast cancer therapy" *J. Mater. Chem. B* **2023**, *11*, 2478–2489.

[61] L. R. Jones, E. A. Goun, R. Shinde, J. B. Rothbard, C. H. Contag, P. A. Wender, "Releasable Luciferin−Transporter Conjugates: Tools for the Real-Time Analysis of Cellular Uptake and Release" *J. Am. Chem. Soc.* **2006**, *128*, 6526–6527.

[62] X. Zhang, X. Zhang, L. Sun, F. Liu, M. Wang, J. Peng, Y. Wang, L. Ma, B. Wang, H. Wei, "Fabrication of biocleavable shell cross-linked hybrid micelles for controlled drug release using a reducible silica monomer" *Chem. Commun.* **2018**, *54*, 13495–13498.

[63] A. Rühling, K. Schaepe, L. Rakers, B. Vonhören, P. Tegeder, B. J. Ravoo, F. Glorius, "Modular Bidentate Hybrid NHC-Thioether Ligands for the Stabilization of Palladium Nanoparticles in Various Solvents" *Angewandte Chemie International Edition* **2016**, *55*, 5856–5860.
